# Preference-based measures of health-related quality of life in Indigenous people: a systematic review

**DOI:** 10.1007/s11136-023-03499-7

**Published:** 2023-09-16

**Authors:** Lilla M. Roy, Aidan Neill, Kristen Swampy, Juliette Auger, Sandra M. Campbell, Susan Chatwood, Fatima Al Sayah, Jeffrey A. Johnson

**Affiliations:** 1 School of Public Health, University of Alberta, Edmonton, AB Canada; 2 Present Address: School of Nursing, Cape Breton University, Sydney, NS Canada; 3 NorQuest College, Edmonton, Canada; 4 John W. Scott Health Sciences Library, University of Alberta, Edmonton, Canada

**Keywords:** Preference-based measures, Health-related quality of life, Indigenous, Systematic review

## Abstract

**Purpose:**

In many countries, there are calls to address health inequalities experienced by Indigenous people. Preference-based measures (PBMs) provide a measurement of health-related quality of life and can support resource allocation decisions. This review aimed to identify, summarize, and appraise the literature reporting the use and performance of PBMs with Indigenous people.

**Methods:**

Eleven major databases were searched from inception to August 31, 2022. Records in English that (1) assessed any measurement property of PBMs, (2) directly elicited health preferences, (3) reported the development or translation of PBMs for Indigenous people, or (4) measured health-related quality of life (HRQL) using PBMs were included. Ethically engaged research with Indigenous people was considered as an element of methodological quality. Data was synthesized descriptively (PROSPERO ID: CRD42020205239).

**Results:**

Of 3139 records identified, 81 were eligible, describing psychometric evaluation (n = 4), preference elicitation (n = 4), development (n = 4), translation (n = 2), and HRQL measurement (n = 71). 31 reported ethically engaged research. Reports originated primarily from Australia (n = 38), New Zealand (n = 20), USA (n = 9) and Canada (n = 6). Nearly all (n = 73) reported indirect, multi-attribute PBMs, the most common of which was the EQ-5D (n = 50).

**Conclusion:**

A large number of recent publications from diverse disciplines report the use of PBMs with Indigenous people, despite little evidence on measurement properties in these populations. Understanding the measurement properties of PBMs with Indigenous people is important to better understand how these measures might, or might not, be used in policy and resource decisions affecting Indigenous people. (Funding: EuroQoL Research Foundation).

**Supplementary Information:**

The online version of this article (10.1007/s11136-023-03499-7) contains supplementary material, which is available to authorized users.

## Plain English summary

Preference-based measures (PBMs) are surveys that help us understand the quality of life related to health of people or groups. These surveys are essential for making decisions about healthcare approaches and health policies. It is crucial that the PBMs accurately and consistently assess health for a person or group. Currently we lack a summary of information on how PBMs are used specifically for Indigenous people and whether these surveys effectively capture their health experiences. This is particularly important because Indigenous communities worldwide have unique knowledges and approaches to healing that promote their health and care experiences. To address this gap, we reviewed published information to understand how PBMs are used with Indigenous people. Our study shows that PBMs have been used more and more with Indigenous people even though there is not much information on how well these surveys work in this group. This finding highlights the need for further discussions and research on how to measure health-related quality of life accurately and meaningfully for Indigenous people.

## Introduction

Preference-based measures provide a measurement of health-related quality of life (HRQL) that can be used to evaluate individual or population health. PBM allow us to calculate value or utility of different health states, which are used in calculating quality-adjusted life years (QALYs) used in economic evaluations. There are two types of PBMs: direct and indirect. Direct PBMs, such as time trade-off or standard gamble, typically ask respondents to make choices about hypothetical health scenarios [1] under conditions of certainty or uncertainty. On the other hand, indirect PBMs use a classification system describing specific dimensions of health, and a scoring system, to infer preferences for a particular health state based on previously elicited values usually from the general population [1]. Indirect generic PBMs (such as the EQ-5D-5L) permit comparison across different populations, whereas indirect condition-specific PBMs (such as the cancer-specific EORTC-QLQ-C30) can be more useful in disease-specific healthcare areas [1].

The usefulness of a PBM is partially contingent on the appropriateness of the PBM for the population in which it is used. Many Indigenous groups hold a worldview and conceptualization of health [2] that may not be reflected in current instruments that have been translated or adapted to non-Indigenous contexts. Indigenous people face distinct health needs and experience significant health inequalities [3–6], often stemming from the negative impacts of colonial systems [7]. There is a need to improve appropriateness of health care services and measures of disease for Indigenous populations [6]. This means ensuring that PBMs consider the specific cultural contexts, values, and health perspectives of Indigenous communities. It is not only an ethical imperative but also a priority for population health to establish the validity and reliability of PBMs in Indigenous populations.

There is limited evidence on the use of PBMs with Indigenous people, and their alignment with Indigenous worldviews. A systematic review of literature conducted in 2016 [8] identified only one PBM—the EQ-5D 3-level version (EQ-5D-3L) which has been preliminarily validated for use in Māori in New Zealand [9]. Upon conducting a more recent and expanded scoping review (March 2020), we identified an additional seven studies using PBMs with Indigenous people. These studies had various aims including measurement of HRQL, validation of PBMs, and direct valuation of health states [10–16]. We felt it pertinent to conduct an updated and expanded systematic review on the use of PBMs with Indigenous people, globally.

The objective of this review was to identify, summarize, and appraise the literature reporting the use of direct or indirect PBMs with Indigenous people in terms of (1) assessing measurement properties of PBMs, (2) eliciting health preferences using a direct PBM, (3) reporting development or translation of a PBM for Indigenous Peoples, or (4) measuring HRQL using a PBM.

## Methods

### Search strategy

Our database search was executed by an expert health librarian (SMC) using the following databases: PROSPERO, OVID Medline, OVID EMBASE, OVID Global Health, OVID Health and Psychosocial Instruments, OVID PsycInfo, Cochrane Library (CDSR and Central), EBSCO CINAHL, EBSCO Econlit, Proquest Dissertations and Theses Global and SCOPUS. The search used controlled vocabulary (e.g., MeSH, Emtree, etc.) and key words representing the concepts “Indigenous people” and “preference-based measures”. The search strategies were informed by the work of Goodwin and Green [17]. Variants of several University of Alberta Health Sciences Search filters were modified for use in each database [18–21]. No additional limits were applied to the searches. Databases were searched from inception to May 2021, and updated in August 31, 2022. Results (3139) were exported to COVIDENCE review management software. Duplicates (947) were removed. Detailed search strategies are available as an online resource (Supplementary Information).

### Screening of records

Records were included in our review if they met the following criteria: (a) the purpose of the study aligned with one of the four categories identified in the objectives; (b) the sample included Indigenous people as the primary population of interest or identified Indigenous people as a specific sub-group; (c) reported the use of any direct (such as time trade-off or standard gamble) or indirect (including generic or condition specific) PBM; (d) were published in English; and (e) were published after inception of PBMs (1980). During the screening process, the reviewers considered the definition of Indigenous people as provided by the authors of each study. If the definition appeared outdated or ambiguous, the screening decisions were guided by the definitions of Indigenous people provided by the United Nations [22, 23] and the Government of Canada [24].

To capture a comprehensive range of literature, reports were retained even if they originated from the same study, so long as they reported on a different category of interest. For example, measurement or performance. However, if two publications reported on the same aspect of the study (for example, a published abstract followed by a full paper), only the most recent or complete publication was included. Registered trials and published abstracts were included only if there was no subsequent peer reviewed publication available. In cases where abstracts provided limited information, data was extracted from the corresponding Registered Trials where available. All titles, abstracts, and full texts were independently reviewed for inclusion by two reviewers (LMR and AN). Decisions were reached through discussion and by consensus, and any disagreements were reviewed with a senior researcher.

### Data extraction and synthesis

Data was extracted by the first author (LMR). For publications where the first reviewer had uncertainties, a second reviewer (AN during initial review or KS during update) independently extracted data to validate the initial assessment. Missing or unclear data was recorded as “Not Reported” or “Unclear”. The screening process was facilitated by Covidence software, and the extracted data was compiled using MS Office Excel. Included publications were categorized according to the objectives of the review, and data was synthesized descriptively. It is important to note that the evaluation of measurement properties was based on publications with samples primarily consisting of Indigenous peoples.

The first author (LMR) worked with an Indigenous Elder (EJA) and an Indigenous research assistant (KS) who contributed to the interpretations and conclusions of the review. The Elder and Indigenous research assistant also advised on relevancy, strength-based language (writing in a good way), and future directions for research in this area. A further description of author position in relation to this work is available as an online resource (Supplemental Information).

### Assessment of methodological quality

Given the objective of this review to describe the extent of study and use of PBMs with Indigenous people, it was not relevant to assess methodologic quality for all report types. The focus was on describing the frequency and purpose of PBM use rather than evaluating methodological rigor. However, ethically engaged research with Indigenous people was considered an indicator of quality. Ethical approaches to research involving or pertaining to Indigenous people is well established, whereby many institutions and regions have culturally and ethically appropriate research practices [25–28]. When assessing for ethical engagement with Indigenous people, we considered: (a) whether the publication reported some form of patient-oriented, community-oriented, Indigenous-centered, or otherwise engaged approach to research, or (b) whether the publication reported ethics approval from an Indigenous ethics committee. The presence of either of these elements was categorized as an “ethically engaged approach”. Of note, the Aboriginal and Torres Strait Islander quality assessment tool [29] was recently developed in Australia; as it has yet to be validated in global contexts, we chose not to use it in this review.

## Results

### PRISMA summary

**Fig. 1:**
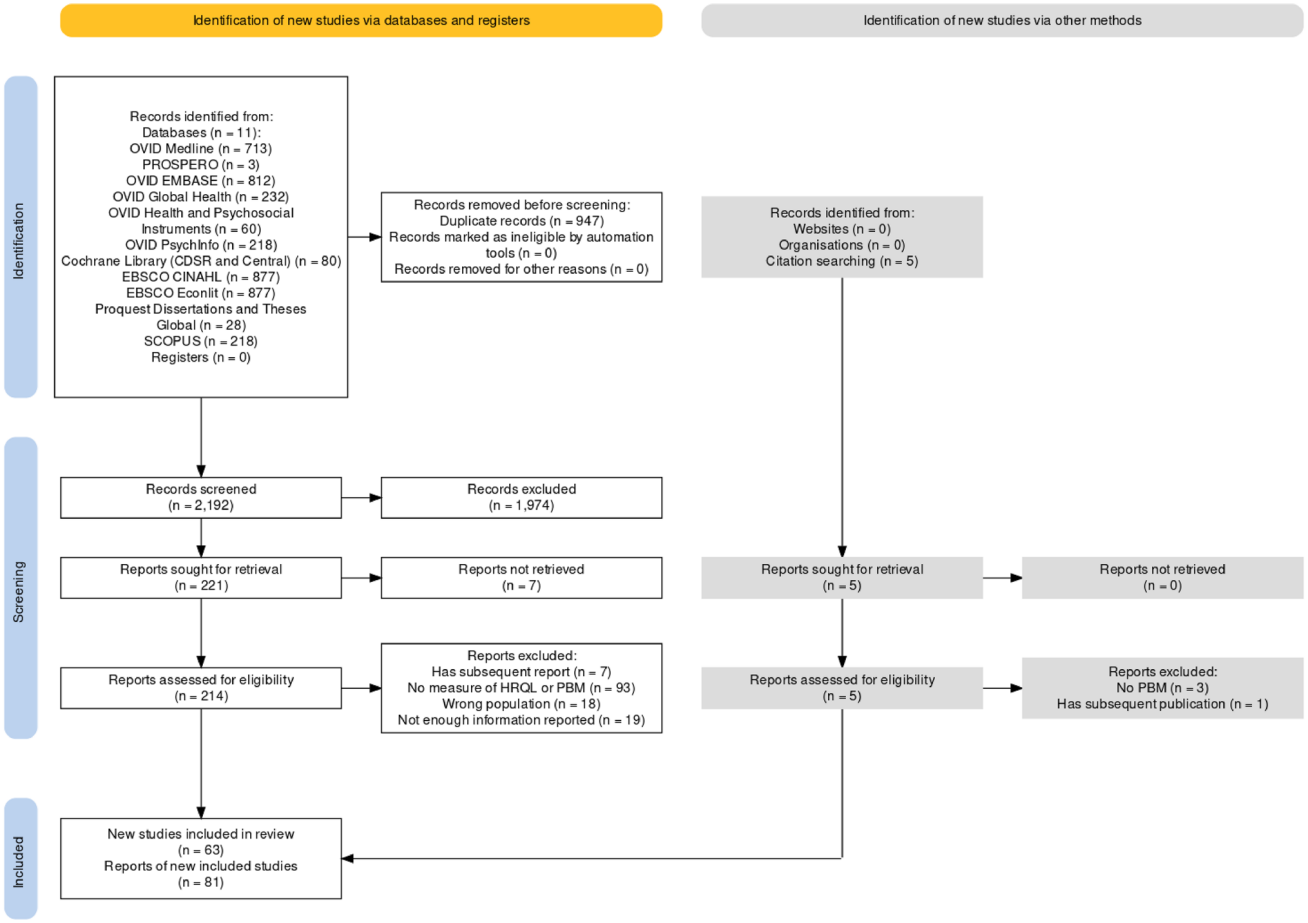
PRISMA flow diagram. Figure generated using: Haddaway et al., [122]

This review includes 81 reports that met the inclusion criteria, originating from 63 unique studies. A PRISMA diagram is included in Fig. 1 PRISMA Flow Diagram. There has been a significant increase in reported use of PBMs with Indigenous people over the past five years, with 47 reports since 2018. Among these, the majority were peer-reviewed journal articles of original research (n = 45) [9, 11, 12, 14–16, 30–69] with an additional 9 registered trials [70–78], 1 pilot study [79], 11 abstracts [80–88], 1 dissertation [89], and 12 research protocols [10, 90–97].

### Geographical region

The majority of publications reporting the use of PBMs with Indigenous people were from Australia (n = 38) and New Zealand (n = 20). There were fewer reports from the USA (n = 9) and Canada (n = 6). There was a single multinational study, and several other studies from South Africa (n = 3), Ecuador (n = 1), and Mexico (n = 2) (Table 1).

**Table 1:** Characteristics of included reports

First author	Year	Country	Indigenous group	Ethically engaged approach?	Results reported for Indigenous people or subgroup?	Direct or indirect PBM?	Instrument or method
*Reports of measurement of health-related quality of life*							
Altomare	2018	USA	Native American, Pacific Islander	NR	No	Indirect	EORTC-QLQ-C30
Armstrong	2021	Australia	Aboriginal	Yes	Yes	Indirect	EQ-5D-3L
Barnabe	2015	Canada	Aboriginal Albertans	NR	Yes	Indirect	EQ-5D
Barnabe	2018	Canada	First Nations, Inuit, Metis people	Unclear	Yes	Indirect	EQ-5D
Banham	2019	Australia	Aboriginal South Australians	NR	Yes	Indirect	SF-6D v2
Chenhall	2012	Australia	Indigenous Australians	Yes	Yes	Direct	SEIQoL-DW
Cousins	2021	New Zealand	Maori and Pacific peoples	Yes	Unclear	Indirect	EQ-5D-5L
Custer	2014	USA	American-Indian	NR	Yes	Indirect	EQ-5D
Delbaere	2021	Australia	Aboriginal and Torres Strait Islander	Yes	Yes	Indirect	EQ-5D-5L
Derrett	2009	New Zealand	Maori	Yes	Yes	Indirect	EQ-5D
Derrett	2011	New Zealand	Maori	NR	No	Indirect	EQ-5D
Derrett	2012	New Zealand	Maori, Pacific	NR	Yes	Indirect	EQ-5D-3L
Derrett	2017	New Zealand	Maori, Pacific	NR	Yes	Indirect	EQ-5D-3L
Dingwall	2019	Australia	Indigenous Australians	NR	Yes	Indirect	EQ-5D-5L
Dingwall	2020	Australia	Indigenous Australians	NR	Yes	Indirect	EQ-5D
Dingwall	2021	Australia	Indigenous Australians	Yes	Yes	Indirect	EQ-5D
Donaldson	2022	Australia	Aboriginal and Torres Strait Islander	Yes	Yes	Indirect	EQ-5D-5L
du Toit	2016	South Africa	South Africans	NR	Yes	Indirect	EORTC-QLQ-C30
Farace	2014	USA	Native American	NR	No	Indirect	EORTC-QLQ-C30
Gall	2022	Australia	Aboriginal and Torres Strait Islander	Yes	Yes	Indirect	AQ0L-4D
Garvey	2014	Australia	Indigenous Australian	NR	Yes	Indirect	AQoL-4D
Garvey	2016	Australia	Indigenous Australian	NR	Yes	Indirect	AQoL-4D
Groessl	2003	USA	Native American	NR	No	Indirect	QWB
Guevara	2020	Equador	Saraguro	Yes	Yes	Indirect	EQ-5D-3L
Hachem	2021	Australia	Indigenous Australians	NR	Yes	Indirect	EQ-5D-5L
Harcombe	2022	New Zealand	Maori	Unclear	Yes	Indirect	EQ-5D-5L
Hatcher	2011	New Zealand	Maori	Yes	Yes	Indirect	EQ-5D
Hay	2021	USA	Native Hawaiian/Pacific Islander, American Indian/Alaskan Native	NR	Yes	Indirect	EQ-5D-5L
Hughes	2004	South Africa	Zhosa	NR	Yes	Indirect	EQ-5D
Ingham	2017	New Zealand	New Zealand Maori	Yes	Yes	Indirect	EQ-5D-Y (Proxy 1 version)
Jamieson	2022	Australia	Indigenous children	NR	Yes	Indirect	CHU-9D
Janda	2009	Australia	Indigenous Australian	NR	No	Indirect	FACT-GP
Johnson	2015	Australia	Aboriginal and Torres Strait Islander	Yes	Yes	Indirect	AQoL
Ju	2021	Australia	Indigenous Australian	Yes	Yes	Indirect	EQ-5D-5L
Kilkenny	2018	Australia	Aboriginal Australians	NR	Yes	Indirect	EQ-5D-3L
Kinchin	2018	Australia	Aboriginal and Torres Strait Islander	NR	NR	Indirect	EQ-5D-5L; AQOL-8D
Kularatna	2020	Australia	Indigenous Australian	Yes	Yes	Indirect	CHU-9D
LaGrappe	2022	Australia	Aboriginal Australians	Yes	Yes	Indirect	EQ-5D
Lalloo	2015	Australia	Indigenous Australian children	Unclear	Yes	Indirect	CHU-9D
Lapsley	2020	New Zealand	Maori	Yes	Yes	Indirect	EQ-5D-3L
Lavergne	2009	Canada	Aboriginal (Canada)	NR	Yes	Indirect	HUI3
Lavergne	2012	Canada	First Nations, Inuit, or Metis	NR	Yes	Indirect	HUI3
Liu	2010	Australia	Indigenous Australian	Yes	Yes	Indirect	EQ-5D
Lott	2019	Australia	Aboriginal and Torres Strait Islander	NR	NR	Indirect	EQ-5D
Maclennan	2022	New Zealand	Maori	NR	Yes	Indirect	EQ-5D-3L
Mann	2020	Australia	Indigenous Australian	NR	Yes	Indirect	EQ-5D
McDermott	2015	Australia	Aboriginal and Torres Strait Islander	Unclear	Yes	Indirect	AQoL
McNoe	2019	New Zealand	Maori, Pacific People	NR	Yes	Indirect	EQ-5D-3L
Mold	2004	USA	Native American	NR	No	Indirect	HUI3; QWB
Moodie	2010	Australia, New Zealand, Fiji, Tonga	Multiple (Maori, Pacific Islanders, Indigenous Fijians, Tongans, and unspecified "Australians")	NR	Yes	Indirect	AQoL-6D
Morales	2018	Mexico	Maya-Yucateco Indigenous	NR	Yes	Indirect	EQ-5D-3L
Oen	2018	Canada	Canadian Aboriginal	NR	Yes	Indirect	QoML
Radford	2019	Australia	Aboriginal and Torres Strait Islander	NR	Yes	Indirect	EQ-5D
Ramirez-Cervantes	2015	Mexico	Mexican Mestizo	NR	Yes	Indirect	EQ-5D-3L
Ralph-Campbell	2006	Canada	Aboriginal	NR	Yes	Indirect	HUI-3
Ranta	2021	New Zealand	Maori	NR	Yes	Indirect	EQ-5D-3L
Robinson	2022	Australia	Aboriginal	Unclear	Yes	Indirect	AQoL-4D
Sato	2012	Ghana	Multiple traditional groups identified through religion (traditionalist African or Indigenous religion) or other ethnicities	NR	No	Indirect	EQ-5D-3L
Scholes-Robertson	2021	Australia	Aboriginal Australian and/or Torres Strait Islander	NR	Unclear	Indirect	EQ-5D
Segal	2016	Australia	Indigenous Australian	Unclear	Yes	Indirect	AQoL-4D
Shettigar	2021	New Zealand	Maori and Pacific peoples	Unclear	Yes	Indirect	EQ-5D-3L
Smith	2019	Australia	Aboriginal Australian and/or Torres Strait Islander	Unclear	Yes	Indirect	EQ-5D-5L
Taylor	2005	New Zealand	Maori	NR	Yes	Indirect	EQ-5D
Thompson	2022	New Zealand	Maori and Pacific peoples	NR	Yes	Indirect	EQ-5D-3L
Toombs	2018	Australia	Aboriginal and Torres Strait Islander	Yes	Yes	Indirect	AQoL-8D
Walker	2019	New Zealand	New Zealand Maori	Yes	Yes	Indirect	EQ-5D
Walker	2021	New Zealand	Maori or whanau of Maori	NR	Yes	Indirect	EQ-5D
Wilson	2010	USA	American Indian	Yes	Yes	Indirect	QWB
*Reports of instrument development or translation*							
Anderson	2021	Australia	Aboriginal and Torres Strait Islander	Yes	Yes	NA	NA
Arrow	2018	Australia	Aboriginal Australians	Yes	Yes	Indirect	CHU-9D, EQ-5D-Y, new oral specific health utility scale
Howard	2020	Australia	Aboriginal and Torres Strait Islander	Yes	Yes	NA	NA
Nagel	2020	Australia	Aboriginal and Torres Strait Islander	Yes	Yes	Indirect	EQ-5D-3L
Willing	2020	New Zealand	Maori	Yes	Yes	NA	NA
*Reports of direct preference elicitation*							
Alfonso	2022	USA	American Indian/Alaskan Natives	Yes	Yes	Direct	VAS (0-100)
Devlin	2000	New Zealand	Maori	NR	Yes	Indirect	EQ-5D-3L
Ju	2021	Australia	Indigenous Australians	Yes	Yes	Direct	Standard gamble
Ju	2021	Australia	Indigenous Australians	Yes	Yes	Direct	Standard gamble
*Reports of instrument performance*							
McClure	2011	USA	American Indian/Alaskan Natives	NR	Yes	Indirect	NEI-VFQ-25
Jelsma	2004	South Africa	Zhosa	NR	Yes	Indirect	EQ-5D
Perkins	2004	New Zealand	Maori	Yes	Yes	Both	EQ-5D-3L (indirect); VAS (0-100) (direct)
Ribeiro-Santiago	2021	Australia	Aboriginal and Torres Strait Islander	Yes	Yes	Indirect	EQ-5D-5L

*SEIQoL-DW* Self evaluated individual quality of life-direct weight, *QOML* Quality of My Life Questionnaire, *USA *United States of America

### Indigenous groups

At least 13 different Indigenous groups were identified. These included (as reported) Native American, American Indian, Native Hawaiian, Alaskan Native, Pacific Islander, First Nations (of Canada), Inuit, Metis people (of Canada), Māori (or New Zealand Māori), Indigenous Australians, Aboriginal and Torres Strait Islander, Indigenous Fijians, Tongans, Saraguara People of Ecuador, and Xhosa (Table 1). In some cases, the specific ethnicity beyond “Aboriginal” or “Indigenous” was not provided, however, the country is clearly stated. Among the included reports, only 26 described the methods used to determine ethnicity in the sample, such as self-report, or health records [10–12, 35, 42, 45, 48–50, 53, 55, 57, 61, 63, 69, 73, 75, 89, 95, 98–100].

### Indirect and direct preference-based measures

Among the included reports, 37 identified the use of a PBM as their primary or one of their primary outcomes (Table 1); 38 reported it as a secondary outcome, and 6 were unclear or did not specify. The majority (n = 73) reported using indirect multi-attribute PBMs. Out of these, most (n = 69) reported the use of generic PBMs. Four publications reported the use of a direct PBM, with two utilizing standard gamble [65, 66], and the other two [33, 58] using the Visual Analogue Scale (VAS) to directly elicit health preferences. Overall, only 17 reported the use of a VAS, with 8 affirming the scale as 0–10 and the rest using a 0–100 scale.

The most commonly reported generic PBM was the EQ-5D (n = 50). Among these, 19 did not specify the versions used, while 17 used the EQ-5D-3L, 11 used the EQ-5D-5L, and 3 used the EQ-5D-Y. Other generic PBMs reported included the Child Health Utility 9-D (CHU-9D) (n = 4), Assessment of Quality of Life (AQoL) (n = 8), QWB (n = 3), Short Form Six-Dimension (SF-6D) v2 (n = 1), and the Health Utility Index Mark 3 (HUI3) (n = 4). Four condition-specific PBMs were reported in 6 publications—the National Eye Institute Visual Function Questionnaire-25 (NEI-VFQ-25) (n = 1), the Functional Assessment of Cancer Therapy-General Population (FACT-GP) (n = 1), the European Organization for the Research and Treatment of Cancer Quality of Life Questionnaire (EORTC-QLQ-C30) (n = 3), and a newly proposed oral-specific health utility scale (n = 1). Only 14 reports clearly stated which value set was used [11, 38, 43, 44, 54, 55, 73, 81, 82, 90, 93, 95, 101, 102], including value sets from Australia (n = 3), Canada (n = 1), New Zealand (n = 5), UK (n = 3), and USA (n = 2).

### Purpose for using the PBM

Among the identified reports (Table 1), four examined the performance of a PBM [9, 41, 47, 52], four directly elicited health preferences [13, 58, 65, 66], four explored the development of a PBM [10, 64, 103, 104], two explored translation of a PBM [35, 68], while a majority (n = 71) used a PBM to measure health status. In some instances, reports could be classified in more than one category of use. For example, a research protocol from Australia aimed to develop a dental specific health utility scale (including preference elicitation) and measure dental HRQL within the same study. Among the included reports, 25 described interventional designs, 38 were observational, 5 were psychometric evaluations, 4 were economic evaluations, and 4 were longitudinal designs.

Nine of the included reports used a qualitative component or approach [30, 60, 64, 73, 78, 90, 102–105], with all but one [64] being part of a mixed methods study. Five reported using mixed methods, qualitative methods, or interviews but did not specify further the specific methodology used [60, 72, 78, 102, 103]. Of the remaining reports, one was a study protocol and described the plan to use phenomenological and collaborative storytelling to explore lived experience of injury-related disability [90]. A second report used phenomenology to explore the lived experience of asthma and assess a “whanau-centered, culturally tailored, health literacy-based intervention” among Māori in New Zealand [73]. A third publication described anthropological fieldwork with participant observation and interviews to contextualize their quality of life measure [30]. Howard et al. [104] published a protocol to develop a PBM for Indigenous Australians which planned to use Yarning Circles and semi-structured interviews. Three reports [64, 103, 104] described the use of Indigenous or decolonizing methodologies alongside other study methodology. For example, Willing et al. [64] reported a qualitative design situated within a Kaupapa Maori theoretical paradigm.

#### Performance of PBMs in Indigenous people

Four publications explored psychometric evaluation of a PBM specific to Indigenous people. Among these, 3 explored validity [9, 41, 52], including content validity [9, 52], discriminant validity [52], concurrent validity [41, 52], or convergent validity [52]. All four explored some form of reliability, including test–retest reliability [9, 41] and internal consistency [47, 52]. These studies were conducted in Australia [52], South Africa [41], New Zealand [9], and the USA [47].

Three of these studies used the EQ-5D [9, 41, 52]. The South African study found that the formally translated EQ-5D demonstrated both reliability and validity for Xhosa people [41]. Perkins (2004) suggested that the EQ-5D showed content validity but might lack construct validity among Māori people in New Zealand. The authors also suggested that the EQ-5D-3L had test–retest reliability with Māori people in New Zealand [9]. The most recent Australian study [52] concluded that the EQ-5D-5L demonstrated good concurrent validity, discriminant and convergent validity, and adequate internal consistency for Indigenous Australians. Only one publication explored a condition-specific based PBM, namely the NEI-VFQ-25 [47].

#### Direct preference elicitation with Indigenous people

We found two publications reporting the use of the standard gamble method with Indigenous people. One of these focused on population-based utility scores among Indigenous Australians for HPV infection and oropharyngeal squamous cell carcinoma [66], and the other reported population-based utility scores among Indigenous Australian women for HPV infection and cervical squamous cell carcinoma [65]. Both refer to one larger study around HPV. Two publications [9, 13] reported using data from a larger study on the valuation of health states with Māori people in New Zealand. In this study, valuations for health states were obtained using a visual analogue 0–100 scale (versus time trade-off or standard gamble). Similarly, a recent publication [58] used a VAS to value health states associated with suicide and depression. Although the VAS can be used as a direct measure of utility, it has a number of limitations [106]. Two publications reported obtaining individual direct preference weights using the Self-Evaluated Individual Quality of Life-Direct Weight (SEIQoL-DW) [30] and the Quality of My Life Questionnaire (QoML) [51].

#### Development of a PBM

We identified four reports that relate to the development of PBMs for Indigenous people. Arrow et al. (2018) published a protocol for a randomized controlled trial using a minimally invasive dentistry approach that proposed to simultaneously develop a dental specific child health utility scale for Aboriginal children in Australia [10]. Howard et al. (2020) also published a protocol to develop a new culturally relevant indirect, multi-attribute PBM including a descriptive system and underlying scoring algorithm for Aboriginal and Torres Strait Islanders in Australia [104]. Anderson et al. (2021) published an abstract reporting findings for the qualitative phase of this proposed study, finding a conceptual model of well-being including family, community, and culture that is being used to develop and score a well-being measure [103]. Willing et al. [64] describes a qualitative study situated within a Kaupapa Maori theoretical paradigm that specifically considers which key dimensions Western economic measures might miss, and to inform future development of a culturally-appropriate PBM. They report the need to “consider the individual within the context of the collective… and the environment” (p. 9).

#### Translation of a PBM

Translation was reported 9 times, of which three reported formal translations. Du Toit et al. [35] reported using the formal Xhosa and Afrikaans translation of the EORTC-QLQ-C30, a cancer-specific PBM. Jelsma et al. [41] reported using the Xhosa version of the EQ-5D (although not specified in the report, this would have been the three-level version, given that the five-level version was not yet available). The remaining studies suggested informal translation of the EQ-5D-3L to multiple Ghanaian dialects [53], the EQ-5D to Xhosa [39], and the AQoL to Creole [48]. Nagel et al. [105] reported forward translation of the EQ-5D-5L to multiple Australian Northern Territory languages to support a larger study [34, 91]. One report from New Zealand using the EQ-5D-3L indicated the use of “bilingual” translators to assist with a small proportion (11%) of interviews, but did not specify which language was used for translation (presumably Māori) [49].

### Ethically engaged research involving Indigenous people

An ethically engaged approach to research was detected in 31 reports. Within 8 reports there was insufficient information to generate an opinion, although wording suggested it may have been considered. Most reports (n = 42) did not provide information related to engagement, relationship-building, or Indigenous-specific ethical approaches to research. The reports of ethically engaged research increased over time, with approximately 75% reported since 2018. Indigenous members of the team (EJA, KS) emphasized the value of understanding whether studies were done in a good way, and the importance of relational, engaged approaches to future research.

## Discussion

Our review identified a substantial number of recent publications from diverse research areas that reported the use of PBMs with Indigenous people worldwide. This review also demonstrates that a wide variety of PBMs have been used to report health status, despite relatively little (or, in some cases, lack of) evidence on their performance in various Indigenous populations. Similarly, studies investigating translation or development of PBMs and preference elicitation were nearly absent, or in their early stages, often published as protocols. Studies involving Indigenous people were predominantly conducted in Australia and New Zealand, followed by the USA and Canada. There were only two reports exploring traditional direct preference elicitation methods (TTO or SG), while the majority of reports used indirect, multi-attribute PBM, the most common of which was the EQ-5D.

To our knowledge, this is the first systematic review to specifically explore the use of PBMs with Indigenous people, shedding light on their current application. Given the recent and relative increase in the number of studies reporting PBMs with Indigenous people, understanding performance metrics in this population is essential. Apart from a recent study of validity and reliability of the EQ-5D-5L for Indigenous Australians [52], studies evaluating performance are more than ten years old, presenting a possibly dated perspective of any of these instruments which have evolved to newer versions. Guidelines suggest first evaluating content and face validity of an instrument, followed by internal structure (construct validity), criterion validity, reliability and responsiveness [107]. Given this, it appears that for most colonized countries, a comprehensive evaluation of the performance of any PBM with Indigenous people should be done before using PBMs with these populations. COSMIN’s guidance further affirms the need to start with exploration of content validity and other forms of validity for any PBM that may be used to measure HRQL with Indigenous people [108].

There is a significant and growing body of Indigenous research and work published in academic journals that shares Indigenous perspectives on health, wellness, quality of life, and health related quality of life [2, 109–111]. This includes development of Aboriginal or Indigenous specific HRQL measures, such as the Aboriginal Children’s Health and Wellbeing Measure [112, 113]. It also includes accounts of what current PBMs of health-related quality of life (based on western biomedical models of health) fail to capture, such as elements of community, the environment, or spirituality [2, 29, 64, 110, 111, 114]. This review provides preliminary evidence of dimensions of health that may not be adequately captured for Maori people [64], as well as for Aboriginal and Torres Strait Islander people [103], emphasizing the need to integrate this knowledge into the exploration of validity and development of PBMs that are appropriate, accurate, valid, and reliable for Indigenous Peoples.

We did not find many reports related to the development or translation of PBMs to Indigenous contexts—Jelsma et al. [41] studied the performance of a Xhosa translation of the EQ-5D in South Africa, but the scarcity of translation exercises is noted. In communities in which traditional Indigenous language is still the primary language, translation of PBMs may be an important strategy to include traditional language speakers. Both Arrow et al. [10] and Howard et al. [104] proposed studies to develop a dental specific health utility scale and a preference-based wellbeing measure (respectively) in Australia. There are also recent efforts being made to generate an Indigenous specific PBM using Indigenous or decolonizing methodologies [64, 103, 104]. This approach should be modeled in the pursuit of similar questions around developing PBMs in other colonized countries. However, if Indigenous-specific HRQL instruments exist, perhaps there is an opportunity to investigate the appropriateness of deriving preferences for existing Indigenous-specific instruments.

The review by Angell et al. [8] focused on measures of HRQL with Indigenous people, but only captured one PBM. There are multiple factors that may have contributed to this recent increase. Firstly, there is a growing demand and interest in the application of PBMs in general, as they have become a common metric in evaluation of health and health system performance. Additionally, the global focus on reconciliation efforts prompted a shift in research practices to recognize, include, and partner with Indigenous people, aiming to address historic harms. The increasing number of studies in this review involving or reporting Indigenous people may be a result of this political and organizational shift towards engagement and reconciliation. In any case, we believe the inclusion of Indigenous people in studies using and reporting on PBMs is a beginning to the necessary representation and power shift in how research on PBMs is conducted or reported in Indigenous communities.

It is important to note that the majority of reports in this review did not clearly indicate whether the work was undertaken using currently accepted approaches for ethically engaged Indigenous research. Engaged approaches can reflect researchers' intentionality and their acknowledgment (or lack thereof) of the historical harms that colonizing research practices have inflicted upon Indigenous people. The lack of reporting likely calls for ongoing efforts to increase knowledge, awareness, and training in ethically engaged Indigenous research. It is also possible that academic journals publishing on PBMs are based in positivist epistemologies and are still maturing in their willingness and ability to include detailed information on engaged scholarship. Consequently, the records we found may not have provided comprehensive details regarding the methodology, particularly in terms of the extent of engagement with Indigenous communities.

PBMs, such as the EQ-5D, have already been adopted by some health systems to support health care resource allocation and economic evaluation [115]. The limited amount of recent information on performance of PBMs with Indigenous people suggests that the use of PBMs with Indigenous people should be applied with caution, and warrants consultation with Indigenous Peoples. There is recent, ethically engaged evidence of both validity (content, construct, criterion) and internal consistency of the EQ-5D-5L in Aboriginal and Torres Strait Islanders [52]. However, although this may indicate support for the EQ-5D-5L (at least in Aboriginal and Torres Strait Islanders), the authors also explicitly state that content validity was endorsed “in the absence of another suitable instrument being available” [52]. This aligns with concerns nearly two decades earlier about construct validity in Māori [9] that may suggest further exploration of fundamental questions of validity, and what constitutes health for Indigenous people. Decision-makers and policymakers may wish to exercise caution in their choice of PBM, and if they choose the EQ-5D, consider the transferability of Ribeiro’s findings to specific Indigenous communities in their region.

We undertook a very broad search strategy, in terms of populations, measures, study objectives and designs, intending to understand the full scope of use of PBMs with Indigenous populations. However, we were only able to include studies published in English. This review may also have failed to capture studies reporting broader measures of well-being, or studies supporting the early development of measures, such as theoretical or conceptual studies that may have preceded PBM-specific terminology. Due to the diversity of types of studies in this review, however, we chose not to evaluate the quality and strength of the included studies.

This work focused on describing the state of the peer-reviewed literature for PBMs with Indigenous people and explored how Indigenous groups and communities engaged in the work. This resource can be used to inform future work, and how research teams might engage with Indigenous partners in a research agenda on this topic. This review is based on the intention to understand how current PBMs are used and how they perform, such that they can be used, or put aside, appropriately.

This review suggests that further work is required to evaluate the performance of PBMs with Indigenous people. Given the predominant use of indirect PBMs, it is important to assess not only the validity of health status descriptive systems, but also the concept of valuation and preference elicitation. Given the limited evidence on the performance of current PBMs, future research might also focus on development of a PBM from an Indigenous-specific HRQL descriptive system and preference valuation that is culturally appropriate for Indigenous people. Work in this direction should only be pursued in an engaged manner, therefore relationship building between Indigenous communities, health economists and health policy actors would be beneficial. Lastly, exploration of the relevance of the Aboriginal and Torres Strait Islander quality assessment tool to other Indigenous contexts should be explored to support future systematic reviews and assessment of research.

Future research is not without ethical and political obligations for western researchers to recognize spaces for Indigenous self-determination in research, actively engaging in the work of decolonizing current (colonizing) research and policy systems [116, 117]. Similarly, theoretical assumptions of the health economic paradigm itself should be considered in terms of their euro-western roots and documented limitations [118], and the relation to Indigenous ways of knowing and Indigenous approaches to decision-making and priority-setting [119–121].

## Conclusion

PBMs have many applications, including assessment of population health and informing economic evaluation, and therefore resource allocation decisions, in many countries, including Canada. This review provides insight on the current breadth of use of PBMs worldwide with Indigenous people, as well as information on their validity and reliability. Understanding the validity and performance of PBMs with Indigenous populations is particularly important to better understand how such measures, or other measures, might be used in economic analysis that inform resource allocation decisions that affect Indigenous populations. Perhaps most importantly, it is our hope that this review can also facilitate meaningful conversations and work towards accurate and appropriate measurement of HRQL, particularly given the need to address health inequalities experienced by Indigenous people.

### Supplementary Information

Below is the link to the electronic supplementary material.

Supplementary file1 (DOCX 720 kb)

## Data Availability

Search strategies are available as an Online resource (Supplemental Information). This review was registered on PROSPERO (CRD42020205239). Please contact corresponding author for protocol, data collection forms or data extracted from included studies.

## References

[1] Drummond MF, Sculpher M, Claxton K, Stoddart GL, Torrance G (2015). Methods for the economic evaluation of health care programmes.

[2] Graham H, Stamler LL (2013). Contemporary perceptions of health from an Indigenous (Plains Cree) perspective. International Journal of Indigenous Health.

[3] Ellison-Loschmann L, Pearce N (2006). Improving access to health care among New Zealand’s Maori population. American Journal of Public Health.

[4] Gregory R, Easterling D, Kaechele N, Trousdale W (2016). Values-based measures of impacts to indigenous health. Risk Analysis Official Publications Society.

[5] Hansen KL (2015). Ethnic discrimination and health: the relationship between experienced ethnic discrimination and multiple health domains in Norway’s rural Sami population. International Journal of Circumpolar Health.

[6] Truth and Reconciliation Commission of Canada. Truth and Reconciliation Commission of Canada: Calls to action. (2015). Retrieved from http://nctr.ca/assets/reports/Calls_to_Action_English2.pdf

[7] MacDonald C, Steenbeek A (2015). The impact of colonization and Western assimilation on health and wellbeing of Canadian Aboriginal people. International Journal of Regional and Local History.

[8] Angell B, Muhunthan J, Eades A-M, Cunningham J, Garvey G, Cass A, Howard K, Ratcliffe J, Eades S, Jan S (2016). The health-related quality of life of Indigenous populations: A global systematic review. Quality of Life Research: An International Journal of Quality of Life Aspects of Treatment, Care and Rehabilitation.

[9] Perkins MRV, Devlin NJ, Hansen P (2004). The validity and reliability of EQ-5D health state valuations in a survey of Maori. Quality of Life Research.

[10] Arrow P, McPhee R, Atkinson D, Mackean T, Kularatna S, Tonmukayakul U, Brennan D, Palmer D, Nanda S, Jamieson L (2018). Minimally invasive dentistry based on atraumatic restorative treatment to manage early childhood caries in rural and remote Aboriginal communities: Protocol for a randomized controlled trial. JMIR Research Protocols.

[11] Banham D, Karnon J, Lynch J (2019). Health related quality of life (HRQoL) among Aboriginal South Australians: A perspective using survey-based health utility estimates. The Australian Indigenous HealthBulletin.

[12] Barnabe C, Crane L, White T, Hemmelgarn B, Kaplan GG, Martin L, Maksymowych WP (2018). Patient-reported outcomes, resource use, and social participation of patients with Rheumatoid Arthritis treated with biologics in Alberta: Experience of Indigenous and non-Indigenous patients. Journal of Rheumatology.

[13] Devlin N, Hansen P, Herbison P (2000). Variations in self-reported health status: Results from a New Zealand survey. New Zealand Medical Journal.

[14] Guevara SV, Feican EA, Pelaez I, Valdiviezo WA, Montaleza MA, Molina GM, Ortega NR, Delgado JA, Chimbo LE, Hernandez MV, Sanin LH, Cervera R (2020). Prevalence of rheumatic diseases and quality of life in the Saraguro Indigenous people, Ecuador: A cross-sectional community-based study. Journal of Clinical Rheumatology (JCR)..

[15] Lavergne MR, Kephart G (2012). Examining variations in health within rural Canada. Rural and Remote Health.

[16] Ramirez-Cervantes KL, Remes-Troche JM, Del Pilar Milke-Garcia M, Romero V, Uscanga LF (2015). Characteristics and factors related to quality of life in Mexican Mestizo patients with celiac disease. BMC Gastroenterology.

[17] Goodwin E, Green C (2016). A systematic review of the literature on the development of condition-specific preference-based measures of health. Applied Health Economics and Health Policy.

[18] Campbell, S. (2021). A *filter to retrieve studies related to Indigenous people of Australia and the Torres Strait Isalnd from the Ovid MEDLINE Database*. John W. Scott Health Sciences Library, University of Alberta. Retrieved from https://docs.google.com/document/d/15g260L_hRKgYCh-iygS_QHI1cvGlSsphd5XwlrSk2Tg/edit#

[19] Campbell, S. (2021). *Filter to retrieve studies related to Indigenous people of the United States from the OVID Medline Database*. John W. Scott Health Sciences Library, University of Alberta. Retrieved from https://docs.google.com/document/d/118tP1FvgQ1hRROjI1QroLjM-u8WN_uS6Nafis6s37jk/edit

[20] Campbell S (2020). A filter to retrieve studies related to Sami people from the Ovid MEDLINE Database.

[21] Campbell, S., Dorgan, M., & Tjosvold, L. (2016). *Filter to retrieve studies related to Indigenous people of Canada the OVID Medline Database*. John W. Scott Health Sciences Library, University of Alberta, 2016. Retrieved from https://docs.google.com/document/d/1XqpWHN7hrFIyNwaqucRFRXaCnBOaeshFw4SR31Uxyek/edit

[22] United Nations. (2006). *Fifth Session Fact Sheet 1: Indigenous peoples and identity. United Nations Permanent Forum on Indigenous Issues*. Retrieved from November 15, 2022, from https://www.un.org/development/desa/indigenouspeoples/unpfii-sessions-2/fifth-session-of-unpfii.html

[23] United Nations. (2022). *United Nations for Indigenous peoples*. Indigenous Peoples at the United Nations. Retrieved from November 15, 2022, from https://www.un.org/development/desa/indigenouspeoples/about-us.html

[24] Government of Canada, Crown-Indigenous Relations and Northern Affairs Canada. (2009). *Indigenous peoples and communities*. Indigenous Peoples and Communities. Retrieved from November 15, 2022, from https://www.rcaanc-cirnac.gc.ca/eng/1100100013785/1529102490303

[25] First Nations Information Governance Center. (2022). *The First Nations Principles of OCAP*. The First Nations Information Governance Centre. Retrieved from June 06, 2002, from https://fnigc.ca/ocap-training/

[26] Australian Institute for Aboriginal and Torres Strait Islander Studies. (2012). *Guidelines for ethical research in Australian Indigenous Studies*. Australian Institute of Aboriginal and Torres Strait Islander Studies. Retrieved from https://aiatsis.gov.au/sites/default/files/2020-09/gerais.pdf

[27] Inuit Tapiriit Kanatami and Nunavut Research Institute. (2006). *Negotiating research relationships with Inuit communities: A guide for researchers*. Inuit Tapiriit Kanatami and Nunavut Research Institute.

[28] Canadian Institutes of Health Research. (2027). Government of Canada. Defining Indigenous health research—CIHR. Defining Indigenous Health Research. Retrieved from November 15, 2002, from https://cihr-irsc.gc.ca/e/50340.html

[29] Harfield S, Pearson O, Morey K, Kite E, Canuto K, Glover K, Gomersall JS, Carter D, Davy C, Aromataris E, Braunack-Mayer A (2020). Assessing the quality of health research from an Indigenous perspective: The Aboriginal and Torres Strait Islander quality appraisal tool. BMC Medical Research Methodology.

[30] Chenhall R, Senior K (2012). Treating Indigenous Australians with alcohol/drug problems: Assessing quality of life. Alcoholism Treatment Quarterly.

[31] Derrett S, Davie G, Ameratunga S, Wyeth E, Colhoun S, Wilson S, Samaranayaka A, Lilley R, Hokowhitu B, Hansen P, Langley J (2011). Prospective outcomes of injury study: Recruitment, and participant characteristics, health and disability status. Injury Prevention.

[32] Derrett S, Harcombe H, Wyeth E, Davie G, Samaranayaka A, Hansen P, Hall G, Cameron ID, Gabbe B, Powell D, Sullivan T, Wilson S, Barson D (2017). Subsequent Injury Study (SInS): Improving outcomes for injured New Zealanders. Injury Prevention Journal of the International Society for Child and Adolescent Injury Prevention.

[33] Devlin N, Hanson P, Herbison P (2000). Variations in self-reported health status: Results from a New Zealand survey. New Zealand Medical Journal.

[34] Dingwall KM, Sweet M, Cass A, Hughes JT, Kavanagh D, Howard K, Barzi F, Brown S, Sajiv C, Majoni SW, Nagel T (2021). Effectiveness of Wellbeing Intervention for Chronic Kidney Disease (WICKD): Results of a randomised controlled trial. BMC Nephrology.

[35] du Toit GC, Kidd M (2016). An analysis of the psychometric properties of the translated versions of the European Organisation for the Research and Treatment of Cancer QLQ CX24 questionnaire in the two South African indigenous languages of Xhosa and Afrikaans. European Journal of Cancer Care (England).

[36] Garvey G, Cunningham J, He VY, Janda M, Baade P, Sabesan S, Martin JH, Fay M, Adams J, Kondalsamy-Chennakesavan S, Valery PC (2016). Health-related quality of life among Indigenous Australians diagnosed with cancer. Quality of Life Research.

[37] Groessl EJ, Kaplan RM, Cronan TA (2003). Quality of well-being scale. Arthritis & Rheumatology.

[38] Hay JW, Gong CL, Jiao X, Zawadzki NK, Zawadzki RS, Pickard AS, Xie F, Crawford SA, Gu NY (2021). A US Population Health Survey on the impact of COVID-19 Using the EQ-5D-5L. Journal of General Internal Medicine.

[39] Hughes J, Jelsma J, Maclean E, Darder M, Tinise X (2004). The health-related quality of life of people living with HIV/AIDS. Disability and Rehabilitation.

[40] Janda M, DiSipio T, Hurst C, Cella D, Newman B (2009). The Queensland Cancer Risk Study: General population norms for the Functional Assessment of Cancer Therapy-General (FACT-G). Psycho-Oncology.

[41] Jelsma J, Mkoka S, Amosun L, Nieuwveldt J (2004). The reliability and validity of the Xhosa version of the EQ-5D. Disability and Rehabilitation.

[42] Johnson DR, McDermott RA, Clifton PM, D'Onise K, Taylor SM, Preece CL, Schmidt BA (2015). Characteristics of Indigenous adults with poorly controlled diabetes in north Queensland: Implications for services. BMC Public Health.

[43] Ju X, Hedges J, Garvey G, Smith M, Canfell K, Jamieson L (2021). Poor self-rated oral health associated with poorer general health among Indigenous Australians. BMC Public Health.

[44] Kularatna S, Lalloo R, Kroon J, Tadakamadla SKK, Scuffham PA, Johnson NW (2020). Demonstration of high value care to improve oral health of a remote Indigenous community in Australia. Health and Quality of Life Outcomes.

[45] Lapsley H, Hayman KJ, Muru-Lanning ML, Moyes SA, Keeling S, Edlin R, Kerse N (2020). Caregiving, ethnicity and gender in Maori and non-Maori New Zealanders of advanced age: Findings from LiLACS NZ kaiawhina (love and support) study. Australasian Journal on Ageing.

[46] Mann J, Thompson F, Devine S, Quigley R, Strivens E, McDermott R (2021). Beyond multimorbidity: Primary care and the older person with complex needs. Australian Journal of Primary Health.

[47] McClure TM, Choi D, Wooten K, Nield C, Becker TM, Mansberger SL (2011). The impact of eyeglasses on vision-related quality of life in American Indian/Alaska Natives. American Journal of Ophthalmology.

[48] McDermott R, Schmidt B, Preece C, Owens V, Taylor S, Li M, Esterman A (2015). Community health workers improve diabetes care in remote Australian Indigenous communities: results of a pragmatic cluster randomized controlled trial. BMC Health Services Research.

[49] McNoe B, Schollum JBW, Derrett S, Marshall MR, Henderson A, Samaranayaka A, Walker RJ (2019). Recruitment and participant baseline characteristics in the dialysis outcomes in those aged 65 years or older study. BMC Nephrology.

[50] Mold JW, Vesely SK, Keyl BA, Schenk JB, Roberts M (2004). Quality of Well-Being–Self-Administered Questionnaire. Journal of the American Board of Family Medicine.

[51] Oen, K., Guzman, J., Dufault, B., Tucker, L. B., Shiff, N. J., Duffy, K. W., Lee, J. J. Y., Feldman, B. M., Berard, R. A., Dancey, P., Huber, A. M., Scuccimarri, R., Cabral, D. A., Morishita, K. A., Ramsey, S. E., Rosenberg, A. M., Boire, G., Benseler, S. M., Lang, B., … Duffy, C. M. (2018). Health-related quality of life in an inception cohort of children with Juvenile Idiopathic Arthritis: A longitudinal analysis. *Arthritis Care Research,**70*(1), 134–144. 10.1002/acr.23236 28320056

[52] Ribeiro Santiago PH, Haag D, Macedo DM, Garvey G, Smith M, Canfell K, Hedges J, Jamieson L (2021). Psychometric properties of the EQ-5D-5L for aboriginal Australians: a multi-method study. Health Quality of Life Outcomes.

[53] Sato A (2012). Revealing the popularity of traditional medicine in light of multiple recourses and outcome measurements from a user’s perspective in Ghana. Health Policy and Planning.

[54] Segal L, Nguyen H, Schmidt B, Wenitong M, McDermott RA (2016). Economic evaluation of Indigenous health worker management of poorly controlled type 2 diabetes in north Queensland. Medical Journal of Australia.

[55] Walker N, Smith B, Barnes J, Verbiest M, Parag V, Pokhrel S, Wharakura M, Lees T, Gutierrez HC, Jones B, Bullen C (2021). Cytisine versus varenicline for smoking cessation in New Zealand indigenous Maori: A randomized controlled trial. Addiction (Abingdon, England).

[56] Wilson C, Huang C, Shara N, Howard BV, Fleg JL, Henderson JA, Howard WJ, Huentelman H, Lee ET, Mete M, Russell M, Galloway JM, Silverman A, Sylianou M, Umans J, Weir MR, Yeh F, Ratner RE (2010). Cost-effectiveness of lower targets for blood pressure and low-density lipoprotein cholesterol in diabetes: The Stop Atherosclerosis in Native Diabetics Study (SANDS). Journal of Clinical Lipidology.

[57] Donaldson LH, Hammond NE, Agarwal S, Taylor S, Bompoint S, Coombes J, Bennett-Brook K, Bellomo R, Myburgh J, Venkatesh B (2022). Outcomes following severe septic shock in a cohort of Aboriginal and Torres Strait Islander people: a nested cohort study from the ADRENAL trial. Critical Care and Resuscitation.

[58] Alfonso YN, Bishai D, Ivanich JD, O'Keefe VM, Usher J, Aldridge LR, Haroz EE, Goklish N, Barlow A, Cwik M (2022). Suicide ideation and depression quality of life ratings in a reservation-based community of native American youths and young adults. Community Mental Health Journal.

[59] Gall A, Diaz A, Garvey G, Anderson K, Lindsay D, Howard K (2022). Self-reported wellbeing and health-related quality of life of Aboriginal and Torres Strait Islander people pre and post the first wave of the COVID-19 2020 pandemic. Australian and New Zealand Journal of Public Health.

[60] LaGrappe D, Massey L, Kruavit A, Howarth T, Lalara G, Daniels B, Wunungmurra JG, Flavell K, Barker R, Flavell H, Heraganahally SS (2022). Sleep disorders among Aboriginal Australians with Machado-Joseph Disease: Quantitative results from a multiple methods study to assess the experience of people living with the disease and their caregivers. Neurobiology of Sleep and Circadian Rhythms.

[61] Maclennan B, Wyeth E, Samaranayaka A, Derrett S (2022). Predictors of EQ-5D-3L outcomes amongst injured Maori: 1-year post-injury findings from a New Zealand cohort study. Quality of Life Research: An International Journal of Quality of Life Aspects of Treatment, Care and Rehabilitation.

[62] Shettigar R, Samaranayaka A, Schollum JBW, Wyeth EH, Derrett S, Walker RJ (2021). Predictors of health deterioration among older New Zealanders undergoing dialysis: A three-year accelerated longitudinal cohort study. Canadian Journal of Kidney Health and Disease.

[63] Thompson SG, Barber PA, Gommans JH, Cadihac DA, Davis A, Fink JN, Harwood M, Levack W, McNaughton H, Feigin VL, Girvan J, Denison H, Corbin M, Wilson A, Douwes J, Ranta A (2022). The impact of ethnicity on stroke care access and patient outcomes: a New Zealand nationwide observational study. The Lancet Regional Health – Western Pacific.

[64] Willing E, Paine S-J, Wyeth E, Te Ao B, Vaithianathan R, Reid P (2020). Indigenous voices on measuring and valuing health states. AlterNative.

[65] Ju X, Canfell K, Howard K, Garvey G, Hedges J, Smith M, Jamieson L (2021). Population-based utility scores for HPV infection and cervical squamous cell carcinoma among Australian Indigenous women. PLOS ONE.

[66] Ju X, Hedges J, Garvey G, Smith M, Canfell K, Jamieson L (2021). Population-based utility scores for HPV infection and oropharyngeal squamous cell carcinoma among Indigenous Australians. BMC Public Health.

[67] Elder-Robinson E, Diaz A, Howard K, Parikh DR, Kar G, Garvey G (2021). Quality of life in the first year of cancer diagnosis among Aboriginal and non-Aboriginal people living in regional and remote areas of Australia. International Journal of Environmental Research and Public Health.

[68] Nagel T, Sweet M, Dingwall KM, Puszka S, Hughes JT, Kavanagh DJ, Cass A, Howard K, Majoini SW (2020). Adapting wellbeing research tools for Aboriginal and Torres Strait Islander people with chronic kidney disease. BMC Nephrology.

[69] Ralph-Campbell K, Pohar SL, Guirguis LM, Toth EL (2006). Aboriginal participation in the DOVE study. Canadian Journal of Public Health = Revue Canadienne De Santé Publique.

[70] Cochrane Library. (2019). Dementia prevention and risk Management Program for Aboriginal Australians—DAMPAA Project. Cochrane Central Register of Controlled Trials. Retrieved from https://www.cochranelibrary.com/central/doi/10.1002/central/CN-01975175/full

[71] Lott, N. (2019). A randomised controlled trial to evaluate the effects of shared decision making within a multidisciplinary team on decision making in the older adult population considering surgery. World Health Organization International Clinical Trials Registry Platform. Retrieved from October 17, 2021, from https://www.cochranelibrary.com/central/doi/10.1002/central/CN-02064935/full

[72] Toombs, M. (2018). Indigenous Model of Mental Health Care. World Health Organization International Clinical Trials Registry Platform. Retrieved from October 17, 2021, from https://www.cochranelibrary.com/central/doi/10.1002/central/CN-01948784/full

[73] Ingham, T. (2017). Whakapai e Te Ara Ha: Asthma self-management programme for the whanau of Tamariki Maori with asthma. Australian New Zealand Clinical Trials Registry. https://www.cochranelibrary.com/central/doi/10.1002/central/CN-01889867/full

[74] Radford, K. (2019). Standing Tall with Our Mob Program (STOMP) pilot trial to improve mobility, balance, physical activity, cognitive function and psychological well-being with older people in an urban Aboriginal community. International Clinical Trials Registry Platform Search Portal. Retrieved from November 15, 2022, from https://trialsearch.who.int/Trial2.aspx?TrialID=ACTRN12619001130156

[75] Delbaere, K., & Veinovic, M. (2021). StandingTall with our Mob Project (STOMP!): A holistic approach towards active and healthy ageing of Aboriginal and Torres Strait Islander peoples. Australian New Zealand Clinical Trials Registry. Retrieved from May 11, 2023, from https://anzctr.org.au/Trial/Registration/TrialReview.aspx?id=382062&showOriginal=true&isReview=true

[76] Hachem, M. (2021). Can Flash Glucose Monitoring (FlashGM) improve glucose management in Indigenous Australians with type 2 diabetes? International Clinical Trials Registry Platform Search Portal. Retrieved from May 10, 2023, from https://trialsearch.who.int/Trial2.aspx?TrialID=ACTRN12621000753853

[77] Jamieson, L. M. (2022). Investigating the effect of a silver fluoride intervention on the life journeys of young Indigenous peoples and the arresting of dental caries across the life course. International Clinical Trials Registry Platform Search Portal. Retrieved from April 25, 2023, from https://trialsearch.who.int/Trial2.aspx?TrialID=ACTRN12622001066774

[78] Harcombe, H., Derrett, S., Wyeth, E., Maclennan, B., & Barson, D. (2022). Effect of a telephone follow-up intervention on subsequent injuries: A feasibility study. Australian New Zealand Clinical Trials Registry. Retrieved from May 11, 2023, from https://www.anzctr.org.au/Trial/Registration/TrialReview.aspx?id=384053&isReview=true

[79] Taylor W (2005). Musculoskeletal pain in the adult New Zealand population: Prevalence and impact. New Zealand Medical Journal.

[80] Altomare I, Colucci P, Parasuraman S, Paranagama DC, Al-Janadi A (2018). Disease characteristics of minority patient populations with polycythemia vera: An analysis from the reveal study. Blood.

[81] Barnabe C, Hemmelgarn B, Kaplan G, Martin L, Maksymowych W (2015). Treatment outcomes with biologic therapies for rheumatoid arthritis in the alberta aboriginal population. Journal of Rheumatology.

[82] Custer B, Vahidnier F, Kesser D, Lepare G, Krystof D, Shaz B, Stramer S (2014). Health-related quality of life in us blood donors with and without viral infections. Vox Sang..

[83] Dingwall KM, Hughes JT, Sweet M, Cass A, Kavanagh D, Howard K, Barzi F, Brown S, Sajiv C, Majoni SW, Nagel T (2020). Wellbeing intervention for chronic kidney disease: The wickd trial. Nephrology.

[84] Farace E, Sheehan J (2014). Trajectory of quality of life at end of life in malignant glioma: Support for the terminal drop theory. Neuro-Oncology.

[85] Garvey G, Beesley VL, Janda M, O’Rourke P, He VYF, Hawkes AL, Elston J, Green AC, Cunningham J, Valery PC (2014). Psychometric properties of an Australian supportive care needs assessment tool for indigenous people (SCNAT-IP) with cancer. Asia – Pacific Journal of Clinical Oncology.

[86] Kilkenny M, Lannin N, Kim J, Thrift A, Donnan G, Hill K, Grimley R, Middleton S, Anderson C, Cadilhac D (2018). Stroke care and outcomes for Australian Aboriginal and non-Aboriginal patients: Observational study from the Australian Stroke Clinical Registry. International Journal of Stroke.

[87] Moodie M, Keating C, Mavoa H, Fotu K, Waqa G, Faeamani G, Swinburn B (2010). The impact of obesity on the quality of life of adolescents in different ethnic groups. Obesity Reviews.

[88] Morales-Arango F, Moctezuma JF, Loyola-Sanchez A, Garcia H, Alvarez-Hernandez E, Vazquez-Mellado J, Ayora-Manzano H, Cruz-Martin G, Flores-Aguilar D, Pereira-Zaldivar R, Zarate-Dominguez M, Mendoza M, Pelaez-Ballestas I (2018). High prevalence of seronegative rheumatoid arthritisin a maya-yucateco indigenous population: A cohort community-based study. Annals of Rheumatic Diseases.

[89] Lavergne, M. R. (2009). *Health in non-metropolitan Canada: Beyond the urban/rural dichotomy*. Dalhousie University. Retrieved from https://login.ezproxy.library.ualberta.ca/login?url=https://www.proquest.com/docview/305073000?accountid=14474

[90] Derrett S, Langley J, Hokowhitu B, Ameratunga S, Hansen P, Davie G, Wyeth E, Lilley R (2009). Prospective outcomes of injury study. Injury Prevention Journal of the International Society for Child and Adolescent Injury Prevention.

[91] Dingwall KM, Nagel T, Hughes JT, Kavanagh DJ, Cass A, Howard K, Sweet M, Brown S, Sajiv C, Majoni SW (2019). Wellbeing intervention for chronic kidney disease (WICKD): A randomised controlled trial study protocol. BMC Psychology.

[92] Kinchin I, Jacups S, Mann J, Quigley R, Harvey D, Doran CM, Strivens E (2018). Efficacy and cost-effectiveness of a community based model of care for older patients with complex needs: A study protocol for a multicentre randomized controlled trial using a stepped wedge cluster design. Trials.

[93] Lalloo R, Krooen J, Tut O, Kularatna S, Jamieson LM, Wallace V, Boase R, Fernando S, Cadet-James Y, Scuffham PA, Johnson NW (2015). Effectiveness, cost-effectiveness and cost-benefit of a single annual professional intervention for the prevention of childhood dental caries in a remote rural Indigenous community. BMC Oral Health.

[94] Liu H, Patel A, Brown A, Eades S, Hayman N, Jan S, Ring I, Stewart G, Tonkin A, Weeramanthri T, Wade V, Rodgers A, Usherwood T, Neal B, Peiris D, Burke H, Reid C, Cass A (2010). Rationale and design of the Kanyini guidelines adherence with the polypill (Kanyini-GAP) study: A randomised controlled trial of a polypill-based strategy amongst indigenous and non indigenous people at high cardiovascular risk. BMC Public Health.

[95] Walker N, Smith B, Barnes J, Verviest M, Kurdziel T, Parag V, Pokhrel S, Bullen C (2019). Cytisine versus varenicline for smoking cessation for Maori (the indigenous people of New Zealand) and their extended family: Protocol for a randomized non-inferiority trial. Addiction (Abingdon, England).

[96] Ranta A, Thompson S, Harwood MLN, Cadilhac DA, Barber PA, Davis A, Gommans JH, Fink JN, McNaughton HK, Denison H, Corbin M, Feigin V, Abernethy V, Levack W, Douwes J, Girvan J, Wilson A (2021). Reducing ethnic and geographic inequities to optimise New Zealand Stroke Care (REGIONS Care): Protocol for a Nationwide Observational Study. JMIR Research Protocols.

[97] Hatcher S, Coupe N, Duri M, Elder H, Tapsell R, Wikiriwhi K, Parag V (2011). Te Ira Tangata: A Zelen randomised controlled trial of a treatment package including problem solving therapy compared to treatment as usual in Maori who present to hospital after self harm. Trials.

[98] Derrett S, Beaver C, Sullivan MJ, Herbison GP, Acland R, Paul C (2012). Traumatic and non-traumatic spinal cord impairment in New Zealand: Incidence and characteristics of people admitted to spinal units. Injury Prevention Journal of the International Society for Child and Adolescent Injury Prevention.

[99] Scholes-Robertson N, Blazek K, Tong A, Craig J, Essue B, Howard K, Howell M (2021). The financial burden of Chronic Kidney Disease for rural Australian families. Nephrology.

[100] Armstrong E, Coffin J, Hersh D, Katzenellenbogen JM, Thompson S, Flicker L, McAllister M, Cadilhac DA, Rai T, Godecke E, Hayward C, Hankey G, Drew N, Lin I, Woods D, Ciccone N (2021). Healing Right Way: study protocol for a stepped wedge cluster randomised controlled trial to enhance rehabilitation services and improve quality of life in Aboriginal Australians after brain injury. BMJ Open.

[101] Kinchin I, Doran CM, McCalman J, Jacups S, Tsey K, Lines K, Smith K, Searles A (2017). Delivering an empowerment intervention to a remote Indigenous child safety workforce: Its economic cost from an agency perspective. Evaluation and Program Planning.

[102] Cousins K, Norris P, Horsburgh S, Smith A, Keown S, Samaranayaka A, Marra C, Churchward M (2021). Impact of removing prescription charges on health outcomes: protocol for a randomised controlled trial. BMJ Open.

[103] Anderson K, Howard K, Cunningham J, Butler TL, Gall A, Arley B, Garvey G (2021). What matters: Development of a well-being measure for Aboriginal and Torres Strait Islander adults. Asia-Pacific Journal of Clinical Oncology.

[104] Howard K, Anderson K, Cunningham J, Cass A, Ratcliffe J, Whop LJ, Dickson M, Viney R, Mulhern B, Tong A, Garvey G (2020). What Matters 2 Adults: A study protocol to develop a new preference-based wellbeing measure with Aboriginal and Torres Strait Islander adults (WM2Adults). BMC Public Health.

[105] Nagel T, Sweet M, Dingwall KM, Puszka S, Hughes JT, Kavanagh DJ, Cass A, Howard K, Majoni SW (2020). Adapting wellbeing research tools for Aboriginal and Torres Strait Islander people with chronic kidney disease. BMC Nephrology.

[106] Parkin D, Devlin N (2006). Is there a case for using visual analogue scale valuations in cost-utility analysis?. Health Economics.

[107] Prinsen CAC, Mokkink LB, Bouter LM, Alonso J, Patrick DL, de Vet HCW, Terwee CB (2018). COSMIN guideline for systematic reviews of patient-reported outcome measures. Quality of Life Research.

[108] Mokkink LB, Terwee CB, Patrick DL, Alonso J, Stratford PW, Knol DL, Bouter LM, de Vet HCW (2010). The COSMIN checklist for assessing the methodological quality of studies on measurement properties of health status measurement instruments: An international Delphi study. Quality of Life Research.

[109] Fiedeldey-Van Dijk C, Rowan M, Dell C, Mushquash C, Hopkins C, Fornssler B, Hall L, Mykota D, Farag M, Shea B (2017). Honoring Indigenous culture-as-intervention: Development and validity of the Native Wellness Assessment. Journal of Ethnicity in Substance Abuse.

[110] Richmond CA, Ross NA, Bernier J, White J, Beavon D, Wingert S, Maxim P (2007). Exploring indigenous concepts of health: the dimensions of Métis and Inuit health. Aboriginal policy research: Directions and outcomes.

[111] Smith K, Gilchrist L, Taylor K, Clinch C, LoGiudice D, Edgill P, Ratcliffe J, Flicker L, Douglas H, Bradely K, Bessarab D (2020). Good Spirit, good life: A quality of life tool and framework for older Aboriginal peoples. The Gerontologist.

[112] Young NL, Wabano MJ, Ritchie SD, Burke TA, Pangowish B, Corbiere RG (2015). Assessing children’s interpretations of the Aboriginal Children’s Health and Well-Being Measure (ACHWM). Health and Quality of Life Outcomes.

[113] Young NL, Wabano MJ, Blight S, Baker-Anderson K, Beaudin R, McGregor LF, McGregor LE, Burke TA (2017). Relevance of the aboriginal children’s health and well-being measure beyond Wiikwemkoong. Rural Remote Health.

[114] Colomeda LA, Wenzel ER (2000). Medicine keepers: Issues in indigenous health. Critical Public Health.

[115] Canadian Agency for Drugs and Technologies in Health (2017). Guidelines for the economic evaluation of health technologies: Canada.

[116] Wilson NJ (2019). ‘Seeing Water Like a State?’: Indigenous water governance through Yukon First Nation Self-Government Agreements. Geoforum.

[117] Green R (2015). The economics of reconciliation: Tracing investment in Indigenous–settler relations. Journal of Genocide Research.

[118] Mooney G (2009). Challenging health economics.

[119] Angell, B. J. (2017). *Health economics and Indigenous health: Measuring value beyond health outcomes*. Unpublished Doctor of Philosophy Thesis.

[120] Otim, M., Asante, K., Kelaher, M., Doran, C., & Anderson, I. (2015). What constitutes benefit from health care interventions for Indigenous Australians? *Australian Aboriginal Studies* (1), 30–42.

[121] Adamowicz W, Beckley T, MacDonald DH, Just L, Luckert M, Murray E, Phillips W (1998). In search of forest resource values of indigenous peoples: Are nonmarket valuation techniques applicable?. Society and Natural Resources.

[122] Haddaway, N. R., Page, M. J., Pritchard, C. C., & McGuinness, L. A. (2022). PRISMA2020: An R package and Shiny app for producing PRISMA 2020-compliant flow diagrams, with interactivity for optimised digital transparency and Open Synthesis Campbell Systematic Reviews, 18, e1230. 10.1002/cl2.1230 PMC8958186 36911350

