# Supplementary material for: Preference-based measures of health-related quality of life in Indigenous people: a systematic review
Source: Qual Life Res. 2023 Sep 16;33(2):317–33. doi: 10.1007/s11136-023-03499-7 (PMC10850204; doi:10.1007/s11136-023-03499-7)
Supplement: Supplementary file 1 — Supplementary file1 (DOCX 720 kb) [file 11136_2023_3499_MOESM1_ESM.docx]

**Supplementary Information**

**Article Title:** Preference-based measures of health-related quality of life in Indigenous People: A systematic review

**Journal Name:** Quality of Life Research

**Author Names:** Lilla M. Roy, Aidan Neill, Kristen Swampy, Elder Juliette Auger, Sandra M. Campbell, Susan Chatwood, Fatima Al Sayah and Jeffrey A. Johnson

**Corresponding Author:** Jeffrey Johnson, jeff.johnson@ualberta.ca

**Supplementary Information Content**:

- Search Strategies (p. 2)
- Description of Author Position (p. 39)
- Table I Characteristics of Included Reports (in full) (p. 40)
- Table II Performance of PBMs with Indigenous People (p. 46)

**Search Strategies**

**Ovid MEDLINE(R) ALL <1946 to August 30, 2022>**

| **#** | **Search Statement** | **Results** |
| --- | --- | --- |
| 1 | american native continental ancestry group/ or alaska natives/ or indians, north american/ or inuits/ or indigenous peoples/ or (first nation or first nations or first people* or original people* or "first inhabitant*" or original inhabitant* or autochtone or aborigin* or indigenous or metis or inuit or innu or inuk or inuvialuit or american indian* or native american*).mp. | 73250 |
| 2 | ((Indigenous People/ or American Indian/ or First Nation/ or Metis/ or Eskimo/ or Inuit/ or Indigenous Health Services/ or exp Ethnopharmacology/ or (Algonquin or Anishinaabe or Assiniboine or Athapaskan or Blackfoot or Cayuga or Chippewa or Cree or Dakelh or Dene or Dunne-za or Gitsxan or Gwich'in or Haida or (Han not (Chinese or China or Tibet*)) or Haisla or Heiltsuk or Haudenosaunee or Kwakwaka'wakw or Inuit or Inuk or ((Iroquios or Iroquoian) not (homeobox or corn)) or Kaska or Ktunaxa or Nlaka'pamux or Nakoda or Nakota or Nuxalk or Maliseet or Meits or Mi'kmaq or Micmac or Mohawk or Nipissing or Nisga'a or Nuu chah nulth or jibwa or Ojibwe or Ojibway or Oji-Cree or Okanagan or Oneida or Onodaga or Oweenkeno or Passamaquoddy or Potawatomi or Salish or Saulteaux or Sec wepmc or Sekani or Seneca or Stl'atlimc or Tagish or Tasttine or Tahltan or Tlingit or Tsimshian or Tsilhqot'in or Tsuu T'inia or Tuscarora or Tutchone or Wakashan or Wyandot).mp. or Aboriginal*.mp. or Indigenous*.mp. or red [road.mp](http://road.mp/). or "on reserve".mp. or [off-reserve.mp](http://off-reserve.mp/). or First Nation.mp. or First Nations.mp. or Amerindian.mp. or (urban adj3 (Indian* or Native* or Aboriginal*)).mp. or [ethnomedicine.mp](http://ethnomedicine.mp/). or country food*.mp. or residential school*.mp. or ((exp Medicine, Traditional/ or traditional medicine*.mp.) not Chinese.mp.) or exp Shamanism/ or shaman*.mp. or traditional heal*.mp. or traditional food*.mp. or medicine [man.mp](http://man.mp/). or medicine [woman.mp](http://woman.mp/). or autochtone*.mp. or (Native* adj1 (american or man or men or women or woman or boy* or girl* or adolescent* or youth or youths or person* or adult or people* or Indian* or Nation or tribe* or tribal or band or bands)).mp.) and (exp Canada/ or (Canad* or British Columbia or Columbie Britannique or Alberta or Saskatchewan or Manitoba or Ontario or Quebec or Nova Scotia or New Brunswick or Newfoundland or Labrador or Prince Edward Island or Yukon Territory or NWT or Northwest Territories or Nunavut).mp.)) or (Canadian Aboriginal/ not oriental medicine/) or (Innu or Inuvialuit* or Nunavik or Nunatsiavut or NunatKavut or Wet'suwet'en).mp. [mp=title, book title, abstract, original title, name of substance word, subject heading word, floating sub-heading word, keyword heading word, organism supplementary concept word, protocol supplementary concept word, rare disease supplementary concept word, unique identifier, synonyms] | 7851 |
| 3 | (Saami or Sampi or (Sami not Ulus) or Samis or Southernsami* or Umesami* or Pitesami* or Lulesami* or Northernsami* or Enaresami* or Kolasami* or Lapp or Lapps or Lappish or Lappland or (Lapland* not longspur) or Lappalainen* or Saamelainen* or reindeer herd* or reindeer culture* or reindeer pastoral* or Lappbys or Samebys or reinbeitesdistrikt or paliskunta or siida).mp. or (((Fennoscandia or Finnmark or Scandinavia or Nordic or Sweden or Norway or Finland or Swedish or Finnish or Norwegian or Norge or Svensk* or Suomi or Barents Region or (Kola not (garcinia or gotu)) or Arctic Europe* or Polar Europe* or North* Europ*).mp. or Finland/ or Norway/ or Sweden/) and ((traditional adj2 (food* or heal* or medicine* or shaman*)) or (Indigen* adj3 (people* or person* or mother* or father* or parent* or child* or boy or boys or girl* or youth* or healer* or famil* or herder*))).mp.) | 1519 |
| 4 | (((Indians, North American/ or Inuits/) and (Alaska/ or Alaska*.mp.)) or (((Anvik or Barrow or Bethel or Buckland or Cantwell or Chilcoot or Clarkes Point or Cordova or Craig or Crooked Creek or Deering or Dot Lake or Douglas or Eagle or Eek Elim or English Bay or Evansville or False Pass or Galena or Louden or Gambel or Georgetown or (Haida not (Haida Gwaii or Skidegate or Charlotte or Canada)) or Haines or Hamilton or Healy Lake or Holy Cross or Hughes or Kake or King Island or King Salmon or Kotlik or Kwethluik or Marshall or McGrath or Minto or Nome or Northway or Old Harbor or Oscarville or Sheldon's Point or Perryville or (Petersburg not St Petersburg) or Pilot Point or Pilot Station or Point Hope or Point Lay or Port Graham or Port Heiden or Port Lions or Pribilof* or St Paul Island or St George Island* or Sand Point or Rampart or Red Devil or Ruby or Saxman or Saint Michael or Stebbins or Stevens or Stony River or twin Hills) adj3 (Eskimo or Indian or community or Native or traditional or Indigenous or tribe or tribes or tribal or elder or elders or people or peoples)) or ((Native Village and Alaska*) or ((Native adj2 Alaska*) not species) or "Indians/alaska" or Afognak or Agdaagux or Akhiok or Akiachak or Akiak or Akutan or Alakanuk or Alatna or Aleknagik or Algaaciq or Allakaket or Anaktuvuk or Andreafski or Angoon Community or Aniak or Arctic Village or Venetie or Asa'carsarmiut or Atka or Atmautluak or Atqasuk or Atkasook or "beaver village" or "Bill Moore* Slough" or Belkofski or Birch Creek Tribe or Brevig Mission or Chenega or Chanega or Chalkyitsik or Cheesh-Na Chistochina or Chefornak or Chevak or Chickaloon or Chignik or Chilkat or Copper Center Village or Klukwan or Chinik or Golovin or Chitina or Chuathbaluk or Kuskokwim or Chuloonawick or Curyung or Diomede or Inalik or Egegik or Eklutna or Ekuk or Ekwok or Emmonak or Bettles Field or Fort Yukon or Gakona or Goodnews Bay or Holikachuk or Gulkana or Hoonah or Hooper Bay or Huslia or Hydaburg or Igiugig or Iliamna or Inupiat or Inupiaq or Arctic Slope or Iqurmuit or Ivanoff Bay or Kaguyak or Kaktovik or Barter Island or Kalskag or Kaltag or Kanatak or Karluk or Kasaan or Kasigluk or Elders Council or Kasigluk or Kenaitze or Ketchikan or Kiana or Kipnuk or Kivalina or Klawock or Kluti Kaah or Knik Tribe or Kobuk or Kokhanok or Kongiganak or Kotlik or Kotzebue or Koyuk or Koyukuk or Kwethluk or Kwigillingok or Kwinhagak or Quinhagak or Larsen Bay or Levelock or Lesnoi or Kalskag or Manley Hot Springs or Manokotak or Fortuna Ledge or Mary's Igloo or Mekoryuk or Mentasta or Metlakatla or Annette Island Reserve or Naknek or Nanwalek or Napaimute or Napakiak or Napaskiak or Nelson Lagoon or Nenana or Koliganek or Stuyahok or Newhalen Village or Newtok Village or Nightmute or Nikolski or Ninilchik or Noatak or Nondalton Village or Noorvik or Nuiqsut or Nooiksut or Nulato or Nunakauyarmiut or Toksook or Nunam Iqua or Nunapitchuk or Ohogamiut or Orutsararmuit or Ouzinkie or Paimiut or Pauloff or Pedro Bay or Pitka's Point or Portage Creek or Ohgsenakale or Aleut or Aleuts or Qagan Tayagungin or Qawalangin or Unalaska or Salamatoff or Savoonga or Scammon Bay or Selawik or Seldovia or Shageluk or Shaktoolik or Shishmaref or Shungnak or (Sitka not spruce) or Skagway Village or Sleetmute or Naknek or Sun'aq or (Kodiak not bear*) or Shoonaq' or Takotna or Tanacross or Tanana or Tangirnaq or Tatitlek or Tazlina or Telida Village or Tetlin or Tlingit or Haida or Togiak or Tuluksak or Tuntutuliak or Tununak or Tyonek or Ugashik or Umkumiute or Unalakleet or (Village adj3 (Grayling or Circle or Lime or Nikolai or Wales or Platinum or Wainwright or Ambler or Unga or Teller or White Mountain)) or Wrangell or Yakutat or Yupiit or Yup'ik)).mp.) not (geolog* or thermokarst* or seismic* or geomorphology).mp. | 5723 |
| 5 | (A' ani or Haaninin or Atsina or Gros Ventre or Acopsel or Tlacopsel or Lacopsel or Ahtna or Ahtena or Akenitsi or Occaneechi or Akokisa or Horcoquisa or Orcoquizas or Aleut or Unangax or Unangan or Alibamu or Alabama Alsea or Alutiiq or Sugpiag or Pacific Yupik or Amahami or Awaxawi or Androscoggin or Arosaguntacook or Ameriscoggin or Anishinaabeg or Chippewa or Anihsinape or Saulteaux or Apalachee or Aranama or Texan Coahuilteca or Tamique or Arikara or Sahnish or Ree or Arickaree or Adakadaho or Assiniboine or Hohe or Nakota or Nakoda or Nakona or Atsa' Kudok-wa or Awatixa or Bannock or Snake Indians or Bidai or Ishak or Quasmigdo or Biloxi or Blackfoot Confederacy or Niitsitapi or Sikasikaitsitapi or Cahto or Kato or Kaipomo or Cahuilla or Ivilyuqaletem or Ivilyuat or Catawba or Inna or Iswa or Chemehuevi or Chickasaw or Chilula Chimakum or Aqokulo or Chimariko or Chiricahua or Tsokanende or Chitimacha or Chetimachan or Sitimacha or Chowanoke or Roanoke or Chumash or Ciboney or Taino Ciwat or Clatsop or Coos or Coosa or Uchis or Chiaha or Coste or Tali or Talisi or Coquille or Kokwell or Coso or Cowlitz or Taitnapam or Crow Nation or Absaroka or Cui Ui Ticutta or Cupeno or Kuupangaxwichem or Cupa or Cup' ig or Nunivak or Dakota Oyate or Lakota or Nakota or Santee or Teton or Sioux or Deadose or Deg Xinag or Deg Xit' an or Kaiyuhkhotana or Deg Hit' an or Dena' ina or Tanaina or Dichinanek' Hwt' ana or Upper Kuskokwim Athabascans or Kolchan or Goltsan or Tundra Kolosh or Do lkabya or Western Yavapai or Duwamish or Esselen or Eyak or Gidi' tikadii or Guwevkabaya or Southeast Yavapai or Gwich' in or Kutchin or Haida or Xaadas or Xaat or Halchidhoma or Havasupai or Green Water People or Hiratsa or Hiraaca or Ho-chaaqa or Winnebago or Holikachuk or Innoko or Tlegon-khotana or Hopi or Houma - Louisiana or Huaco or Waco or Hualapai or Hupa or Natinixwe or Natinook-wa or Hwech' in or ((Delaware or Iowa or Spokane or Miami or Arkansas or Han) adj3 (reservation* or tribe or tribes or tribal or Indian*)) or Hankutchin or Iroquois Confederacy or Hodinoso ni or Illinois Confedera* or Ilinoweg or Illini or Inupiat or Inuit or Ioway or Baxoje or Jicarilla or Juaneno or Acjachemen or Jumano or Kalapuya or Clackama or Kalispel or Pend d' Oreilles or Qlispe or Karuk or Karok or Chum-ne or Katkoc or Kaw or Kansa or Kanza or Kawaiisu or Nuwa or Kennebec or Kinipekw Kittitas or Klickitat or Qwu' lh-hwai-pum or Awi-adshi or Mahane or Wahnookt or Koa' aga' itoka or Keresan or Kichai or Kitsai or Keechi or K' itaish or Kiowa or Gaigwu or Cauigu or Kutjau or Kwu-da or Tep-da or Kitanemuk or Kittitas or Klickitat or Qwu' lh-hwai-pum or Awi-adshi or Mahane or Wahnookt or Koa' aga' itoka or Konkow or Koop Ticutta or Koyukon or Ktunaxa or Kootenai or Flathead or Kucadikadi or Kotsa' va or Kumeyaay or Tipai-Ipai or Kamia or Diegueno or Kwapa or Cocopah or Cucapa or Xawitt kwnchawaay or Lassik or Lenape or Leni-Lenape or Lipan or Luiseno or Payomkawichum or Madqwadabaya or Desert Yavapai or Mahican or Mohicans or Makah or Makuhadokado or Maliseet or Wolistoqiag or Manahoac or Mahock or Meipontsky or Mandan or Mattole or Bear River or Tul' bush or Ni' ekeni or Meherrin or Menominee or Mackinac or Mescalero or myaamiaki or Kickapoo or Twigtwee or Missouria or Miwok or Miwuk or Moadokado or Modoc or Mohave or Aha Makhav or Mohawk or Kaneng' hega or Molala or Molale or Molele or Mono or Nyyhmy or Moosonee or Moose Cree or Monsonis or Multnomah or Chinook or Nabedache or Nabaydacu or Wawadishe or Nabiltse or Applegate or Dakubetede or Nacho Nyak Dun or Tutchone or Nacono or Na' isha or Nanticoke or Navajo or Ndee or Nial or Niimiipu or Nez Perce or Watapala or Watapahlute or Nisenan or Nisqually or Nomlaki or Noamlakee or Central Wintun or Nongatl or Nottoway or Cheroenhaka or Northern Cheyenne or Ohlone or Costanoan or Omaha or O' odham or Pima or Tahono O' odham or Papago or Osage or Otoe or Otse or Ozav Dika or Palus or Passamaquoddy or Pestomuhkati or Patiri or Petaros or Pastia or Patwin or Southern Wintun or Pawnee or Panis or Skidi or Pedee or Penobscot or Petun Piipaash or Kokmalik' op or Piscataway or Piscatawa or Doeg or Conoy or Pit River or Pomo or Kashaya or Ponca or Ponka or Pottawatomi or Bodewadmik or Powhatan or Puyallup or Spuyalepabs or Quapaw or Ugahxpa or Quechan or Yuma or Kwtsaan or Quileute or Salinan or Saponi or Monacan or Sapon or Eastern Blackfoot or Christanna or Sawawatodo or Serrano or Taaqtam or Maarenga' yam or Yuhaviatam or Shasta or Chasta or Sasti or Shoshone or Siletz or Sinkine or Sinkyone or Siuslaw Umpqua or Skitswish or Coeur D' Alene or Schitsu' umash or Snohomish or Snuqualmi or Sokoki or Missiquoi or Stillaguamish or Stoluckwamish or Suquamish or Sutaio or Swinomish or Skagit or Syilx or Okanagan or Sotaae or Taga Ticutta or Takelma or Dagelma or Taltushtuntede or Galice or Tanan Gwich' in or Taos or Taovaya or Tataviam or Alliklik or Tawakoni or Tahuacano or Tenino or Thawikila or Hathawekela or Fort Ancient or Tigua or Ysleta del Sur or Tillamook or Nehalem or Timbisha or Panamint or Timpanogos or Ute or Tlingit or Toi Ticutta or Tolowa or Talawa Dini' or Tongva or Gabrieleno or Fernandeno or Tobikhar or Tonkawa or Ticanwatic or Tsikip or Appalousa or Opelousa or Tsitsistas or Tubatulabal or Tukabatchee or Tula or Tunica or Tuscarora or Tomahittan or Kuskarawock or Tutelo or Tutero or Totteroy or Tutera or Yusan or Tututni or Umatilla or Umpqua or Waccamaw or Waxmaw or Wadatika or Harney Valley Paiute or Wailiki or Waluulapam or Walla Walla or Walpapi or Huipui or Wampanoag or Massasoit or Wanapum or Wappo or Washoe or Wichita or Willapa or Kwalhioqua or Wi pukba or Verde Valley Yavapai or Wintu or Northern Wintun or Wiyot or Wee' at or Weyet or Yakama or Yamosopo Tuviwarai or Yaqui or Yoeme or Yatasi or Yattasih or Yavbe' or Northwest Yavapai or Yojuane or Yokuts or Mariposa or Yuki or Yupighyt or Yup'ik or Yurok or Olekwo'l or Zuni).mp. | 100625 |
| 6 | (maori or tangata whenua or mauori or moriori or mauri or ((New Zealand or Christchurch or Aukland) and (indigenous or aboriginal or "first people*" or shaman* or tribe or tribes or tribal or clan or clans))).mp. or (exp New Zealand/ and (indigenous or aboriginal or "first people*" or shaman* or tribe or tribes or tribal or clan or clans).mp.) | 4899 |
| 7 | (exp Australia/ or (Queensland or Austral* or New South Wales or NSW or "Northern Territory" or Canberra or (Sydney not Canada) or (Melbourne not (England or United Kingdom)) or Adelaide or Tasmania or (Perth not Scotland)).mp.) and (Indigen* or Aborig* or tribe or tribal or tribes or outback or Blackfella* or Aborigin* or Indigenous* or first people* or original people).ti,ab. | 11308 |
| 8 | ("Torres Strait Island* " or Ngunnawal or Murrawarri or Alyawarre or Anmatjera or Arrernte or Gurindiji or Kunibidji or Luritja or Murrinh Patha or Pitjantjatjara or Tiwi or Waripiri or Yoingu or "Guugu Yimithirr" or Kalkadoon or Adnyamathanha or Adynyamathanha or Dieri or Kaurna or Maralinga Tjarutja or Ngarrindjeri or Narungga or Gunai or Kurnai or Kulin or " Yorta Yorta" or Bangerang or Kailtheban or Wollithiga or Moira or Ulupna or "Kwat Kwat" or "Yalaba Yalaba" or "Ngurai illiam Wurrung " or Jarrakan or Noongar or Nyungar or Nyoongar or "Pila Iguru").mp. | 2465 |
| 9 | 1 or 2 or 3 or 4 or 5 or 6 or 7 or 8 | 175435 |
| 10 | ("time trade-off" or (TTO not "tea tree") or "standard gamble" or "health utility" or hui or hui2 or "hui 2" or hui3 or "hui 3" or eq-5d-3L or eq-5d-5L or eq5d* or "eq 5d*" or euroqol* or sf-6d* or sf6d* or short form 6-d or QWB or "quality of well-being" or 15-d or "health utilities index" or CHU-9D or "child health index" or AQoL or "adult quality of life" or "eortc qlq c30").mp. | 27737 |
| 11 | ("preference base*" or (("quality of life" or qol) and "self report*") or "preference weight*" or "health utilit*" or "valuation weight*" or "preference value*" or "quality adjusted life year*" or "qaly weight*" or ("health state*" adj5 (valu* or rank* or preference*)) or ((HR-PRO or HRPRO) and HR-PRO)).mp. | 46195 |
| 12 | exp "Quality of Life"/ and "self report*".mp. | 15024 |
| 13 | 10 or 11 or 12 | 67107 |
| 14 | 9 and 13 | 426 |
| 15 | (instrument$ or measure$ or "classification system$" or "health state classification$" or "descriptive system$" or index or indices or indexes or tool$).mp. or exp "Surveys and Questionnaires"/ or questionnaire$.mp. or scale$.mp. | 7483186 |
| 16 | ("preference base$" or utilit$ or "preference weight$" or "valuation weight$" or "preference valu$" or "qaly weight$" or "health state$" or valu$ or preference$ or rank$ or ("health state$" adj5 (valu$ or preference$ or rank$))).mp. | 3260524 |
| 17 | "quality of life"/ or ("quality of life" or qol or hrqol or hrql).mp. or "health status".mp. or "quality adjusted life year*".mp. or qaly*.mp. | 561585 |
| 18 | 15 and 16 and 17 | 61730 |
| 19 | 9 and 18 | 418 |
| 20 | 14 or 19 | 756 |
| 21 | ((HUI or HAN) adj3 (Qinghai or Tibet* or Chinese)).mp. or China.ti,ab. or (hui adj2 meeting*).mp. or (hui adj2 gathering*).mp. or Animals Laboratory/ or animal*.mp. | 7618786 |
| 22 | 20 not 21 | 713 |

**Embase <1974 to 2022 August 30>**

| # | Search Statement | Results |
| --- | --- | --- |
| 1 | indigenous people/ or alaska native/ or american indian/ or canadian aboriginal/ or first nation/ or inuit/ or indigenous health care/ or (first nation or first nations or first people* or original people* or "first inhabitant*" or original inhabitant* or autochtone or aborigin* or indigenous or metis or inuit or innu or inuk or inuvialuit or "american indian*" or "native american*" or amerindian).mp. | 89567 |
| 2 | ((Athapaskan or Saulteaux or Wakashan or Cree or Dene or Inuit or Inuk or Inuvialuit* or Haida or Ktunaxa or Tsimshian or Gitsxan or Nisga'a or Haisla or Heiltsuk or Oweenkeno or Kwakwaka'wakw or Nuu chah nulth or Tsilhqot'in or Dakelh or Wet'suwet'en or Sekani or Dunne-za or Dene or Tahltan or Kaska or Tagish or Tutchone or Nuxalk or Salish or Stl'atlimc or Nlaka'pamux or Okanagan or Sec wepmc or Tlingit or Anishinaabe or Blackfoot or Nakoda or Tasttine or Tsuu T'inia or Gwich'in or Han or Tagish or Tutchone or Algonquin or Nipissing or Ojibwa or Potawatomi or Innu or Maliseet or Mi'kmaq or Micmac or Passamaquoddy or Haudenosaunee or Cayuga or Mohawk or Oneida or Onodaga or Seneca or Tuscarora or Wyandot or Aboriginal* or Indigenous* or Metis or red road or "on reserve" or off-reserve or First Nation or First Nations or Amerindian or (urban adj3 (Indian* or Native* or Aboriginal*)) or ethnomedicine or country food* or residential school*).mp. or ((native american medicine/ or traditional medicine*.mp.) not Chinese.mp.) or exp Shamanism/ or shaman*.mp. or traditional heal*.mp. or traditional food*.mp. or medicine [man.mp](http://man.mp/). or medicine [woman.mp](http://woman.mp/). or autochtone*.mp. or (Native* adj1 (man or men or women or woman or boy* or girl* or adolescent* or youth or youths or person* or adult or people* or Indian* or Nation or tribe* or tribal or band or bands)).mp.) and (exp Canada/ or (Canad* or British Columbia or Columbie Britannique or Alberta or Saskatchewan or Manitoba or Ontario or Quebec or Nova Scotia or New Brunswick or Newfoundland or Labrador or Prince Edward Island or Yukon Territory or NWT or Northwest Territories or Nunavut or Nunavik or Nunatsiavut or NunatuKavut).mp.) | 8791 |
| 3 | "sami (people)"/ or (Saami or Sampi or (Sami not Ulus) or Samis or Southernsami* or Umesami* or Pitesami* or Lulesami* or Northernsami* or Enaresami* or Kolasami* or Lapp or Lapps or Lappish or Lappland or (Lapland* not longspur) or Lappalainen* or Saamelainen* or reindeer herd* or reindeer culture* or reindeer pastoral* or Lappbys or Samebys or reinbeitesdistrikt or paliskunta or siida).mp. or (((Fennoscandia or Finnmark or Scandinavia or Nordic or Sweden or Norway or Finland or Swedish or Finnish or Norwegian or Norge or Svensk* or Suomi or Barents Region or (Kola not (garcinia or gotu)) or Arctic Europe* or Polar Europe* or North* Europ*).mp. or Finland/ or Norway/ or Sweden/) and ((traditional adj2 (food* or heal* or medicine* or shaman*)) or (Indigen* adj3 (people* or person* or mother* or father* or parent* or child* or boy or boys or girl* or youth* or healer* or famil* or herder*))).mp.) | 1979 |
| 4 | ((("inupiat (people)"/ or eskimo-aleut people/ or "aleut (people)"/ or exp eskimo/) and (Alaska/ or Alaska*.mp.)) or (((Anvik or Barrow or Bethel or Buckland or Cantwell or Chilcoot or Clarkes Point or Cordova or Craig or Crooked Creek or Deering or Dot Lake or Douglas or Eagle or Eek Elim or English Bay or Evansville or False Pass or Galena or Louden or Gambel or Georgetown or (Haida not (Haida Gwaii or Skidegate or Charlotte or Canada)) or Haines or Hamilton or Healy Lake or Holy Cross or Hughes or Kake or King Island or King Salmon or Kotlik or Kwethluik or Marshall or McGrath or Minto or Nome or Northway or Old Harbor or Oscarville or Sheldon's Point or Perryville or (Petersburg not St Petersburg) or Pilot Point or Pilot Station or Point Hope or Point Lay or Port Graham or Port Heiden or Port Lions or Pribilof* or St Paul Island or St George Island* or Sand Point or Rampart or Red Devil or Ruby or Saxman or Saint Michael or Stebbins or Stevens or Stony River or twin Hills) adj3 (Eskimo or Indian or community or Native or traditional or Indigenous or tribe or tribes or tribal or elder or elders or people or peoples)) or ((Native Village and Alaska*) or ((Native adj2 Alaska*) not species) or "Indians/alaska" or Afognak or Agdaagux or Akhiok or Akiachak or Akiak or Akutan or Alakanuk or Alatna or Aleknagik or Algaaciq or Allakaket or Anaktuvuk or Andreafski or Angoon Community or Aniak or Arctic Village or Venetie or Asa'carsarmiut or Atka or Atmautluak or Atqasuk or Atkasook or "beaver village" or "Bill Moore* Slough" or Belkofski or Birch Creek Tribe or Brevig Mission or Chenega or Chanega or Chalkyitsik or Cheesh-Na Chistochina or Chefornak or Chevak or Chickaloon or Chignik or Chilkat or Copper Center Village or Klukwan or Chinik or Golovin or Chitina or Chuathbaluk or Kuskokwim or Chuloonawick or Curyung or Diomede or Inalik or Egegik or Eklutna or Ekuk or Ekwok or Emmonak or Bettles Field or Fort Yukon or Gakona or Goodnews Bay or Holikachuk or Gulkana or Hoonah or Hooper Bay or Huslia or Hydaburg or Igiugig or Iliamna or Inupiat or Inupiaq or Arctic Slope or Iqurmuit or Ivanoff Bay or Kaguyak or Kaktovik or Barter Island or Kalskag or Kaltag or Kanatak or Karluk or Kasaan or Kasigluk or Elders Council or Kasigluk or Kenaitze or Ketchikan or Kiana or Kipnuk or Kivalina or Klawock or Kluti Kaah or Knik Tribe or Kobuk or Kokhanok or Kongiganak or Kotlik or Kotzebue or Koyuk or Koyukuk or Kwethluk or Kwigillingok or Kwinhagak or Quinhagak or Larsen Bay or Levelock or Lesnoi or Kalskag or Manley Hot Springs or Manokotak or Fortuna Ledge or Mary's Igloo or Mekoryuk or Mentasta or Metlakatla or Annette Island Reserve or Naknek or Nanwalek or Napaimute or Napakiak or Napaskiak or Nelson Lagoon or Nenana or Koliganek or Stuyahok or Newhalen Village or Newtok Village or Nightmute or Nikolski or Ninilchik or Noatak or Nondalton Village or Noorvik or Nuiqsut or Nooiksut or Nulato or Nunakauyarmiut or Toksook or Nunam Iqua or Nunapitchuk or Ohogamiut or Orutsararmuit or Ouzinkie or Paimiut or Pauloff or Pedro Bay or Pitka's Point or Portage Creek or Ohgsenakale or Aleut or Aleuts or Qagan Tayagungin or Qawalangin or Unalaska or Salamatoff or Savoonga or Scammon Bay or Selawik or Seldovia or Shageluk or Shaktoolik or Shishmaref or Shungnak or (Sitka not spruce) or Skagway Village or Sleetmute or Naknek or Sun'aq or (Kodiak not bear*) or Shoonaq' or Takotna or Tanacross or Tanana or Tangirnaq or Tatitlek or Tazlina or Telida Village or Tetlin or Tlingit or Haida or Togiak or Tuluksak or Tuntutuliak or Tununak or Tyonek or Ugashik or Umkumiute or Unalakleet or (Village adj3 (Grayling or Circle or Lime or Nikolai or Wales or Platinum or Wainwright or Ambler or Unga or Teller or White Mountain)) or Wrangell or Yakutat or Yupiit or Yup'ik)).mp.) not (geolog* or thermokarst* or seismic* or geomorphology).mp. | 6751 |
| 5 | (A' ani or Haaninin or Atsina or Gros Ventre or Acopsel or Tlacopsel or Lacopsel or Ahtna or Ahtena or Akenitsi or Occaneechi or Akokisa or Horcoquisa or Orcoquizas or Aleut or Unangax or Unangan or Alibamu or Alabama Alsea or Alutiiq or Sugpiag or Pacific Yupik or Amahami or Awaxawi or Androscoggin or Arosaguntacook or Ameriscoggin or Anishinaabeg or Chippewa or Anihsinape or Saulteaux or Apalachee or Aranama or Texan Coahuilteca or Tamique or Arikara or Sahnish or Ree or Arickaree or Adakadaho or Assiniboine or Hohe or Nakota or Nakoda or Nakona or Atsa' Kudok-wa or Awatixa or Bannock or Snake Indians or Bidai or Ishak or Quasmigdo or Biloxi or Blackfoot Confederacy or Niitsitapi or Sikasikaitsitapi or Cahto or Kato or Kaipomo or Cahuilla or Ivilyuqaletem or Ivilyuat or Catawba or Inna or Iswa or Chemehuevi or Chickasaw or Chilula Chimakum or Aqokulo or Chimariko or Chiricahua or Tsokanende or Chitimacha or Chetimachan or Sitimacha or Chowanoke or Roanoke or Chumash or Ciboney or Taino Ciwat or Clatsop or Coos or Coosa or Uchis or Chiaha or Coste or Tali or Talisi or Coquille or Kokwell or Coso or Cowlitz or Taitnapam or Crow Nation or Absaroka or Cui Ui Ticutta or Cupeno or Kuupangaxwichem or Cupa or Cup' ig or Nunivak or Dakota Oyate or Lakota or Nakota or Santee or Teton or Sioux or Deadose or Deg Xinag or Deg Xit' an or Kaiyuhkhotana or Deg Hit' an or Dena' ina or Tanaina or Dichinanek' Hwt' ana or Upper Kuskokwim Athabascans or Kolchan or Goltsan or Tundra Kolosh or Do lkabya or Western Yavapai or Duwamish or Esselen or Eyak or Gidi' tikadii or Guwevkabaya or Southeast Yavapai or Gwich' in or Kutchin or Haida or Xaadas or Xaat or Halchidhoma or Havasupai or Green Water People or Hiratsa or Hiraaca or Ho-chaaqa or Winnebago or Holikachuk or Innoko or Tlegon-khotana or Hopi or Houma - Louisiana or Huaco or Waco or Hualapai or Hupa or Natinixwe or Natinook-wa or Hwech' in or ((Delaware or Iowa or Spokane or Miami or Arkansas or Han) adj3 (reservation* or tribe or tribes or tribal or Indian*)) or Hankutchin or Iroquois Confederacy or Hodinoso ni or Illinois Confedera* or Ilinoweg or Illini or Inupiat or Inuit or Ioway or Baxoje or Jicarilla or Juaneno or Acjachemen or Jumano or Kalapuya or Clackama or Kalispel or Pend d' Oreilles or Qlispe or Karuk or Karok or Chum-ne or Katkoc or Kaw or Kansa or Kanza or Kawaiisu or Nuwa or Kennebec or Kinipekw Kittitas or Klickitat or Qwu' lh-hwai-pum or Awi-adshi or Mahane or Wahnookt or Koa' aga' itoka or Keresan or Kichai or Kitsai or Keechi or K' itaish or Kiowa or Gaigwu or Cauigu or Kutjau or Kwu-da or Tep-da or Kitanemuk or Kittitas or Klickitat or Qwu' lh-hwai-pum or Awi-adshi or Mahane or Wahnookt or Koa' aga' itoka or Konkow or Koop Ticutta or Koyukon or Ktunaxa or Kootenai or Flathead or Kucadikadi or Kotsa' va or Kumeyaay or Tipai-Ipai or Kamia or Diegueno or Kwapa or Cocopah or Cucapa or Xawitt kwnchawaay or Lassik or Lenape or Leni-Lenape or Lipan or Luiseno or Payomkawichum or Madqwadabaya or Desert Yavapai or Mahican or Mohicans or Makah or Makuhadokado or Maliseet or Wolistoqiag or Manahoac or Mahock or Meipontsky or Mandan or Mattole or Bear River or Tul' bush or Ni' ekeni or Meherrin or Menominee or Mackinac or Mescalero or myaamiaki or Kickapoo or Twigtwee or Missouria or Miwok or Miwuk or Moadokado or Modoc or Mohave or Aha Makhav or Mohawk or Kaneng' hega or Molala or Molale or Molele or Mono or Nyyhmy or Moosonee or Moose Cree or Monsonis or Multnomah or Chinook or Nabedache or Nabaydacu or Wawadishe or Nabiltse or Applegate or Dakubetede or Nacho Nyak Dun or Tutchone or Nacono or Na' isha or Nanticoke or Navajo or Ndee or Nial or Niimiipu or Nez Perce or Watapala or Watapahlute or Nisenan or Nisqually or Nomlaki or Noamlakee or Central Wintun or Nongatl or Nottoway or Cheroenhaka or Northern Cheyenne or Ohlone or Costanoan or Omaha or O' odham or Pima or Tahono O' odham or Papago or Osage or Otoe or Otse or Ozav Dika or Palus or Passamaquoddy or Pestomuhkati or Patiri or Petaros or Pastia or Patwin or Southern Wintun or Pawnee or Panis or Skidi or Pedee or Penobscot or Petun Piipaash or Kokmalik' op or Piscataway or Piscatawa or Doeg or Conoy or Pit River or Pomo or Kashaya or Ponca or Ponka or Pottawatomi or Bodewadmik or Powhatan or Puyallup or Spuyalepabs or Quapaw or Ugahxpa or Quechan or Yuma or Kwtsaan or Quileute or Salinan or Saponi or Monacan or Sapon or Eastern Blackfoot or Christanna or Sawawatodo or Serrano or Taaqtam or Maarenga' yam or Yuhaviatam or Shasta or Chasta or Sasti or Shoshone or Siletz or Sinkine or Sinkyone or Siuslaw Umpqua or Skitswish or Coeur D' Alene or Schitsu' umash or Snohomish or Snuqualmi or Sokoki or Missiquoi or Stillaguamish or Stoluckwamish or Suquamish or Sutaio or Swinomish or Skagit or Syilx or Okanagan or Sotaae or Taga Ticutta or Takelma or Dagelma or Taltushtuntede or Galice or Tanan Gwich' in or Taos or Taovaya or Tataviam or Alliklik or Tawakoni or Tahuacano or Tenino or Thawikila or Hathawekela or Fort Ancient or Tigua or Ysleta del Sur or Tillamook or Nehalem or Timbisha or Panamint or Timpanogos or Ute or Tlingit or Toi Ticutta or Tolowa or Talawa Dini' or Tongva or Gabrieleno or Fernandeno or Tobikhar or Tonkawa or Ticanwatic or Tsikip or Appalousa or Opelousa or Tsitsistas or Tubatulabal or Tukabatchee or Tula or Tunica or Tuscarora or Tomahittan or Kuskarawock or Tutelo or Tutero or Totteroy or Tutera or Yusan or Tututni or Umatilla or Umpqua or Waccamaw or Waxmaw or Wadatika or Harney Valley Paiute or Wailiki or Waluulapam or Walla Walla or Walpapi or Huipui or Wampanoag or Massasoit or Wanapum or Wappo or Washoe or Wichita or Willapa or Kwalhioqua or Wi pukba or Verde Valley Yavapai or Wintu or Northern Wintun or Wiyot or Wee' at or Weyet or Yakama or Yamosopo Tuviwarai or Yaqui or Yoeme or Yatasi or Yattasih or Yavbe' or Northwest Yavapai or Yojuane or Yokuts or Mariposa or Yuki or Yupighyt or Yup'ik or Yurok or Olekwo'l or Zuni).mp. | 117351 |
| 6 | indigenous australian/ or taiwanese aborigine/ or (maori or tangata whenua or mauori or moriori or mauri or ((New Zealand or Christchurch or Aukland) and (indigenous or aboriginal or "first people*" or shaman* or tribe or tribes or tribal or clan or clans))).mp. or ((new zealand/ or "australia and new zealand".mp.) and (indigenous or aboriginal or "first people*" or shaman* or tribe or tribes or tribal or clan or clans).mp.) [mp=title, abstract, heading word, drug trade name, original title, device manufacturer, drug manufacturer, device trade name, keyword heading word, floating subheading word, candidate term word] | 7932 |
| 7 | (exp australia/ or "australia and new zealand"/ or Queensland.mp. or New South Wales.mp. or NSW.mp. or Northern Territory.mp. or Canberra.mp. or (Sydney not Canada).mp. or ((Melbourne not (England or United Kingdom)) or Adelaide or Tasmania or (Perth not Scotland) or Austral*).mp.) and ((Indigen* or Aborig* or tribe or tribal or tribes or outback or Blackfella* Aborigin* or Indigenous* or first people* or original people).ti,ab. or Torres Strait Island*.mp. or Ngunnawal.mp. or Murrawarri.mp. or Alyawarre.mp. or Anmatjera.mp. or Arrernte.mp. or Gurindiji.mp. or Kunibidji.mp. or Luritja.mp. or Murrinh Patha.mp. or Pitjantjatjara.mp. or Tiwi.mp. or Waripiri.mp. or Yoingu.mp. or Guugu Yimithirr.mp. or Kalkadoon.mp. or Torres Strait Islander*.mp. or Adnyamathanha.mp. or Adynyamathanha.mp. or Dieri.mp. or Kaurna.mp. or Maralinga Tjarutja.mp. or Ngarrindjeri.mp. or Narungga.mp. or Gunai.mp. or Kurnai.mp. or Kulin.mp. or Yorta Yorta.mp. or Bangerang.mp. or Kailtheban.mp. or Wollithiga.mp. or Moira.mp. or Ulupna.mp. or Kwat Kwat.mp. or Yalaba Yalaba.mp. or Ngurai illiam [wurrung.mp](http://wurrung.mp/). or Jarrakan.mp. or Noongar.mp. or Nyungar.mp. or Nyoongar.mp. or Pila Iguru.mp.) [mp=title, abstract, heading word, drug trade name, original title, device manufacturer, drug manufacturer, device trade name, keyword heading word, floating subheading word, candidate term word] | 13633 |
| 8 | 1 or 2 or 3 or 4 or 5 or 6 or 7 | 210316 |
| 9 | ("HR-PRO" or HRPRO or "HR-PRO" or "time trade-off" or (TTO not "tea tree") or "standard gamble" or "health utility" or hui or hui2 or "hui 2" or hui3 or "hui 3" or eq-5d-3L or eq-5d-5L or eq5d* or "eq 5d*" or euroqol* or sf-6d* or sf6d* or short form 6-d or QWB or "quality of well-being" or 15-d or "health utilities index" or CHU-9D or "child health index" or AQoL or "adult quality of life" or "eortc qlq c30").mp. | 48261 |
| 10 | ("preference based measure*" or (("quality of life" or qol) and "self report*")).mp. | 32883 |
| 11 | 9 or 10 | 77700 |
| 12 | 8 and 11 | 445 |
| 13 | (instrument$ or measure$ or "classification system$" or "health state classification$" or "descriptive system$" or index or indices or indexes).mp. or "quality of life index"/ or tool$.mp. or questionnaire/ or "structured questionnaire"/ or questionnaire$.mp. or scale$.mp. or "rating scale"/ | 8898753 |
| 14 | ("preference base$" or utilit$ or "preference weight$" or "valuation weight$" or "preference valu$" or "qaly weight$" or "health state$" or valu$ or preference$ or rank$ or ("health state$" adj5 (valu$ or preference$ or rank$))).mp. | 4272701 |
| 15 | "quality of life"/ or ("quality of life" or qol or hrqol or hrql).mp. or health status/ or "health status".mp. or "quality adjusted life year*".mp. or qaly*.mp. | 879263 |
| 16 | 13 and 14 and 15 | 93334 |
| 17 | 8 and 16 | 482 |
| 18 | 12 or 17 | 853 |
| 19 | ((HUI or HAN) adj3 (Qinghai or Tibet* or Chinese)).mp. or China.ti,ab. or (hui adj2 meeting*).mp. or (hui adj2 gathering*).mp. or exp experimental animal/ or animal experiment/ or "laboratory animal*".mp. or "animal stud*".mp. | 3600673 |
| 20 | 18 not 19 | 812 |

**APA PsycInfo <1806 to August Week 4 2022>**

| # | Search Statement | Results |
| --- | --- | --- |
| 1 | indigenous populations/ or alaska natives/ or american indians/ or inuit/ or exp pacific islanders/ or indigenous health care/ or hawaii natives/ or (first nation or first nations or first people* or original people* or "first inhabitant*" or original inhabitant* or autochtone or aborigin* or indigenous or metis or inuit or innu or inuk or inuvialuit or "american indian*" or "native american*" or amerindian).mp. | 31141 |
| 2 | ((Athapaskan or Saulteaux or Wakashan or Cree or Dene or Inuit or Inuk or Inuvialuit* or Haida or Ktunaxa or Tsimshian or Gitsxan or Nisga'a or Haisla or Heiltsuk or Oweenkeno or Kwakwaka'wakw or Nuu chah nulth or Tsilhqot'in or Dakelh or Wet'suwet'en or Sekani or Dunne-za or Dene or Tahltan or Kaska or Tagish or Tutchone or Nuxalk or Salish or Stl'atlimc or Nlaka'pamux or Okanagan or Sec wepmc or Tlingit or Anishinaabe or Blackfoot or Nakoda or Tasttine or Tsuu T'inia or Gwich'in or Han or Tagish or Tutchone or Algonquin or Nipissing or Ojibwa or Potawatomi or Innu or Maliseet or Mi'kmaq or Micmac or Passamaquoddy or Haudenosaunee or Cayuga or Mohawk or Oneida or Onodaga or Seneca or Tuscarora or Wyandot or Aboriginal* or Indigenous* or Metis or red road or "on reserve" or off-reserve or First Nation or First Nations or Amerindian or (urban adj3 (Indian* or Native* or Aboriginal*)) or ethnomedicine or country food* or residential school* or (traditional medicine* not Chinese)).mp. or exp Shamanism/ or shaman*.mp. or traditional heal*.mp. or traditional food*.mp. or medicine [man.mp](http://man.mp/). or medicine [woman.mp](http://woman.mp/). or autochtone*.mp. or (Native* adj1 (man or men or women or woman or boy* or girl* or adolescent* or youth or youths or person* or adult or people* or Indian* or Nation or tribe* or tribal or band or bands)).mp.) and (exp Canada/ or (Canad* or British Columbia or Columbie Britannique or Alberta or Saskatchewan or Manitoba or Ontario or Quebec or Nova Scotia or New Brunswick or Newfoundland or Labrador or Prince Edward Island or Yukon Territory or NWT or Northwest Territories or Nunavut or Nunavik or Nunatsiavut or NunatuKavut).mp.) | 3308 |
| 3 | (Saami or Sampi or (Sami not Ulus) or Samis or Southernsami* or Umesami* or Pitesami* or Lulesami* or Northernsami* or Enaresami* or Kolasami* or Lapp or Lapps or Lappish or Lappland or (Lapland* not longspur) or Lappalainen* or Saamelainen* or reindeer herd* or reindeer culture* or reindeer pastoral* or Lappbys or Samebys or reinbeitesdistrikt or paliskunta or siida).mp. or (((Fennoscandia or Finnmark or Scandinavia or Nordic or Sweden or Norway or Finland or Swedish or Finnish or Norwegian or Norge or Svensk* or Suomi or Barents Region or (Kola not (garcinia or gotu)) or Arctic Europe* or Polar Europe* or North* Europ*).mp. or Finland/ or Norway/ or Sweden/) and ((traditional adj2 (food* or heal* or medicine* or shaman*)) or (Indigen* adj3 (people* or person* or mother* or father* or parent* or child* or boy or boys or girl* or youth* or healer* or famil* or herder*))).mp.) | 545 |
| 4 | (A' ani or Haaninin or Atsina or Gros Ventre or Acopsel or Tlacopsel or Lacopsel or Ahtna or Ahtena or Akenitsi or Occaneechi or Akokisa or Horcoquisa or Orcoquizas or Aleut or Unangax or Unangan or Alibamu or Alabama Alsea or Alutiiq or Sugpiag or Pacific Yupik or Amahami or Awaxawi or Androscoggin or Arosaguntacook or Ameriscoggin or Anishinaabeg or Chippewa or Anihsinape or Saulteaux or Apalachee or Aranama or Texan Coahuilteca or Tamique or Arikara or Sahnish or Ree or Arickaree or Adakadaho or Assiniboine or Hohe or Nakota or Nakoda or Nakona or Atsa' Kudok-wa or Awatixa or Bannock or Snake Indians or Bidai or Ishak or Quasmigdo or Biloxi or Blackfoot Confederacy or Niitsitapi or Sikasikaitsitapi or Cahto or Kato or Kaipomo or Cahuilla or Ivilyuqaletem or Ivilyuat or Catawba or Inna or Iswa or Chemehuevi or Chickasaw or Chilula Chimakum or Aqokulo or Chimariko or Chiricahua or Tsokanende or Chitimacha or Chetimachan or Sitimacha or Chowanoke or Roanoke or Chumash or Ciboney or Taino Ciwat or Clatsop or Coos or Coosa or Uchis or Chiaha or Coste or Tali or Talisi or Coquille or Kokwell or Coso or Cowlitz or Taitnapam or Crow Nation or Absaroka or Cui Ui Ticutta or Cupeno or Kuupangaxwichem or Cupa or Cup' ig or Nunivak or Dakota Oyate or Lakota or Nakota or Santee or Teton or Sioux or Deadose or Deg Xinag or Deg Xit' an or Kaiyuhkhotana or Deg Hit' an or Dena' ina or Tanaina or Dichinanek' Hwt' ana or Upper Kuskokwim Athabascans or Kolchan or Goltsan or Tundra Kolosh or Do lkabya or Western Yavapai or Duwamish or Esselen or Eyak or Gidi' tikadii or Guwevkabaya or Southeast Yavapai or Gwich' in or Kutchin or Haida or Xaadas or Xaat or Halchidhoma or Havasupai or Green Water People or Hiratsa or Hiraaca or Ho-chaaqa or Winnebago or Holikachuk or Innoko or Tlegon-khotana or Hopi or Houma - Louisiana or Huaco or Waco or Hualapai or Hupa or Natinixwe or Natinook-wa or Hwech' in or ((Delaware or Iowa or Spokane or Miami or Arkansas or Han) adj3 (reservation* or tribe or tribes or tribal or Indian*)) or Hankutchin or Iroquois Confederacy or Hodinoso ni or Illinois Confedera* or Ilinoweg or Illini or Inupiat or Inuit or Ioway or Baxoje or Jicarilla or Juaneno or Acjachemen or Jumano or Kalapuya or Clackama or Kalispel or Pend d' Oreilles or Qlispe or Karuk or Karok or Chum-ne or Katkoc or Kaw or Kansa or Kanza or Kawaiisu or Nuwa or Kennebec or Kinipekw Kittitas or Klickitat or Qwu' lh-hwai-pum or Awi-adshi or Mahane or Wahnookt or Koa' aga' itoka or Keresan or Kichai or Kitsai or Keechi or K' itaish or Kiowa or Gaigwu or Cauigu or Kutjau or Kwu-da or Tep-da or Kitanemuk or Kittitas or Klickitat or Qwu' lh-hwai-pum or Awi-adshi or Mahane or Wahnookt or Koa' aga' itoka or Konkow or Koop Ticutta or Koyukon or Ktunaxa or Kootenai or Flathead or Kucadikadi or Kotsa' va or Kumeyaay or Tipai-Ipai or Kamia or Diegueno or Kwapa or Cocopah or Cucapa or Xawitt kwnchawaay or Lassik or Lenape or Leni-Lenape or Lipan or Luiseno or Payomkawichum or Madqwadabaya or Desert Yavapai or Mahican or Mohicans or Makah or Makuhadokado or Maliseet or Wolistoqiag or Manahoac or Mahock or Meipontsky or Mandan or Mattole or Bear River or Tul' bush or Ni' ekeni or Meherrin or Menominee or Mackinac or Mescalero or myaamiaki or Kickapoo or Twigtwee or Missouria or Miwok or Miwuk or Moadokado or Modoc or Mohave or Aha Makhav or Mohawk or Kaneng' hega or Molala or Molale or Molele or Mono or Nyyhmy or Moosonee or Moose Cree or Monsonis or Multnomah or Chinook or Nabedache or Nabaydacu or Wawadishe or Nabiltse or Applegate or Dakubetede or Nacho Nyak Dun or Tutchone or Nacono or Na' isha or Nanticoke or Navajo or Ndee or Nial or Niimiipu or Nez Perce or Watapala or Watapahlute or Nisenan or Nisqually or Nomlaki or Noamlakee or Central Wintun or Nongatl or Nottoway or Cheroenhaka or Northern Cheyenne or Ohlone or Costanoan or Omaha or O' odham or Pima or Tahono O' odham or Papago or Osage or Otoe or Otse or Ozav Dika or Palus or Passamaquoddy or Pestomuhkati or Patiri or Petaros or Pastia or Patwin or Southern Wintun or Pawnee or Panis or Skidi or Pedee or Penobscot or Petun Piipaash or Kokmalik' op or Piscataway or Piscatawa or Doeg or Conoy or Pit River or Pomo or Kashaya or Ponca or Ponka or Pottawatomi or Bodewadmik or Powhatan or Puyallup or Spuyalepabs or Quapaw or Ugahxpa or Quechan or Yuma or Kwtsaan or Quileute or Salinan or Saponi or Monacan or Sapon or Eastern Blackfoot or Christanna or Sawawatodo or Serrano or Taaqtam or Maarenga' yam or Yuhaviatam or Shasta or Chasta or Sasti or Shoshone or Siletz or Sinkine or Sinkyone or Siuslaw Umpqua or Skitswish or Coeur D' Alene or Schitsu' umash or Snohomish or Snuqualmi or Sokoki or Missiquoi or Stillaguamish or Stoluckwamish or Suquamish or Sutaio or Swinomish or Skagit or Syilx or Okanagan or Sotaae or Taga Ticutta or Takelma or Dagelma or Taltushtuntede or Galice or Tanan Gwich' in or Taos or Taovaya or Tataviam or Alliklik or Tawakoni or Tahuacano or Tenino or Thawikila or Hathawekela or Fort Ancient or Tigua or Ysleta del Sur or Tillamook or Nehalem or Timbisha or Panamint or Timpanogos or Ute or Tlingit or Toi Ticutta or Tolowa or Talawa Dini' or Tongva or Gabrieleno or Fernandeno or Tobikhar or Tonkawa or Ticanwatic or Tsikip or Appalousa or Opelousa or Tsitsistas or Tubatulabal or Tukabatchee or Tula or Tunica or Tuscarora or Tomahittan or Kuskarawock or Tutelo or Tutero or Totteroy or Tutera or Yusan or Tututni or Umatilla or Umpqua or Waccamaw or Waxmaw or Wadatika or Harney Valley Paiute or Wailiki or Waluulapam or Walla Walla or Walpapi or Huipui or Wampanoag or Massasoit or Wanapum or Wappo or Washoe or Wichita or Willapa or Kwalhioqua or Wi pukba or Verde Valley Yavapai or Wintu or Northern Wintun or Wiyot or Wee' at or Weyet or Yakama or Yamosopo Tuviwarai or Yaqui or Yoeme or Yatasi or Yattasih or Yavbe' or Northwest Yavapai or Yojuane or Yokuts or Mariposa or Yuki or Yupighyt or Yup'ik or Yurok or Olekwo'l or Zuni).mp. | 7808 |
| 5 | indigenous australian/ or taiwanese aborigine/ or (maori or tangata whenua or mauori or moriori or mauri or ((New Zealand or Christchurch or Aukland) and (indigenous or aboriginal or "first people*" or shaman* or tribe or tribes or tribal or clan or clans))).mp. or ((new zealand/ or "australia and new zealand".mp.) and (indigenous or aboriginal or "first people*" or shaman* or tribe or tribes or tribal or clan or clans).mp.) [mp=title, abstract, heading word, table of contents, key concepts, original title, tests & measures, mesh word] | 1922 |
| 6 | (exp australia/ or "australia and new zealand"/ or Queensland.mp. or New South Wales.mp. or NSW.mp. or Northern Territory.mp. or Canberra.mp. or (Sydney not Canada).mp. or ((Melbourne not (England or United Kingdom)) or Adelaide or Tasmania or (Perth not Scotland) or Austral*).mp.) and ((Indigen* or Aborig* or tribe or tribal or tribes or outback or Blackfella* Aborigin* or Indigenous* or first people* or original people).ti,ab. or Torres Strait Island*.mp. or Ngunnawal.mp. or Murrawarri.mp. or Alyawarre.mp. or Anmatjera.mp. or Arrernte.mp. or Gurindiji.mp. or Kunibidji.mp. or Luritja.mp. or Murrinh Patha.mp. or Pitjantjatjara.mp. or Tiwi.mp. or Waripiri.mp. or Yoingu.mp. or Guugu Yimithirr.mp. or Kalkadoon.mp. or Torres Strait Islander*.mp. or Adnyamathanha.mp. or Adynyamathanha.mp. or Dieri.mp. or Kaurna.mp. or Maralinga Tjarutja.mp. or Ngarrindjeri.mp. or Narungga.mp. or Gunai.mp. or Kurnai.mp. or Kulin.mp. or Yorta Yorta.mp. or Bangerang.mp. or Kailtheban.mp. or Wollithiga.mp. or Moira.mp. or Ulupna.mp. or Kwat Kwat.mp. or Yalaba Yalaba.mp. or Ngurai illiam [wurrung.mp](http://wurrung.mp/). or Jarrakan.mp. or Noongar.mp. or Nyungar.mp. or Nyoongar.mp. or Pila Iguru.mp.) [mp=title, abstract, heading word, table of contents, key concepts, original title, tests & measures, mesh word] | 3690 |
| 7 | 1 or 2 or 3 or 4 or 5 or 6 | 38295 |
| 8 | ("time trade-off" or (TTO not "tea tree") or "standard gamble" or "health utility" or hui or hui2 or "hui 2" or hui3 or "hui 3" or eq-5d-3L or eq-5d-5L or eq5d* or "eq 5d*" or euroqol* or sf-6d* or sf6d* or short form 6-d or QWB or "quality of well-being" or 15-d or "health utilities index" or CHU-9D or "child health index" or AQoL or "adult quality of life" or "eortc qlq c30").mp. | 6969 |
| 9 | ("preference base*" or (("quality of life" or qol) and "self report*") or "preference weight*" or "health utilit*" or "valuation weight*" or "preference value*" or "quality adjusted life year*" or "qaly weight*" or ("health state*" adj5 (valu* or rank* or preference*)) or (HR-PRO or HRPRO or HR-PRO)).mp. | 12884 |
| 10 | ("preference based measure*" or (("quality of life" or qol) and "self report*")).mp. | 9265 |
| 11 | 8 or 10 | 15483 |
| 12 | 7 and 11 | 127 |
| 13 | (instrument$ or measure$ or "classification system$" or "health state classification$" or "descriptive system$" or index or indices or indexes or tool$).mp. or questionnaires/ or questionnaire$.mp. or scale$.mp. or exp "rating scales"/ | 1907345 |
| 14 | ("preference base$" or utilit$ or "preference weight$" or "valuation weight$" or "preference valu$" or "qaly weight$" or "health state$" or valu$ or preference$ or rank$ or ("health state$" adj5 (valu$ or preference$ or rank$))).mp. | 632765 |
| 15 | "exp quality of life"/ or "quality of life measures"/ or ("quality of life" or qol or hrqol or hrql).mp. or exp health status/ or "health status".mp. or "quality adjusted life year*".mp. or qaly*.mp. | 137813 |
| 16 | 13 and 14 and 15 | 14988 |
| 17 | 7 and 16 | 108 |
| 18 | 12 or 17 | 228 |
| 19 | ((HUI or HAN) adj3 (Qinghai or Tibet* or Chinese)).mp. or China.ti,ab. or (hui adj2 meeting*).mp. or (hui adj2 gathering*).mp. or exp experimental animal/ or animal experiment/ or "laboratory animal*".mp. or "animal stud*".mp. | 43605 |
| 20 | 18 not 19 | 218 |
| 21 | remove duplicates from 20 | 218 |

**Global Health <1910 to 2022 Week 34>**

| # | Search Statement | Results |
| --- | --- | --- |
| 1 | (first nation or first nations or first people* or original people* or "first inhabitant*" or original inhabitant* or autochtone or aborigin* or indigenous or metis or inuit or innu or inuk or inuvialuit or american indian* or native american*).mp. | 55482 |
| 2 | (Athapaskan or Saulteaux or Wakashan or Cree or Dene or Inuit or Inuk or Inuvialuit* or Haida or Ktunaxa or Tsimshian or Gitsxan or Nisga'a or Haisla or Heiltsuk or Oweenkeno or Kwakwaka'wakw or Nuu chah nulth or Tsilhqot'in or Dakelh or Wet'suwet'en or Sekani or Dunne-za or Dene or Tahltan or Kaska or Tagish or Tutchone or Nuxalk or Salish or Stl'atlimc or Nlaka'pamux or Okanagan or Sec wepmc or Tlingit or Anishinaabe or Blackfoot or Nakoda or Tasttine or Tsuu T'inia or Gwich'in or Han or Tagish or Tutchone or Algonquin or Nipissing or Ojibwa or Potawatomi or Innu or Maliseet or Mi'kmaq or Micmac or Passamaquoddy or Haudenosaunee or Cayuga or Mohawk or Oneida or Onodaga or Seneca or Tuscarora or Wyandot or Aboriginal* or Indigenous* or Metis or red road or "on reserve" or off-reserve or First Nation or First Nations or Amerindian or (urban adj3 (Indian* or Native* or Aboriginal*)) or ethnomedicine or country food* or residential school* or (traditional medicine* not Chinese) or shaman* or traditional heal* or traditional food* or medicine man or medicine woman or autochtone* or (Native* adj1 (man or men or women or woman or boy* or girl* or adolescent* or youth or youths or person* or adult or people* or Indian* or Nation or tribe* or tribal or band or bands))).mp. and (exp Canada/ or (Canad* or British Columbia or Columbie Britannique or Alberta or Saskatchewan or Manitoba or Ontario or Quebec or Nova Scotia or New Brunswick or Newfoundland or Labrador or Prince Edward Island or Yukon Territory or NWT or Northwest Territories or Nunavut or Nunavik or Nunatsiavut or NunatuKavut).mp.) | 4138 |
| 3 | (Saami or Sampi or (Sami not Ulus) or Samis or Southernsami* or Umesami* or Pitesami* or Lulesami* or Northernsami* or Enaresami* or Kolasami* or Lapp or Lapps or Lappish or Lappland or (Lapland* not longspur) or Lappalainen* or Saamelainen* or reindeer herd* or reindeer culture* or reindeer pastoral* or Lappbys or Samebys or reinbeitesdistrikt or paliskunta or siida or ((Fennoscandia or Finnmark or Scandinavia or Nordic or Sweden or Norway or Finland or Swedish or Finnish or Norwegian or Norge or Svensk* or Suomi or Barents Region or (Kola not (garcinia or gotu)) or Arctic Europe* or Polar Europe* or North* Europ*) and ((traditional adj2 (food* or heal* or medicine* or shaman*)) or (Indigen* adj3 (people* or person* or mother* or father* or parent* or child* or boy or boys or girl* or youth* or healer* or famil* or herder*))))).mp. | 959 |
| 4 | ((alaska or (((Anvik or Barrow or Bethel or Buckland or Cantwell or Chilcoot or Clarkes Point or Cordova or Craig or Crooked Creek or Deering or Dot Lake or Douglas or Eagle or Eek Elim or English Bay or Evansville or False Pass or Galena or Louden or Gambel or Georgetown or (Haida not (Haida Gwaii or Skidegate or Charlotte or Canada)) or Haines or Hamilton or Healy Lake or Holy Cross or Hughes or Kake or King Island or King Salmon or Kotlik or Kwethluik or Marshall or McGrath or Minto or Nome or Northway or Old Harbor or Oscarville or Sheldon's Point or Perryville or (Petersburg not St Petersburg) or Pilot Point or Pilot Station or Point Hope or Point Lay or Port Graham or Port Heiden or Port Lions or Pribilof* or St Paul Island or St George Island* or Sand Point or Rampart or Red Devil or Ruby or Saxman or Saint Michael or Stebbins or Stevens or Stony River or twin Hills) adj3 (Eskimo or Indian or community or Native or traditional or Indigenous or tribe or tribes or tribal or elder or elders or people or peoples)) or ((Native Village and Alaska*) or ((Native adj2 Alaska*) not species) or "Indians/alaska" or Afognak or Agdaagux or Akhiok or Akiachak or Akiak or Akutan or Alakanuk or Alatna or Aleknagik or Algaaciq or Allakaket or Anaktuvuk or Andreafski or Angoon Community or Aniak or Arctic Village or Venetie or Asa'carsarmiut or Atka or Atmautluak or Atqasuk or Atkasook or "beaver village" or "Bill Moore* Slough" or Belkofski or Birch Creek Tribe or Brevig Mission or Chenega or Chanega or Chalkyitsik or Cheesh-Na Chistochina or Chefornak or Chevak or Chickaloon or Chignik or Chilkat or Copper Center Village or Klukwan or Chinik or Golovin or Chitina or Chuathbaluk or Kuskokwim or Chuloonawick or Curyung or Diomede or Inalik or Egegik or Eklutna or Ekuk or Ekwok or Emmonak or Bettles Field or Fort Yukon or Gakona or Goodnews Bay or Holikachuk or Gulkana or Hoonah or Hooper Bay or Huslia or Hydaburg or Igiugig or Iliamna or Inupiat or Inupiaq or Arctic Slope or Iqurmuit or Ivanoff Bay or Kaguyak or Kaktovik or Barter Island or Kalskag or Kaltag or Kanatak or Karluk or Kasaan or Kasigluk or Elders Council or Kasigluk or Kenaitze or Ketchikan or Kiana or Kipnuk or Kivalina or Klawock or Kluti Kaah or Knik Tribe or Kobuk or Kokhanok or Kongiganak or Kotlik or Kotzebue or Koyuk or Koyukuk or Kwethluk or Kwigillingok or Kwinhagak or Quinhagak or Larsen Bay or Levelock or Lesnoi or Kalskag or Manley Hot Springs or Manokotak or Fortuna Ledge or Mary's Igloo or Mekoryuk or Mentasta or Metlakatla or Annette Island Reserve or Naknek or Nanwalek or Napaimute or Napakiak or Napaskiak or Nelson Lagoon or Nenana or Koliganek or Stuyahok or Newhalen Village or Newtok Village or Nightmute or Nikolski or Ninilchik or Noatak or Nondalton Village or Noorvik or Nuiqsut or Nooiksut or Nulato or Nunakauyarmiut or Toksook or Nunam Iqua or Nunapitchuk or Ohogamiut or Orutsararmuit or Ouzinkie or Paimiut or Pauloff or Pedro Bay or Pitka's Point or Portage Creek or Ohgsenakale or Aleut or Aleuts or Qagan Tayagungin or Qawalangin or Unalaska or Salamatoff or Savoonga or Scammon Bay or Selawik or Seldovia or Shageluk or Shaktoolik or Shishmaref or Shungnak or (Sitka not spruce) or Skagway Village or Sleetmute or Naknek or Sun'aq or (Kodiak not bear*) or Shoonaq' or Takotna or Tanacross or Tanana or Tangirnaq or Tatitlek or Tazlina or Telida Village or Tetlin or Tlingit or Haida or Togiak or Tuluksak or Tuntutuliak or Tununak or Tyonek or Ugashik or Umkumiute or Unalakleet or (Village adj3 (Grayling or Circle or Lime or Nikolai or Wales or Platinum or Wainwright or Ambler or Unga or Teller or White Mountain)) or Wrangell or Yakutat or Yupiit or Yup'ik))) not (geolog* or thermokarst* or seismic* or geomorphology)).mp. | 3950 |
| 5 | (A' ani or Haaninin or Atsina or Gros Ventre or Acopsel or Tlacopsel or Lacopsel or Ahtna or Ahtena or Akenitsi or Occaneechi or Akokisa or Horcoquisa or Orcoquizas or Aleut or Unangax or Unangan or Alibamu or Alabama Alsea or Alutiiq or Sugpiag or Pacific Yupik or Amahami or Awaxawi or Androscoggin or Arosaguntacook or Ameriscoggin or Anishinaabeg or Chippewa or Anihsinape or Saulteaux or Apalachee or Aranama or Texan Coahuilteca or Tamique or Arikara or Sahnish or Ree or Arickaree or Adakadaho or Assiniboine or Hohe or Nakota or Nakoda or Nakona or Atsa' Kudok-wa or Awatixa or Bannock or Snake Indians or Bidai or Ishak or Quasmigdo or Biloxi or Blackfoot Confederacy or Niitsitapi or Sikasikaitsitapi or Cahto or Kato or Kaipomo or Cahuilla or Ivilyuqaletem or Ivilyuat or Catawba or Inna or Iswa or Chemehuevi or Chickasaw or Chilula Chimakum or Aqokulo or Chimariko or Chiricahua or Tsokanende or Chitimacha or Chetimachan or Sitimacha or Chowanoke or Roanoke or Chumash or Ciboney or Taino Ciwat or Clatsop or Coos or Coosa or Uchis or Chiaha or Coste or Tali or Talisi or Coquille or Kokwell or Coso or Cowlitz or Taitnapam or Crow Nation or Absaroka or Cui Ui Ticutta or Cupeno or Kuupangaxwichem or Cupa or Cup' ig or Nunivak or Dakota Oyate or Lakota or Nakota or Santee or Teton or Sioux or Deadose or Deg Xinag or Deg Xit' an or Kaiyuhkhotana or Deg Hit' an or Dena' ina or Tanaina or Dichinanek' Hwt' ana or Upper Kuskokwim Athabascans or Kolchan or Goltsan or Tundra Kolosh or Do lkabya or Western Yavapai or Duwamish or Esselen or Eyak or Gidi' tikadii or Guwevkabaya or Southeast Yavapai or Gwich' in or Kutchin or Haida or Xaadas or Xaat or Halchidhoma or Havasupai or Green Water People or Hiratsa or Hiraaca or Ho-chaaqa or Winnebago or Holikachuk or Innoko or Tlegon-khotana or Hopi or Houma - Louisiana or Huaco or Waco or Hualapai or Hupa or Natinixwe or Natinook-wa or Hwech' in or ((Delaware or Iowa or Spokane or Miami or Arkansas or Han) adj3 (reservation* or tribe or tribes or tribal or Indian*)) or Hankutchin or Iroquois Confederacy or Hodinoso ni or Illinois Confedera* or Ilinoweg or Illini or Inupiat or Inuit or Ioway or Baxoje or Jicarilla or Juaneno or Acjachemen or Jumano or Kalapuya or Clackama or Kalispel or Pend d' Oreilles or Qlispe or Karuk or Karok or Chum-ne or Katkoc or Kaw or Kansa or Kanza or Kawaiisu or Nuwa or Kennebec or Kinipekw Kittitas or Klickitat or Qwu' lh-hwai-pum or Awi-adshi or Mahane or Wahnookt or Koa' aga' itoka or Keresan or Kichai or Kitsai or Keechi or K' itaish or Kiowa or Gaigwu or Cauigu or Kutjau or Kwu-da or Tep-da or Kitanemuk or Kittitas or Klickitat or Qwu' lh-hwai-pum or Awi-adshi or Mahane or Wahnookt or Koa' aga' itoka or Konkow or Koop Ticutta or Koyukon or Ktunaxa or Kootenai or Flathead or Kucadikadi or Kotsa' va or Kumeyaay or Tipai-Ipai or Kamia or Diegueno or Kwapa or Cocopah or Cucapa or Xawitt kwnchawaay or Lassik or Lenape or Leni-Lenape or Lipan or Luiseno or Payomkawichum or Madqwadabaya or Desert Yavapai or Mahican or Mohicans or Makah or Makuhadokado or Maliseet or Wolistoqiag or Manahoac or Mahock or Meipontsky or Mandan or Mattole or Bear River or Tul' bush or Ni' ekeni or Meherrin or Menominee or Mackinac or Mescalero or myaamiaki or Kickapoo or Twigtwee or Missouria or Miwok or Miwuk or Moadokado or Modoc or Mohave or Aha Makhav or Mohawk or Kaneng' hega or Molala or Molale or Molele or Mono or Nyyhmy or Moosonee or Moose Cree or Monsonis or Multnomah or Chinook or Nabedache or Nabaydacu or Wawadishe or Nabiltse or Applegate or Dakubetede or Nacho Nyak Dun or Tutchone or Nacono or Na' isha or Nanticoke or Navajo or Ndee or Nial or Niimiipu or Nez Perce or Watapala or Watapahlute or Nisenan or Nisqually or Nomlaki or Noamlakee or Central Wintun or Nongatl or Nottoway or Cheroenhaka or Northern Cheyenne or Ohlone or Costanoan or Omaha or O' odham or Pima or Tahono O' odham or Papago or Osage or Otoe or Otse or Ozav Dika or Palus or Passamaquoddy or Pestomuhkati or Patiri or Petaros or Pastia or Patwin or Southern Wintun or Pawnee or Panis or Skidi or Pedee or Penobscot or Petun Piipaash or Kokmalik' op or Piscataway or Piscatawa or Doeg or Conoy or Pit River or Pomo or Kashaya or Ponca or Ponka or Pottawatomi or Bodewadmik or Powhatan or Puyallup or Spuyalepabs or Quapaw or Ugahxpa or Quechan or Yuma or Kwtsaan or Quileute or Salinan or Saponi or Monacan or Sapon or Eastern Blackfoot or Christanna or Sawawatodo or Serrano or Taaqtam or Maarenga' yam or Yuhaviatam or Shasta or Chasta or Sasti or Shoshone or Siletz or Sinkine or Sinkyone or Siuslaw Umpqua or Skitswish or Coeur D' Alene or Schitsu' umash or Snohomish or Snuqualmi or Sokoki or Missiquoi or Stillaguamish or Stoluckwamish or Suquamish or Sutaio or Swinomish or Skagit or Syilx or Okanagan or Sotaae or Taga Ticutta or Takelma or Dagelma or Taltushtuntede or Galice or Tanan Gwich' in or Taos or Taovaya or Tataviam or Alliklik or Tawakoni or Tahuacano or Tenino or Thawikila or Hathawekela or Fort Ancient or Tigua or Ysleta del Sur or Tillamook or Nehalem or Timbisha or Panamint or Timpanogos or Ute or Tlingit or Toi Ticutta or Tolowa or Talawa Dini' or Tongva or Gabrieleno or Fernandeno or Tobikhar or Tonkawa or Ticanwatic or Tsikip or Appalousa or Opelousa or Tsitsistas or Tubatulabal or Tukabatchee or Tula or Tunica or Tuscarora or Tomahittan or Kuskarawock or Tutelo or Tutero or Totteroy or Tutera or Yusan or Tututni or Umatilla or Umpqua or Waccamaw or Waxmaw or Wadatika or Harney Valley Paiute or Wailiki or Waluulapam or Walla Walla or Walpapi or Huipui or Wampanoag or Massasoit or Wanapum or Wappo or Washoe or Wichita or Willapa or Kwalhioqua or Wi pukba or Verde Valley Yavapai or Wintu or Northern Wintun or Wiyot or Wee' at or Weyet or Yakama or Yamosopo Tuviwarai or Yaqui or Yoeme or Yatasi or Yattasih or Yavbe' or Northwest Yavapai or Yojuane or Yokuts or Mariposa or Yuki or Yupighyt or Yup'ik or Yurok or Olekwo'l or Zuni).mp. | 20850 |
| 6 | (maori or tangata whenua or mauori or moriori or mauri or ((New Zealand or Christchurch or Aukland) and (indigenous or aboriginal or "first people*" or shaman* or tribe or tribes or tribal or clan or clans))).mp. or (exp New Zealand/ and (indigenous or aboriginal or "first people*" or shaman* or tribe or tribes or tribal or clan or clans).mp.) | 2413 |
| 7 | (Australia or Queensland or New South Wales or NSW or Northern Territory or Canberra or (Sydney not Canada) or ((Melbourne not (England or United Kingdom)) or Adelaide or Tasmania or (Perth not Scotland) or Austral*)).mp. and ((Indigen* or Aborig* or tribe or tribal or tribes or outback or Blackfella* Aborigin* or Indigenous* or first people* or original people).ti,ab. or Torres Strait Island*.mp. or Ngunnawal.mp. or Murrawarri.mp. or Alyawarre.mp. or Anmatjera.mp. or Arrernte.mp. or Gurindiji.mp. or Kunibidji.mp. or Luritja.mp. or Murrinh Patha.mp. or Pitjantjatjara.mp. or Tiwi.mp. or Waripiri.mp. or Yoingu.mp. or Guugu Yimithirr.mp. or Kalkadoon.mp. or Torres Strait Islander*.mp. or Adnyamathanha.mp. or Adynyamathanha.mp. or Dieri.mp. or Kaurna.mp. or Maralinga Tjarutja.mp. or Ngarrindjeri.mp. or Narungga.mp. or Gunai.mp. or Kurnai.mp. or Kulin.mp. or Yorta Yorta.mp. or Bangerang.mp. or Kailtheban.mp. or Wollithiga.mp. or Moira.mp. or Ulupna.mp. or Kwat Kwat.mp. or Yalaba Yalaba.mp. or Ngurai illiam [wurrung.mp](http://wurrung.mp/). or Jarrakan.mp. or Noongar.mp. or Nyungar.mp. or Nyoongar.mp. or Pila Iguru.mp.) [mp=abstract, title, original title, heading words, cabicodes words] | 6874 |
| 8 | 1 or 2 or 3 or 4 or 5 or 6 or 7 | 78305 |
| 9 | ("time trade-off" or (TTO not "tea tree") or "standard gamble" or "health utility" or hui or hui2 or "hui 2" or hui3 or "hui 3" or eq-5d-3L or eq-5d-5L or eq5d* or "eq 5d*" or euroqol* or sf-6d* or sf6d* or short form 6-d or QWB or "quality of well-being" or 15-d or "health utilities index" or CHU-9D or "child health index" or AQoL or "adult quality of life" or "eortc qlq c30").mp. | 4153 |
| 10 | ("preference based measure*" or (("quality of life" or qol) and "self report*") or (HR-PRO or HRPRO or HR-PRO)).mp. | 2273 |
| 11 | 9 or 10 | 6265 |
| 12 | 8 and 11 | 116 |
| 13 | (instrument$ or measure$ or "classification system$" or "health state classification$" or "descriptive system$" or index or indices or indexes or tool$ or questionnaire$ or scale$).mp. | 2674862 |
| 14 | ("preference base$" or utilit$ or "preference weight$" or "valuation weight$" or "preference valu$" or "qaly weight$" or "health state$" or valu$ or preference$ or rank$ or ("health state$" adj5 (valu$ or preference$ or rank$))).mp. | 665077 |
| 15 | ("quality of life" or qol or hrqol or hrql or "health status" or "quality adjusted life year*" or qaly*).mp. | 60672 |
| 16 | 13 and 14 and 15 | 7814 |
| 17 | 8 and 16 | 151 |
| 18 | 12 or 17 | 256 |
| 19 | ((HUI or HAN) adj3 (Qinghai or Tibet* or Chinese)).mp. or China.ti,ab. or (hui adj2 meeting*).mp. or (hui adj2 gathering*).mp. or ("animal stud*" or "laboratory animal*" or (experimental adj3 animal*)).mp. | 273209 |
| 20 | 18 not 19 | 232 |

**HaPI**

| # | Search Statement | Results |
| --- | --- | --- |
| 1 | (first nation or first nations or first people* or original people* or "first inhabitant*" or original inhabitant* or autochtone or aborigin* or indigenous or metis or inuit or innu or inuk or inuvialuit or american indian* or native american*).mp. | 7775 |
| 2 | (Saami or Sampi or (Sami not Ulus) or Samis or Southernsami* or Umesami* or Pitesami* or Lulesami* or Northernsami* or Enaresami* or Kolasami* or Lapp or Lapps or Lappish or Lappland or (Lapland* not longspur) or Lappalainen* or Saamelainen* or reindeer herd* or reindeer culture* or reindeer pastoral* or Lappbys or Samebys or reinbeitesdistrikt or paliskunta or siida or ((Fennoscandia or Finnmark or Scandinavia or Nordic or Sweden or Norway or Finland or Swedish or Finnish or Norwegian or Norge or Svensk* or Suomi or Barents Region or (Kola not (garcinia or gotu)) or Arctic Europe* or Polar Europe* or North* Europ*) and ((traditional adj2 (food* or heal* or medicine* or shaman*)) or (Indigen* adj3 (people* or person* or mother* or father* or parent* or child* or boy or boys or girl* or youth* or healer* or famil* or herder*))))).mp. | 117 |
| 3 | ((alaska or (((Anvik or Barrow or Bethel or Buckland or Cantwell or Chilcoot or Clarkes Point or Cordova or Craig or Crooked Creek or Deering or Dot Lake or Douglas or Eagle or Eek Elim or English Bay or Evansville or False Pass or Galena or Louden or Gambel or Georgetown or (Haida not (Haida Gwaii or Skidegate or Charlotte or Canada)) or Haines or Hamilton or Healy Lake or Holy Cross or Hughes or Kake or King Island or King Salmon or Kotlik or Kwethluik or Marshall or McGrath or Minto or Nome or Northway or Old Harbor or Oscarville or Sheldon's Point or Perryville or (Petersburg not St Petersburg) or Pilot Point or Pilot Station or Point Hope or Point Lay or Port Graham or Port Heiden or Port Lions or Pribilof* or St Paul Island or St George Island* or Sand Point or Rampart or Red Devil or Ruby or Saxman or Saint Michael or Stebbins or Stevens or Stony River or twin Hills) adj3 (Eskimo or Indian or community or Native or traditional or Indigenous or tribe or tribes or tribal or elder or elders or people or peoples)) or ((Native Village and Alaska*) or ((Native adj2 Alaska*) not species) or "Indians/alaska" or Afognak or Agdaagux or Akhiok or Akiachak or Akiak or Akutan or Alakanuk or Alatna or Aleknagik or Algaaciq or Allakaket or Anaktuvuk or Andreafski or Angoon Community or Aniak or Arctic Village or Venetie or Asa'carsarmiut or Atka or Atmautluak or Atqasuk or Atkasook or "beaver village" or "Bill Moore* Slough" or Belkofski or Birch Creek Tribe or Brevig Mission or Chenega or Chanega or Chalkyitsik or Cheesh-Na Chistochina or Chefornak or Chevak or Chickaloon or Chignik or Chilkat or Copper Center Village or Klukwan or Chinik or Golovin or Chitina or Chuathbaluk or Kuskokwim or Chuloonawick or Curyung or Diomede or Inalik or Egegik or Eklutna or Ekuk or Ekwok or Emmonak or Bettles Field or Fort Yukon or Gakona or Goodnews Bay or Holikachuk or Gulkana or Hoonah or Hooper Bay or Huslia or Hydaburg or Igiugig or Iliamna or Inupiat or Inupiaq or Arctic Slope or Iqurmuit or Ivanoff Bay or Kaguyak or Kaktovik or Barter Island or Kalskag or Kaltag or Kanatak or Karluk or Kasaan or Kasigluk or Elders Council or Kasigluk or Kenaitze or Ketchikan or Kiana or Kipnuk or Kivalina or Klawock or Kluti Kaah or Knik Tribe or Kobuk or Kokhanok or Kongiganak or Kotlik or Kotzebue or Koyuk or Koyukuk or Kwethluk or Kwigillingok or Kwinhagak or Quinhagak or Larsen Bay or Levelock or Lesnoi or Kalskag or Manley Hot Springs or Manokotak or Fortuna Ledge or Mary's Igloo or Mekoryuk or Mentasta or Metlakatla or Annette Island Reserve or Naknek or Nanwalek or Napaimute or Napakiak or Napaskiak or Nelson Lagoon or Nenana or Koliganek or Stuyahok or Newhalen Village or Newtok Village or Nightmute or Nikolski or Ninilchik or Noatak or Nondalton Village or Noorvik or Nuiqsut or Nooiksut or Nulato or Nunakauyarmiut or Toksook or Nunam Iqua or Nunapitchuk or Ohogamiut or Orutsararmuit or Ouzinkie or Paimiut or Pauloff or Pedro Bay or Pitka's Point or Portage Creek or Ohgsenakale or Aleut or Aleuts or Qagan Tayagungin or Qawalangin or Unalaska or Salamatoff or Savoonga or Scammon Bay or Selawik or Seldovia or Shageluk or Shaktoolik or Shishmaref or Shungnak or (Sitka not spruce) or Skagway Village or Sleetmute or Naknek or Sun'aq or (Kodiak not bear*) or Shoonaq' or Takotna or Tanacross or Tanana or Tangirnaq or Tatitlek or Tazlina or Telida Village or Tetlin or Tlingit or Haida or Togiak or Tuluksak or Tuntutuliak or Tununak or Tyonek or Ugashik or Umkumiute or Unalakleet or (Village adj3 (Grayling or Circle or Lime or Nikolai or Wales or Platinum or Wainwright or Ambler or Unga or Teller or White Mountain)) or Wrangell or Yakutat or Yupiit or Yup'ik))) not (geolog* or thermokarst* or seismic* or geomorphology)).mp. | 54 |
| 4 | (A' ani or Haaninin or Atsina or Gros Ventre or Acopsel or Tlacopsel or Lacopsel or Ahtna or Ahtena or Akenitsi or Occaneechi or Akokisa or Horcoquisa or Orcoquizas or Aleut or Unangax or Unangan or Alibamu or Alabama Alsea or Alutiiq or Sugpiag or Pacific Yupik or Amahami or Awaxawi or Androscoggin or Arosaguntacook or Ameriscoggin or Anishinaabeg or Chippewa or Anihsinape or Saulteaux or Apalachee or Aranama or Texan Coahuilteca or Tamique or Arikara or Sahnish or Ree or Arickaree or Adakadaho or Assiniboine or Hohe or Nakota or Nakoda or Nakona or Atsa' Kudok-wa or Awatixa or Bannock or Snake Indians or Bidai or Ishak or Quasmigdo or Biloxi or Blackfoot Confederacy or Niitsitapi or Sikasikaitsitapi or Cahto or Kato or Kaipomo or Cahuilla or Ivilyuqaletem or Ivilyuat or Catawba or Inna or Iswa or Chemehuevi or Chickasaw or Chilula Chimakum or Aqokulo or Chimariko or Chiricahua or Tsokanende or Chitimacha or Chetimachan or Sitimacha or Chowanoke or Roanoke or Chumash or Ciboney or Taino Ciwat or Clatsop or Coos or Coosa or Uchis or Chiaha or Coste or Tali or Talisi or Coquille or Kokwell or Coso or Cowlitz or Taitnapam or Crow Nation or Absaroka or Cui Ui Ticutta or Cupeno or Kuupangaxwichem or Cupa or Cup' ig or Nunivak or Dakota Oyate or Lakota or Nakota or Santee or Teton or Sioux or Deadose or Deg Xinag or Deg Xit' an or Kaiyuhkhotana or Deg Hit' an or Dena' ina or Tanaina or Dichinanek' Hwt' ana or Upper Kuskokwim Athabascans or Kolchan or Goltsan or Tundra Kolosh or Do lkabya or Western Yavapai or Duwamish or Esselen or Eyak or Gidi' tikadii or Guwevkabaya or Southeast Yavapai or Gwich' in or Kutchin or Haida or Xaadas or Xaat or Halchidhoma or Havasupai or Green Water People or Hiratsa or Hiraaca or Ho-chaaqa or Winnebago or Holikachuk or Innoko or Tlegon-khotana or Hopi or Houma - Louisiana or Huaco or Waco or Hualapai or Hupa or Natinixwe or Natinook-wa or Hwech' in or ((Delaware or Iowa or Spokane or Miami or Arkansas or Han) adj3 (reservation* or tribe or tribes or tribal or Indian*)) or Hankutchin or Iroquois Confederacy or Hodinoso ni or Illinois Confedera* or Ilinoweg or Illini or Inupiat or Inuit or Ioway or Baxoje or Jicarilla or Juaneno or Acjachemen or Jumano or Kalapuya or Clackama or Kalispel or Pend d' Oreilles or Qlispe or Karuk or Karok or Chum-ne or Katkoc or Kaw or Kansa or Kanza or Kawaiisu or Nuwa or Kennebec or Kinipekw Kittitas or Klickitat or Qwu' lh-hwai-pum or Awi-adshi or Mahane or Wahnookt or Koa' aga' itoka or Keresan or Kichai or Kitsai or Keechi or K' itaish or Kiowa or Gaigwu or Cauigu or Kutjau or Kwu-da or Tep-da or Kitanemuk or Kittitas or Klickitat or Qwu' lh-hwai-pum or Awi-adshi or Mahane or Wahnookt or Koa' aga' itoka or Konkow or Koop Ticutta or Koyukon or Ktunaxa or Kootenai or Flathead or Kucadikadi or Kotsa' va or Kumeyaay or Tipai-Ipai or Kamia or Diegueno or Kwapa or Cocopah or Cucapa or Xawitt kwnchawaay or Lassik or Lenape or Leni-Lenape or Lipan or Luiseno or Payomkawichum or Madqwadabaya or Desert Yavapai or Mahican or Mohicans or Makah or Makuhadokado or Maliseet or Wolistoqiag or Manahoac or Mahock or Meipontsky or Mandan or Mattole or Bear River or Tul' bush or Ni' ekeni or Meherrin or Menominee or Mackinac or Mescalero or myaamiaki or Kickapoo or Twigtwee or Missouria or Miwok or Miwuk or Moadokado or Modoc or Mohave or Aha Makhav or Mohawk or Kaneng' hega or Molala or Molale or Molele or Mono or Nyyhmy or Moosonee or Moose Cree or Monsonis or Multnomah or Chinook or Nabedache or Nabaydacu or Wawadishe or Nabiltse or Applegate or Dakubetede or Nacho Nyak Dun or Tutchone or Nacono or Na' isha or Nanticoke or Navajo or Ndee or Nial or Niimiipu or Nez Perce or Watapala or Watapahlute or Nisenan or Nisqually or Nomlaki or Noamlakee or Central Wintun or Nongatl or Nottoway or Cheroenhaka or Northern Cheyenne or Ohlone or Costanoan or Omaha or O' odham or Pima or Tahono O' odham or Papago or Osage or Otoe or Otse or Ozav Dika or Palus or Passamaquoddy or Pestomuhkati or Patiri or Petaros or Pastia or Patwin or Southern Wintun or Pawnee or Panis or Skidi or Pedee or Penobscot or Petun Piipaash or Kokmalik' op or Piscataway or Piscatawa or Doeg or Conoy or Pit River or Pomo or Kashaya or Ponca or Ponka or Pottawatomi or Bodewadmik or Powhatan or Puyallup or Spuyalepabs or Quapaw or Ugahxpa or Quechan or Yuma or Kwtsaan or Quileute or Salinan or Saponi or Monacan or Sapon or Eastern Blackfoot or Christanna or Sawawatodo or Serrano or Taaqtam or Maarenga' yam or Yuhaviatam or Shasta or Chasta or Sasti or Shoshone or Siletz or Sinkine or Sinkyone or Siuslaw Umpqua or Skitswish or Coeur D' Alene or Schitsu' umash or Snohomish or Snuqualmi or Sokoki or Missiquoi or Stillaguamish or Stoluckwamish or Suquamish or Sutaio or Swinomish or Skagit or Syilx or Okanagan or Sotaae or Taga Ticutta or Takelma or Dagelma or Taltushtuntede or Galice or Tanan Gwich' in or Taos or Taovaya or Tataviam or Alliklik or Tawakoni or Tahuacano or Tenino or Thawikila or Hathawekela or Fort Ancient or Tigua or Ysleta del Sur or Tillamook or Nehalem or Timbisha or Panamint or Timpanogos or Ute or Tlingit or Toi Ticutta or Tolowa or Talawa Dini' or Tongva or Gabrieleno or Fernandeno or Tobikhar or Tonkawa or Ticanwatic or Tsikip or Appalousa or Opelousa or Tsitsistas or Tubatulabal or Tukabatchee or Tula or Tunica or Tuscarora or Tomahittan or Kuskarawock or Tutelo or Tutero or Totteroy or Tutera or Yusan or Tututni or Umatilla or Umpqua or Waccamaw or Waxmaw or Wadatika or Harney Valley Paiute or Wailiki or Waluulapam or Walla Walla or Walpapi or Huipui or Wampanoag or Massasoit or Wanapum or Wappo or Washoe or Wichita or Willapa or Kwalhioqua or Wi pukba or Verde Valley Yavapai or Wintu or Northern Wintun or Wiyot or Wee' at or Weyet or Yakama or Yamosopo Tuviwarai or Yaqui or Yoeme or Yatasi or Yattasih or Yavbe' or Northwest Yavapai or Yojuane or Yokuts or Mariposa or Yuki or Yupighyt or Yup'ik or Yurok or Olekwo'l or Zuni).mp. | 1118 |
| 5 | (maori or tangata whenua or mauori or moriori or mauri or ((New Zealand or Christchurch or Aukland) and (indigenous or aboriginal or "first people*" or shaman* or tribe or tribes or tribal or clan or clans)) or (indigenous or aboriginal or "first people*" or shaman* or tribe or tribes or tribal or clan or clans)).mp. | 200 |
| 6 | (Australia or Queensland or New South Wales or NSW or Northern Territory or Canberra or (Sydney not Canada) or ((Melbourne not (England or United Kingdom)) or Adelaide or Tasmania or (Perth not Scotland) or Austral*)).mp. and ((Indigen* or Aborig* or tribe or tribal or tribes or outback or Blackfella* Aborigin* or Indigenous* or first people* or original people).ti,ab. or Torres Strait Island*.mp. or Ngunnawal.mp. or Murrawarri.mp. or Alyawarre.mp. or Anmatjera.mp. or Arrernte.mp. or Gurindiji.mp. or Kunibidji.mp. or Luritja.mp. or Murrinh Patha.mp. or Pitjantjatjara.mp. or Tiwi.mp. or Waripiri.mp. or Yoingu.mp. or Guugu Yimithirr.mp. or Kalkadoon.mp. or Torres Strait Islander*.mp. or Adnyamathanha.mp. or Adynyamathanha.mp. or Dieri.mp. or Kaurna.mp. or Maralinga Tjarutja.mp. or Ngarrindjeri.mp. or Narungga.mp. or Gunai.mp. or Kurnai.mp. or Kulin.mp. or Yorta Yorta.mp. or Bangerang.mp. or Kailtheban.mp. or Wollithiga.mp. or Moira.mp. or Ulupna.mp. or Kwat Kwat.mp. or Yalaba Yalaba.mp. or Ngurai illiam [wurrung.mp](http://wurrung.mp/). or Jarrakan.mp. or Noongar.mp. or Nyungar.mp. or Nyoongar.mp. or Pila Iguru.mp.) [mp=title, acronym, descriptors, measure descriptors, sample descriptors, abstract, source] | 6 |
| 7 | 1 or 2 or 3 or 4 or 5 or 6 | 9058 |
| 8 | ("time trade-off" or (TTO not "tea tree") or "standard gamble" or "health utility" or hui or hui2 or "hui 2" or hui3 or "hui 3" or eq-5d-3L or eq-5d-5L or eq5d* or "eq 5d*" or euroqol* or sf-6d* or sf6d* or short form 6-d or QWB or "quality of well-being" or 15-d or "health utilities index" or CHU-9D or "child health index" or AQoL or "adult quality of life" or "eortc qlq c30").mp. | 966 |
| 9 | ("preference based measure*" or (("quality of life" or qol) and "self report*") or ("preference weight*" or "health utilit*" or "valuation weight*" or "preference value*" or "quality adjusted life year*" or "qaly weight*" or ("health state*" adj5 (valu* or rank* or preference*)) or (HR-PRO or HRPRO or HR-PRO))).mp. | 433 |
| 10 | 8 or 9 | 1320 |
| 11 | 7 and 10 | 57 |
| 12 | (instrument* or measure* or "classification system*" or "health state classification*" or "descriptive system*" or index or indices or indexes or tool* or questionnaire* or scale*).mp. | 149418 |
| 13 | ("preference base$" or utilit$ or "preference weight$" or "valuation weight$" or "preference valu$" or "qaly weight$" or "health state$" or valu$ or preference$ or rank$ or ("health state$" adj5 (valu$ or preference$ or rank$))).mp. | 5810 |
| 14 | ("quality of life" or qol or hrqol or hrql or "health status" or "quality adjusted life year*" or qaly*).mp. | 17100 |
| 15 | 12 and 13 and 14 | 276 |
| 16 | 7 and 15 | 4 |
| 17 | 11 or 16 | 60 |
| 18 | ((HUI or HAN) adj3 (Qinghai or Tibet* or Chinese)).mp. or China.ti,ab. or (hui adj2 meeting*).mp. or (hui adj2 gathering*).mp. or ("animal stud*" or "laboratory animal*" or (experimental adj3 animal*)).mp. | 39 |
| 19 | 17 not 18 | 60 |

| **EBSCO CINAHL Plus Full Text Searched August 30, 2022**  **Search Mode: Find all my search terms**   \| # \| Query \| Results \| \| --- \| --- \| --- \| \| S1 \| MH "Indigenous Peoples+") OR (MH "Indigenous Health") OR (MH "Aboriginal Australians") OR (MH "Native Americans") OR (MH "Eskimos+") OR (MH "Inuit") OR (MH "Maori") \| 23,233 \| \| S2 \| (MH "Medicine, Native American") OR (MH "Health Services, Indigenous") OR (MH "Indigenous Health") \| 4,029 \| \| S3 \| ("first nation" or "first nations" or "first people*" or "original people*" or "first inhabitant*" or "original inhabitant*" or autochtone or aborigin* or indigenous or metis or inuit or innu or inuk or inuvialuit or "american indian*" or "native american*") \| 32,584 \| \| S4 \| (Athapaskan or Saulteaux or Wakashan or Cree or Dene or Inuit or Inuk or Inuvialuit* or Haida or Ktunaxa or Tsimshian or Gitsxan or "Nisga'a" or Haisla or Heiltsuk or Oweenkeno or "Kwakwaka'wakw" or "Nuu chah nulth" or "Tsilhqot'in" or Dakelh or "Wet'suwet'en" or Sekani or "Dunne-za" or Dene or Tahltan or Kaska or Tagish or Tutchone or Nuxalk or Salish or "Stl'atlimc" or "Nlaka'pamux" or Okanagan or "Sec wepmc" or Tlingit or Anishinaabe or Blackfoot or Nakoda or Tasttine or "Tsuu T'inia" or "Gwich'in" or Han or Tagish or Tutchone or Algonquin or Nipissing or Ojibwa or Potawatomi or Innu or Maliseet or "Mi'kmaq" or Micmac or Passamaquoddy or Haudenosaunee or Cayuga or Mohawk or Oneida or Onodaga or Seneca or Tuscarora or Wyandot or Aboriginal* or Indigenous* or Metis or red road or "on reserve" or "off-reserve" or Amerindian or (urban N3 (Indian* or Native* or Aboriginal*)) or ethnomedicine or "country food*" or "residential school*" or ("traditional medicine*" not Chinese) or shaman* or traditional heal* or "traditional food*" or "medicine man" or "medicine woman" or autochtone* or (Native* N1 (man or men or women or woman or boy* or girl* or adolescent* or youth or youths or person* or adult or people* or Indian* or Nation or tribe* or tribal or band or bands))) and ( MH "Canada" or (Canad* or "British Columbia" or "Columbie Britannique" or Alberta or Saskatchewan or Manitoba or Ontario or Quebec or "Nova Scotia" or "New Brunswick" or Newfoundland or Labrador or "Prince Edward Island" or "Yukon Territory" or NWT or "Northwest Territories" or Nunavut or Nunavik or Nunatsiavut or NunatuKavut)) \| 5,943 \| \| S5 \| (Saami or Sampi or (Sami not Ulus) or Samis or Southernsami* or Umesami* or Pitesami* or Lulesami* or Northernsami* or Enaresami* or Kolasami* or Lapp or Lapps or Lappish or Lappland or (Lapland* not longspur) or Lappalainen* or Saamelainen* or "reindeer herd*" or "reindeer culture*" or "reindeer pastoral*"or Lappbys or Samebys or reinbeitesdistrikt or paliskunta or siida or ((Fennoscandia or Finnmark or Scandinavia or Nordic or Sweden or Norway or Finland or Swedish or Finnish or Norwegian or Norge or Svensk* or Suomi or "Barents Region" or (Kola not (garcinia or gotu)) or "Arctic Europe*" or "Polar Europe*" or "North* Europ*") and ((traditional N2 (food* or heal* or medicine* or shaman*)) or (Indigen* N3 (people* or person* or mother* or father* or parent* or child* or boy or boys or girl* or youth* or healer* or famil* or herder*)) \| 11,952 \| \| S6 \| TI(“A' ani” or Absaroka or Haaninin or Atsina or Gros Ventre or Acopsel or Tlacopsel or Lacopsel or Ahtna or Ahtena or Akenitsi or Occaneechi or Akokisa or Horcoquisa or Orcoquizas or Aleut or Unangax or Unangan or Alibamu or “Alabama Alsea” or Alutiiq or Sugpiag or Pacific Yupik or Amahami or Awaxawi or Androscoggin or Arosaguntacook or Ameriscoggin or Anishinaabeg or Chippewa or Anihsinape or Saulteaux or Apalachee or Aranama or “Texan Coahuilteca” or Tamique or Arikara or Sahnish or Ree or Arickaree or Adakadaho or Assiniboine or Hohe or Nakota or Nakoda or Nakona or “Atsa' Kudok-wa” or Awatixa or Bannock or “Snake Indian*” or Bidai or Ishak or Quasmigdo or Biloxi or Blackfoot or Niitsitapi or Sikasikaitsitapi or Cahto or Kato or Kaipomo or Cahuilla or Ivilyuqaletem or Ivilyuat or Catawba or Inna or Iswa or Chemehuevi or Chickasaw or “Chilula Chimakum” or Aqokulo or Chimariko or Chiricahua or Tsokanende or Chitimacha or Chetimachan or Sitimacha or Chowanoke or Roanoke or Chumash or Ciboney or "Taino Ciwat" or Clatsop or Coos or Coosa or Uchis or Chiaha or Coste or Tali or Talisi or Coquille or Kokwell or Coso or Cowlitz or Taitnapam or “Crow Nation” or “Cui Ui Ticutta” or Cupeno or Kuupangaxwichem or Cupa or “Cup' ig” or Nunivak or “Dakota Oyate” or Lakota or Nakota or Santee or Teton or Sioux or Deadose or “Deg Xinag” or “Deg Xit' an” or Kaiyuhkhotana or “Deg Hit' an” or “Dena' ina” or Tanaina or “Dichinanek' Hwt' ana” or “Upper Kuskokwim Athabascan*” or Kolchan or Goltsan or “Tundra Kolosh” or “Do lkabya” or Duwamish or Esselen or Eyak or “Gidi' tikadii” or Guwevkabaya or “Gwich' in” or Kutchin or Haida or Xaadas or Xaat or Halchidhoma or Havasupai or “Green Water People” or Hiratsa or Hiraaca or “Ho-chaaqa” or Winnebago or Holikachuk or Innoko or “Tlegon-khotana” or Hopi or “Houma-Louisiana” or Huaco or Waco or Hualapai or Hupa or Natinixwe or “Natinook-wa” or “Hwech' in” or ((Delaware or Iowa or Spokane or Miami or Arkansas or Han or “Coeur D' Alene”) N3 (reservation* or tribe or tribes or tribal or Indian*)) or Hankutchin or “Iroquois Confederacy” or “Hodinoso ni” or “Illinois Confedera*” or Ilinoweg or Illini or Inupiat or Inuit or Ioway or Baxoje or Jicarilla or Juaneno or Acjachemen or Jumano or Kalapuya or Clackama or Kalispel or Pend d' Oreilles or Qlispe or Karuk or Karok or “Chum-ne” or Katkoc or Kaw or Kansa or Kanza or Kawaiisu or Nuwa or Kennebec or "Kinipekw Kittitas" or Klickitat or “Qwu' lh-hwai-pum” or “Awi-adshi” or Mahane or Wahnookt or “Koa' aga' itoka” or Keresan or Kichai or Kitsai or Keechi or “K' itaish” or Kiowa or Gaigwu or Cauigu or Kutjau or “Kwu-da” or “Tep-da” or Kitanemuk or Kittitas or Klickitat or “Qwu' lh-hwai-pum” or “Awi-adshi” or Mahane or Wahnookt or “Koa' aga' itoka” or Konkow or “Koop Ticutta” or Koyukon or Ktunaxa or Kootenai or Flathead or Kucadikadi or “Kotsa' va” or Kumeyaay or “Tipai-Ipai” or Kamia or Diegueno or Kwapa or Cocopah or Cucapa or “Xawitt kwnchawaay” or Lassik or Lenape or “Leni-Lenape” or Lipan or Luiseno or Payomkawichum or Madqwadabaya or Desert Yavapai or Mahican or Mohicans or Makah or Makuhadokado or Maliseet or Wolistoqiag or Manahoac or Mahock or Meipontsky or Mandan or Mattole or “Bear River” or “Tul' bush” or “Ni' ekeni” or Meherrin or Menominee or Mackinac or Mescalero or Myaamiaki or Kickapoo or Twigtwee or Missouria or Miwok or Miwuk or Moadokado or Modoc or Mohave or “Aha Makhav” or Mohawk or “Kaneng' hega” or Molala or Molale or Molele or Mono or Nyyhmy or Moosonee or Moose Cree or Monsonis or Multnomah or Chinook or Nabedache or Nabaydacu or Wawadishe or Nabiltse or Applegate or Dakubetede or “Nacho Nyak Dun” or Tutchone or Nacono or “Na' isha” or Nanticoke or Navajo or Ndee or Nial or Niimiipu or Nez Perce or Watapala or Watapahlute or Nisenan or Nisqually or Nomlaki or Noamlakee or Central Wintun or Nongatl or Nottoway or Cheroenhaka or Northern Cheyenne or Ohlone or Costanoan or Omaha or “O' odham” or Pima or Papago or Osage or Otoe or Otse or “Ozav Dika” or Palus or Passamaquoddy or Pestomuhkati or Patiri or Petaros or Pastia or Patwin or Southern Wintun or Pawnee or Panis or Skidi or Pedee or Penobscot or “Petun Piipaash” or “Kokmalik' op” or Piscataway or Piscatawa or Doeg or Conoy or “Pit River” or Pomo or Kashaya or Ponca or Ponka or Pottawatomi or Bodewadmik or Powhatan or Puyallup or Spuyalepabs or Quapaw or Ugahxpa or Quechan or Yuma or Kwtsaan or Quileute or Salinan or Saponi or Monacan or Sapon or “Eastern Blackfoot” or Christanna or Sawawatodo or Serrano or Taaqtam or “Maarenga' yam” or Yuhaviatam or Shasta or Chasta or Sasti or Shoshone or Siletz or Sinkine or Sinkyone or Siuslaw Umpqua or Skitswish or “Schitsu' umash” or Snohomish or Snuqualmi or Sokoki or Missiquoi or Stillaguamish or Stoluckwamish or Suquamish or Sutaio or Swinomish or Skagit or Syilx or Okanagan or Sotaae or “Taga Ticutta” or Takelma or Dagelma or Taltushtuntede or Galice or “Tanan Gwich' in” or Taos or Taovaya or Tataviam or Alliklik or Tawakoni or Tahuacano or Tenino or Thawikila or Hathawekela or “Fort Ancient” or Tigua or Tillamook or Nehalem or Timbisha or Panamint or Timpanogos or Ute or Tlingit or Toi Ticutta or Tolowa or “Talawa Dini'“ or Tongva or Gabrieleno or Fernandeno or Tobikhar or Tonkawa or Ticanwatic or Tsikip or Appalousa or Opelousa or Tsitsistas or Tubatulabal or Tukabatchee or Tula or Tunica or Tuscarora or Tomahittan or Kuskarawock or Tutelo or Tutero or Totteroy or Tutera or Yusan or Tututni or Umatilla or Umpqua or Waccamaw or Waxmaw or Wadatika or “Harney Valley Paiute” or Wailiki or Waluulapam or Walla Walla or Walpapi or Huipui or Wampanoag or Massasoit or Wanapum or Wappo or Washoe or Wichita or Willapa or Kwalhioqua or “Wi pukba” or “Verde Valley Yavapai” or Wintu or Northern Wintun or Wiyot or “Wee' at” or Weyet or Yakama or “Yamosopo Tuviwarai” or Yaqui or Yoeme or Yatasi or Yattasih or “Yavbe'“ or “Yavapai” or “Ysleta del Sur” or Yojuane or Yokuts or Mariposa or Yuki or Yupighyt or Yup'ik or Yupik or Yurok or “Olekwo'l” or Zuni) OR AB(“A' ani” or Absaroka or Haaninin or Atsina or Gros Ventre or Acopsel or Tlacopsel or Lacopsel or Ahtna or Ahtena or Akenitsi or Occaneechi or Akokisa or Horcoquisa or Orcoquizas or Aleut or Unangax or Unangan or Alibamu or “Alabama Alsea” or Alutiiq or Sugpiag or Pacific Yupik or Amahami or Awaxawi or Androscoggin or Arosaguntacook or Ameriscoggin or Anishinaabeg or Chippewa or Anihsinape or Saulteaux or Apalachee or Aranama or “Texan Coahuilteca” or Tamique or Arikara or Sahnish or Ree or Arickaree or Adakadaho or Assiniboine or Hohe or Nakota or Nakoda or Nakona or “Atsa' Kudok-wa” or Awatixa or Bannock or “Snake Indian*” or Bidai or Ishak or Quasmigdo or Biloxi or Blackfoot or Niitsitapi or Sikasikaitsitapi or Cahto or Kato or Kaipomo or Cahuilla or Ivilyuqaletem or Ivilyuat or Catawba or Inna or Iswa or Chemehuevi or Chickasaw or “Chilula Chimakum” or Aqokulo or Chimariko or Chiricahua or Tsokanende or Chitimacha or Chetimachan or Sitimacha or Chowanoke or Roanoke or Chumash or Ciboney or "Taino Ciwat" or Clatsop or Coos or Coosa or Uchis or Chiaha or Coste or Tali or Talisi or Coquille or Kokwell or Coso or Cowlitz or Taitnapam or “Crow Nation” or “Cui Ui Ticutta” or Cupeno or Kuupangaxwichem or Cupa or “Cup' ig” or Nunivak or “Dakota Oyate” or Lakota or Nakota or Santee or Teton or Sioux or Deadose or “Deg Xinag” or “Deg Xit' an” or Kaiyuhkhotana or “Deg Hit' an” or “Dena' ina” or Tanaina or “Dichinanek' Hwt' ana” or “Upper Kuskokwim Athabascan*” or Kolchan or Goltsan or “Tundra Kolosh” or “Do lkabya” or Duwamish or Esselen or Eyak or “Gidi' tikadii” or Guwevkabaya or “Gwich' in” or Kutchin or Haida or Xaadas or Xaat or Halchidhoma or Havasupai or “Green Water People” or Hiratsa or Hiraaca or “Ho-chaaqa” or Winnebago or Holikachuk or Innoko or “Tlegon-khotana” or Hopi or “Houma-Louisiana” or Huaco or Waco or Hualapai or Hupa or Natinixwe or “Natinook-wa” or “Hwech' in” or ((Delaware or Iowa or Spokane or Miami or Arkansas or Han or “Coeur D' Alene”) N3 (reservation* or tribe or tribes or tribal or Indian*)) or Hankutchin or “Iroquois Confederacy” or “Hodinoso ni” or “Illinois Confedera*” or Ilinoweg or Illini or Inupiat or Inuit or Ioway or Baxoje or Jicarilla or Juaneno or Acjachemen or Jumano or Kalapuya or Clackama or Kalispel or Pend d' Oreilles or Qlispe or Karuk or Karok or “Chum-ne” or Katkoc or Kaw or Kansa or Kanza or Kawaiisu or Nuwa or Kennebec or "Kinipekw Kittitas" or Klickitat or “Qwu' lh-hwai-pum” or “Awi-adshi” or Mahane or Wahnookt or “Koa' aga' itoka” or Keresan or Kichai or Kitsai or Keechi or “K' itaish” or Kiowa or Gaigwu or Cauigu or Kutjau or “Kwu-da” or “Tep-da” or Kitanemuk or Kittitas or Klickitat or “Qwu' lh-hwai-pum” or “Awi-adshi” or Mahane or Wahnookt or “Koa' aga' itoka” or Konkow or “Koop Ticutta” or Koyukon or Ktunaxa or Kootenai or Flathead or Kucadikadi or “Kotsa' va” or Kumeyaay or “Tipai-Ipai” or Kamia or Diegueno or Kwapa or Cocopah or Cucapa or “Xawitt kwnchawaay” or Lassik or Lenape or “Leni-Lenape” or Lipan or Luiseno or Payomkawichum or Madqwadabaya or Desert Yavapai or Mahican or Mohicans or Makah or Makuhadokado or Maliseet or Wolistoqiag or Manahoac or Mahock or Meipontsky or Mandan or Mattole or “Bear River” or “Tul' bush” or “Ni' ekeni” or Meherrin or Menominee or Mackinac or Mescalero or Myaamiaki or Kickapoo or Twigtwee or Missouria or Miwok or Miwuk or Moadokado or Modoc or Mohave or “Aha Makhav” or Mohawk or “Kaneng' hega” or Molala or Molale or Molele or Mono or Nyyhmy or Moosonee or Moose Cree or Monsonis or Multnomah or Chinook or Nabedache or Nabaydacu or Wawadishe or Nabiltse or Applegate or Dakubetede or “Nacho Nyak Dun” or Tutchone or Nacono or “Na' isha” or Nanticoke or Navajo or Ndee or Nial or Niimiipu or Nez Perce or Watapala or Watapahlute or Nisenan or Nisqually or Nomlaki or Noamlakee or Central Wintun or Nongatl or Nottoway or Cheroenhaka or Northern Cheyenne or Ohlone or Costanoan or Omaha or “O' odham” or Pima or Papago or Osage or Otoe or Otse or “Ozav Dika” or Palus or Passamaquoddy or Pestomuhkati or Patiri or Petaros or Pastia or Patwin or Southern Wintun or Pawnee or Panis or Skidi or Pedee or Penobscot or “Petun Piipaash” or “Kokmalik' op” or Piscataway or Piscatawa or Doeg or Conoy or “Pit River” or Pomo or Kashaya or Ponca or Ponka or Pottawatomi or Bodewadmik or Powhatan or Puyallup or Spuyalepabs or Quapaw or Ugahxpa or Quechan or Yuma or Kwtsaan or Quileute or Salinan or Saponi or Monacan or Sapon or “Eastern Blackfoot” or Christanna or Sawawatodo or Serrano or Taaqtam or “Maarenga' yam” or Yuhaviatam or Shasta or Chasta or Sasti or Shoshone or Siletz or Sinkine or Sinkyone or Siuslaw Umpqua or Skitswish or “Schitsu' umash” or Snohomish or Snuqualmi or Sokoki or Missiquoi or Stillaguamish or Stoluckwamish or Suquamish or Sutaio or Swinomish or Skagit or Syilx or Okanagan or Sotaae or “Taga Ticutta” or Takelma or Dagelma or Taltushtuntede or Galice or “Tanan Gwich' in” or Taos or Taovaya or Tataviam or Alliklik or Tawakoni or Tahuacano or Tenino or Thawikila or Hathawekela or “Fort Ancient” or Tigua or Tillamook or Nehalem or Timbisha or Panamint or Timpanogos or Ute or Tlingit or Toi Ticutta or Tolowa or “Talawa Dini'“ or Tongva or Gabrieleno or Fernandeno or Tobikhar or Tonkawa or Ticanwatic or Tsikip or Appalousa or Opelousa or Tsitsistas or Tubatulabal or Tukabatchee or Tula or Tunica or Tuscarora or Tomahittan or Kuskarawock or Tutelo or Tutero or Totteroy or Tutera or Yusan or Tututni or Umatilla or Umpqua or Waccamaw or Waxmaw or Wadatika or “Harney Valley Paiute” or Wailiki or Waluulapam or Walla Walla or Walpapi or Huipui or Wampanoag or Massasoit or Wanapum or Wappo or Washoe or Wichita or Willapa or Kwalhioqua or “Wi pukba” or “Verde Valley Yavapai” or Wintu or Northern Wintun or Wiyot or “Wee' at” or Weyet or Yakama or “Yamosopo Tuviwarai” or Yaqui or Yoeme or Yatasi or Yattasih or “Yavbe'“ or “Yavapai” or “Ysleta del Sur” or Yojuane or Yokuts or Mariposa or Yuki or Yupighyt or Yup'ik or Yupik or Yurok or “Olekwo'l” or Zuni) \| 13,386 \| \| S7 \| TI ( ((Indigen* or Aborig* or tribe or tribal or tribes or outback or Blackfella* or Aborigin* or Indigenous* or first people* or original people) ) OR AB ( ((Indigen* or Aborig* or tribe or tribal or tribes or outback or Blackfella* or Aborigin* or Indigenous* or first people* or original people) ) \| 49,320 \| \| S8 \| Australia or Queensland or New South Wales or NSW or Northern Territory or Canberra or (Sydney not Canada) or ((Melbourne not (England or United Kingdom)) or Adelaide or Tasmania or (Perth not Scotland) or Austral*)) \| 164,907 \| \| S9 \| S7 AND S8 \| 8,917 \| \| S10 \| Torres Strait Island* or Ngunnawal or Murrawarri or Alyawarre or Anmatjera or Arrernte or Gurindiji or Kunibidji or Luritja or Murrinh Patha or Pitjantjatjara or Tiwi or Waripiri or Yoingu or “Guugu Yimithirr” or Kalkadoon or “Torres Strait Islander*” or Adnyamathanha or Adynyamathanha or Dieri or Kaurna or Maralinga Tjarutja or Ngarrindjeri or Narungga or Gunai or Kurnai or Kulin or Yorta Yorta or Bangerang or Kailtheban or Wollithiga or Moira or Ulupna or “Kwat Kwat” or “Yalaba Yalaba” or Ngurai illiam wurrung or Jarrakan or Noongar or Nyungar or Nyoongar or Pila Iguru \| 3,584 \| \| S11 \| S9 OR S10 \| 10,808 \| \| S12 \| S1 OR S2 OR S3 OR S4 OR S5 OR S6 OR S11 \| 55,693 \| \| S13 \| ("time trade-off" or (TTO not "tea tree") or "standard gamble" or "health utility" or hui or hui2 or "hui 2" or hui3 or "hui 3" or eq-5d-3L or eq-5d-5L or eq5d* or "eq 5d*" or euroqol* or sf-6d* or sf6d* or short form 6-d or QWB or "quality of well-being" or 15-d or "health utilities index" or CHU-9D or "child health index" or AQoL or "adult quality of life" or "eortc qlq c30") \| 37,207 \| \| S14 \| ("preference base*" or (("quality of life" or qol) and "self report*") or "preference weight*" or "health utilit*" or "valuation weight*" or "preference value*" or "quality adjusted life year*" or "qaly weight*" or ("health state*" N5 (valu* or rank* or preference*)) or (HR-PRO or HRPRO or HR-PRO)) \| 23,879 \| \| S15 \| S13 OR S14 \| 58,389 \| \| S16 \| S12 AND S15 \| 463 \| \| S17 \| (MH "Questionnaires+") OR ( instrument* or measure* or "classification system*" or "health state classification*" or "descriptive system*" or index or indices or indexes or tool* or questionnaire* or scale or scales ) \| 2,066,267 \| \| S18 \| (MH "quality of life") or ("quality of life" or qol or hrqol or hrql) or "health status*" or "quality adjusted life year*" or qaly \| 305,507 \| \| S19 \| ("preference base*" or utility or utilities or "preference weight*" or "valuation weight*" or "preference valu*" or "qaly weight*" or "health state*" or valu* or preference* or rank* or ("health state*" N5 (valu* or preference* or rank*)) \| 785,717 \| \| S20 \| S17 AND S18 AND S19 \| 44,493 \| \| S21 \| S12 AND S20 \| 546 \| \| S22 \| S16 OR S21 \| 930 \| \| S23 \| ( ((HUI or HAN) n3 (Qinghai or Tibet* or Chinese)) or (hui n2 meeting*) or (hui n2 gathering*) or animal* ) OR TI China OR AB China \| 324,314 \| \| S24 \| s22 NOT s23 \| 877 \| |
| --- | --- | --- | --- | --- | --- | --- | --- | --- | --- | --- | --- | --- | --- | --- | --- | --- | --- | --- | --- | --- | --- | --- | --- | --- | --- | --- | --- | --- | --- | --- | --- | --- | --- | --- | --- | --- | --- | --- | --- | --- | --- | --- | --- | --- | --- | --- | --- | --- | --- | --- | --- | --- | --- | --- | --- | --- | --- | --- | --- | --- | --- | --- | --- | --- | --- | --- | --- | --- | --- | --- | --- | --- | --- | --- | --- |
|  |

**EconLit With Full Text Searched August 30, 2022**

| Limiters/Expanders |
| --- |
| Expanders - Apply equivalent subjects  Search modes - Find all my search terms |

| \| # \| Query \| Results \| \| --- \| --- \| --- \| \| S1 \| MH "Indigenous Peoples+") OR (MH "Indigenous Health") OR (MH "Aboriginal Australians") OR (MH "Native Americans") OR (MH "Eskimos+") OR (MH "Inuit") OR (MH "Maori") \| 23,233 \| \| S2 \| (MH "Medicine, Native American") OR (MH "Health Services, Indigenous") OR (MH "Indigenous Health") \| 4,029 \| \| S3 \| ("first nation" or "first nations" or "first people*" or "original people*" or "first inhabitant*" or "original inhabitant*" or autochtone or aborigin* or indigenous or metis or inuit or innu or inuk or inuvialuit or "american indian*" or "native american*") \| 32,584 \| \| S4 \| (Athapaskan or Saulteaux or Wakashan or Cree or Dene or Inuit or Inuk or Inuvialuit* or Haida or Ktunaxa or Tsimshian or Gitsxan or "Nisga'a" or Haisla or Heiltsuk or Oweenkeno or "Kwakwaka'wakw" or "Nuu chah nulth" or "Tsilhqot'in" or Dakelh or "Wet'suwet'en" or Sekani or "Dunne-za" or Dene or Tahltan or Kaska or Tagish or Tutchone or Nuxalk or Salish or "Stl'atlimc" or "Nlaka'pamux" or Okanagan or "Sec wepmc" or Tlingit or Anishinaabe or Blackfoot or Nakoda or Tasttine or "Tsuu T'inia" or "Gwich'in" or Han or Tagish or Tutchone or Algonquin or Nipissing or Ojibwa or Potawatomi or Innu or Maliseet or "Mi'kmaq" or Micmac or Passamaquoddy or Haudenosaunee or Cayuga or Mohawk or Oneida or Onodaga or Seneca or Tuscarora or Wyandot or Aboriginal* or Indigenous* or Metis or red road or "on reserve" or "off-reserve" or Amerindian or (urban N3 (Indian* or Native* or Aboriginal*)) or ethnomedicine or "country food*" or "residential school*" or ("traditional medicine*" not Chinese) or shaman* or traditional heal* or "traditional food*" or "medicine man" or "medicine woman" or autochtone* or (Native* N1 (man or men or women or woman or boy* or girl* or adolescent* or youth or youths or person* or adult or people* or Indian* or Nation or tribe* or tribal or band or bands))) and ( MH "Canada" or (Canad* or "British Columbia" or "Columbie Britannique" or Alberta or Saskatchewan or Manitoba or Ontario or Quebec or "Nova Scotia" or "New Brunswick" or Newfoundland or Labrador or "Prince Edward Island" or "Yukon Territory" or NWT or "Northwest Territories" or Nunavut or Nunavik or Nunatsiavut or NunatuKavut)) \| 5,943 \| \| S5 \| (Saami or Sampi or (Sami not Ulus) or Samis or Southernsami* or Umesami* or Pitesami* or Lulesami* or Northernsami* or Enaresami* or Kolasami* or Lapp or Lapps or Lappish or Lappland or (Lapland* not longspur) or Lappalainen* or Saamelainen* or "reindeer herd*" or "reindeer culture*" or "reindeer pastoral*"or Lappbys or Samebys or reinbeitesdistrikt or paliskunta or siida or ((Fennoscandia or Finnmark or Scandinavia or Nordic or Sweden or Norway or Finland or Swedish or Finnish or Norwegian or Norge or Svensk* or Suomi or "Barents Region" or (Kola not (garcinia or gotu)) or "Arctic Europe*" or "Polar Europe*" or "North* Europ*") and ((traditional N2 (food* or heal* or medicine* or shaman*)) or (Indigen* N3 (people* or person* or mother* or father* or parent* or child* or boy or boys or girl* or youth* or healer* or famil* or herder*)) \| 11,952 \| \| S6 \| TI(“A' ani” or Absaroka or Haaninin or Atsina or Gros Ventre or Acopsel or Tlacopsel or Lacopsel or Ahtna or Ahtena or Akenitsi or Occaneechi or Akokisa or Horcoquisa or Orcoquizas or Aleut or Unangax or Unangan or Alibamu or “Alabama Alsea” or Alutiiq or Sugpiag or Pacific Yupik or Amahami or Awaxawi or Androscoggin or Arosaguntacook or Ameriscoggin or Anishinaabeg or Chippewa or Anihsinape or Saulteaux or Apalachee or Aranama or “Texan Coahuilteca” or Tamique or Arikara or Sahnish or Ree or Arickaree or Adakadaho or Assiniboine or Hohe or Nakota or Nakoda or Nakona or “Atsa' Kudok-wa” or Awatixa or Bannock or “Snake Indian*” or Bidai or Ishak or Quasmigdo or Biloxi or Blackfoot or Niitsitapi or Sikasikaitsitapi or Cahto or Kato or Kaipomo or Cahuilla or Ivilyuqaletem or Ivilyuat or Catawba or Inna or Iswa or Chemehuevi or Chickasaw or “Chilula Chimakum” or Aqokulo or Chimariko or Chiricahua or Tsokanende or Chitimacha or Chetimachan or Sitimacha or Chowanoke or Roanoke or Chumash or Ciboney or "Taino Ciwat" or Clatsop or Coos or Coosa or Uchis or Chiaha or Coste or Tali or Talisi or Coquille or Kokwell or Coso or Cowlitz or Taitnapam or “Crow Nation” or “Cui Ui Ticutta” or Cupeno or Kuupangaxwichem or Cupa or “Cup' ig” or Nunivak or “Dakota Oyate” or Lakota or Nakota or Santee or Teton or Sioux or Deadose or “Deg Xinag” or “Deg Xit' an” or Kaiyuhkhotana or “Deg Hit' an” or “Dena' ina” or Tanaina or “Dichinanek' Hwt' ana” or “Upper Kuskokwim Athabascan*” or Kolchan or Goltsan or “Tundra Kolosh” or “Do lkabya” or Duwamish or Esselen or Eyak or “Gidi' tikadii” or Guwevkabaya or “Gwich' in” or Kutchin or Haida or Xaadas or Xaat or Halchidhoma or Havasupai or “Green Water People” or Hiratsa or Hiraaca or “Ho-chaaqa” or Winnebago or Holikachuk or Innoko or “Tlegon-khotana” or Hopi or “Houma-Louisiana” or Huaco or Waco or Hualapai or Hupa or Natinixwe or “Natinook-wa” or “Hwech' in” or ((Delaware or Iowa or Spokane or Miami or Arkansas or Han or “Coeur D' Alene”) N3 (reservation* or tribe or tribes or tribal or Indian*)) or Hankutchin or “Iroquois Confederacy” or “Hodinoso ni” or “Illinois Confedera*” or Ilinoweg or Illini or Inupiat or Inuit or Ioway or Baxoje or Jicarilla or Juaneno or Acjachemen or Jumano or Kalapuya or Clackama or Kalispel or Pend d' Oreilles or Qlispe or Karuk or Karok or “Chum-ne” or Katkoc or Kaw or Kansa or Kanza or Kawaiisu or Nuwa or Kennebec or "Kinipekw Kittitas" or Klickitat or “Qwu' lh-hwai-pum” or “Awi-adshi” or Mahane or Wahnookt or “Koa' aga' itoka” or Keresan or Kichai or Kitsai or Keechi or “K' itaish” or Kiowa or Gaigwu or Cauigu or Kutjau or “Kwu-da” or “Tep-da” or Kitanemuk or Kittitas or Klickitat or “Qwu' lh-hwai-pum” or “Awi-adshi” or Mahane or Wahnookt or “Koa' aga' itoka” or Konkow or “Koop Ticutta” or Koyukon or Ktunaxa or Kootenai or Flathead or Kucadikadi or “Kotsa' va” or Kumeyaay or “Tipai-Ipai” or Kamia or Diegueno or Kwapa or Cocopah or Cucapa or “Xawitt kwnchawaay” or Lassik or Lenape or “Leni-Lenape” or Lipan or Luiseno or Payomkawichum or Madqwadabaya or Desert Yavapai or Mahican or Mohicans or Makah or Makuhadokado or Maliseet or Wolistoqiag or Manahoac or Mahock or Meipontsky or Mandan or Mattole or “Bear River” or “Tul' bush” or “Ni' ekeni” or Meherrin or Menominee or Mackinac or Mescalero or Myaamiaki or Kickapoo or Twigtwee or Missouria or Miwok or Miwuk or Moadokado or Modoc or Mohave or “Aha Makhav” or Mohawk or “Kaneng' hega” or Molala or Molale or Molele or Mono or Nyyhmy or Moosonee or Moose Cree or Monsonis or Multnomah or Chinook or Nabedache or Nabaydacu or Wawadishe or Nabiltse or Applegate or Dakubetede or “Nacho Nyak Dun” or Tutchone or Nacono or “Na' isha” or Nanticoke or Navajo or Ndee or Nial or Niimiipu or Nez Perce or Watapala or Watapahlute or Nisenan or Nisqually or Nomlaki or Noamlakee or Central Wintun or Nongatl or Nottoway or Cheroenhaka or Northern Cheyenne or Ohlone or Costanoan or Omaha or “O' odham” or Pima or Papago or Osage or Otoe or Otse or “Ozav Dika” or Palus or Passamaquoddy or Pestomuhkati or Patiri or Petaros or Pastia or Patwin or Southern Wintun or Pawnee or Panis or Skidi or Pedee or Penobscot or “Petun Piipaash” or “Kokmalik' op” or Piscataway or Piscatawa or Doeg or Conoy or “Pit River” or Pomo or Kashaya or Ponca or Ponka or Pottawatomi or Bodewadmik or Powhatan or Puyallup or Spuyalepabs or Quapaw or Ugahxpa or Quechan or Yuma or Kwtsaan or Quileute or Salinan or Saponi or Monacan or Sapon or “Eastern Blackfoot” or Christanna or Sawawatodo or Serrano or Taaqtam or “Maarenga' yam” or Yuhaviatam or Shasta or Chasta or Sasti or Shoshone or Siletz or Sinkine or Sinkyone or Siuslaw Umpqua or Skitswish or “Schitsu' umash” or Snohomish or Snuqualmi or Sokoki or Missiquoi or Stillaguamish or Stoluckwamish or Suquamish or Sutaio or Swinomish or Skagit or Syilx or Okanagan or Sotaae or “Taga Ticutta” or Takelma or Dagelma or Taltushtuntede or Galice or “Tanan Gwich' in” or Taos or Taovaya or Tataviam or Alliklik or Tawakoni or Tahuacano or Tenino or Thawikila or Hathawekela or “Fort Ancient” or Tigua or Tillamook or Nehalem or Timbisha or Panamint or Timpanogos or Ute or Tlingit or Toi Ticutta or Tolowa or “Talawa Dini'“ or Tongva or Gabrieleno or Fernandeno or Tobikhar or Tonkawa or Ticanwatic or Tsikip or Appalousa or Opelousa or Tsitsistas or Tubatulabal or Tukabatchee or Tula or Tunica or Tuscarora or Tomahittan or Kuskarawock or Tutelo or Tutero or Totteroy or Tutera or Yusan or Tututni or Umatilla or Umpqua or Waccamaw or Waxmaw or Wadatika or “Harney Valley Paiute” or Wailiki or Waluulapam or Walla Walla or Walpapi or Huipui or Wampanoag or Massasoit or Wanapum or Wappo or Washoe or Wichita or Willapa or Kwalhioqua or “Wi pukba” or “Verde Valley Yavapai” or Wintu or Northern Wintun or Wiyot or “Wee' at” or Weyet or Yakama or “Yamosopo Tuviwarai” or Yaqui or Yoeme or Yatasi or Yattasih or “Yavbe'“ or “Yavapai” or “Ysleta del Sur” or Yojuane or Yokuts or Mariposa or Yuki or Yupighyt or Yup'ik or Yupik or Yurok or “Olekwo'l” or Zuni) OR AB(“A' ani” or Absaroka or Haaninin or Atsina or Gros Ventre or Acopsel or Tlacopsel or Lacopsel or Ahtna or Ahtena or Akenitsi or Occaneechi or Akokisa or Horcoquisa or Orcoquizas or Aleut or Unangax or Unangan or Alibamu or “Alabama Alsea” or Alutiiq or Sugpiag or Pacific Yupik or Amahami or Awaxawi or Androscoggin or Arosaguntacook or Ameriscoggin or Anishinaabeg or Chippewa or Anihsinape or Saulteaux or Apalachee or Aranama or “Texan Coahuilteca” or Tamique or Arikara or Sahnish or Ree or Arickaree or Adakadaho or Assiniboine or Hohe or Nakota or Nakoda or Nakona or “Atsa' Kudok-wa” or Awatixa or Bannock or “Snake Indian*” or Bidai or Ishak or Quasmigdo or Biloxi or Blackfoot or Niitsitapi or Sikasikaitsitapi or Cahto or Kato or Kaipomo or Cahuilla or Ivilyuqaletem or Ivilyuat or Catawba or Inna or Iswa or Chemehuevi or Chickasaw or “Chilula Chimakum” or Aqokulo or Chimariko or Chiricahua or Tsokanende or Chitimacha or Chetimachan or Sitimacha or Chowanoke or Roanoke or Chumash or Ciboney or "Taino Ciwat" or Clatsop or Coos or Coosa or Uchis or Chiaha or Coste or Tali or Talisi or Coquille or Kokwell or Coso or Cowlitz or Taitnapam or “Crow Nation” or “Cui Ui Ticutta” or Cupeno or Kuupangaxwichem or Cupa or “Cup' ig” or Nunivak or “Dakota Oyate” or Lakota or Nakota or Santee or Teton or Sioux or Deadose or “Deg Xinag” or “Deg Xit' an” or Kaiyuhkhotana or “Deg Hit' an” or “Dena' ina” or Tanaina or “Dichinanek' Hwt' ana” or “Upper Kuskokwim Athabascan*” or Kolchan or Goltsan or “Tundra Kolosh” or “Do lkabya” or Duwamish or Esselen or Eyak or “Gidi' tikadii” or Guwevkabaya or “Gwich' in” or Kutchin or Haida or Xaadas or Xaat or Halchidhoma or Havasupai or “Green Water People” or Hiratsa or Hiraaca or “Ho-chaaqa” or Winnebago or Holikachuk or Innoko or “Tlegon-khotana” or Hopi or “Houma-Louisiana” or Huaco or Waco or Hualapai or Hupa or Natinixwe or “Natinook-wa” or “Hwech' in” or ((Delaware or Iowa or Spokane or Miami or Arkansas or Han or “Coeur D' Alene”) N3 (reservation* or tribe or tribes or tribal or Indian*)) or Hankutchin or “Iroquois Confederacy” or “Hodinoso ni” or “Illinois Confedera*” or Ilinoweg or Illini or Inupiat or Inuit or Ioway or Baxoje or Jicarilla or Juaneno or Acjachemen or Jumano or Kalapuya or Clackama or Kalispel or Pend d' Oreilles or Qlispe or Karuk or Karok or “Chum-ne” or Katkoc or Kaw or Kansa or Kanza or Kawaiisu or Nuwa or Kennebec or "Kinipekw Kittitas" or Klickitat or “Qwu' lh-hwai-pum” or “Awi-adshi” or Mahane or Wahnookt or “Koa' aga' itoka” or Keresan or Kichai or Kitsai or Keechi or “K' itaish” or Kiowa or Gaigwu or Cauigu or Kutjau or “Kwu-da” or “Tep-da” or Kitanemuk or Kittitas or Klickitat or “Qwu' lh-hwai-pum” or “Awi-adshi” or Mahane or Wahnookt or “Koa' aga' itoka” or Konkow or “Koop Ticutta” or Koyukon or Ktunaxa or Kootenai or Flathead or Kucadikadi or “Kotsa' va” or Kumeyaay or “Tipai-Ipai” or Kamia or Diegueno or Kwapa or Cocopah or Cucapa or “Xawitt kwnchawaay” or Lassik or Lenape or “Leni-Lenape” or Lipan or Luiseno or Payomkawichum or Madqwadabaya or Desert Yavapai or Mahican or Mohicans or Makah or Makuhadokado or Maliseet or Wolistoqiag or Manahoac or Mahock or Meipontsky or Mandan or Mattole or “Bear River” or “Tul' bush” or “Ni' ekeni” or Meherrin or Menominee or Mackinac or Mescalero or Myaamiaki or Kickapoo or Twigtwee or Missouria or Miwok or Miwuk or Moadokado or Modoc or Mohave or “Aha Makhav” or Mohawk or “Kaneng' hega” or Molala or Molale or Molele or Mono or Nyyhmy or Moosonee or Moose Cree or Monsonis or Multnomah or Chinook or Nabedache or Nabaydacu or Wawadishe or Nabiltse or Applegate or Dakubetede or “Nacho Nyak Dun” or Tutchone or Nacono or “Na' isha” or Nanticoke or Navajo or Ndee or Nial or Niimiipu or Nez Perce or Watapala or Watapahlute or Nisenan or Nisqually or Nomlaki or Noamlakee or Central Wintun or Nongatl or Nottoway or Cheroenhaka or Northern Cheyenne or Ohlone or Costanoan or Omaha or “O' odham” or Pima or Papago or Osage or Otoe or Otse or “Ozav Dika” or Palus or Passamaquoddy or Pestomuhkati or Patiri or Petaros or Pastia or Patwin or Southern Wintun or Pawnee or Panis or Skidi or Pedee or Penobscot or “Petun Piipaash” or “Kokmalik' op” or Piscataway or Piscatawa or Doeg or Conoy or “Pit River” or Pomo or Kashaya or Ponca or Ponka or Pottawatomi or Bodewadmik or Powhatan or Puyallup or Spuyalepabs or Quapaw or Ugahxpa or Quechan or Yuma or Kwtsaan or Quileute or Salinan or Saponi or Monacan or Sapon or “Eastern Blackfoot” or Christanna or Sawawatodo or Serrano or Taaqtam or “Maarenga' yam” or Yuhaviatam or Shasta or Chasta or Sasti or Shoshone or Siletz or Sinkine or Sinkyone or Siuslaw Umpqua or Skitswish or “Schitsu' umash” or Snohomish or Snuqualmi or Sokoki or Missiquoi or Stillaguamish or Stoluckwamish or Suquamish or Sutaio or Swinomish or Skagit or Syilx or Okanagan or Sotaae or “Taga Ticutta” or Takelma or Dagelma or Taltushtuntede or Galice or “Tanan Gwich' in” or Taos or Taovaya or Tataviam or Alliklik or Tawakoni or Tahuacano or Tenino or Thawikila or Hathawekela or “Fort Ancient” or Tigua or Tillamook or Nehalem or Timbisha or Panamint or Timpanogos or Ute or Tlingit or Toi Ticutta or Tolowa or “Talawa Dini'“ or Tongva or Gabrieleno or Fernandeno or Tobikhar or Tonkawa or Ticanwatic or Tsikip or Appalousa or Opelousa or Tsitsistas or Tubatulabal or Tukabatchee or Tula or Tunica or Tuscarora or Tomahittan or Kuskarawock or Tutelo or Tutero or Totteroy or Tutera or Yusan or Tututni or Umatilla or Umpqua or Waccamaw or Waxmaw or Wadatika or “Harney Valley Paiute” or Wailiki or Waluulapam or Walla Walla or Walpapi or Huipui or Wampanoag or Massasoit or Wanapum or Wappo or Washoe or Wichita or Willapa or Kwalhioqua or “Wi pukba” or “Verde Valley Yavapai” or Wintu or Northern Wintun or Wiyot or “Wee' at” or Weyet or Yakama or “Yamosopo Tuviwarai” or Yaqui or Yoeme or Yatasi or Yattasih or “Yavbe'“ or “Yavapai” or “Ysleta del Sur” or Yojuane or Yokuts or Mariposa or Yuki or Yupighyt or Yup'ik or Yupik or Yurok or “Olekwo'l” or Zuni) \| 13,386 \| \| S7 \| TI ( ((Indigen* or Aborig* or tribe or tribal or tribes or outback or Blackfella* or Aborigin* or Indigenous* or first people* or original people) ) OR AB ( ((Indigen* or Aborig* or tribe or tribal or tribes or outback or Blackfella* or Aborigin* or Indigenous* or first people* or original people) ) \| 49,320 \| \| S8 \| Australia or Queensland or New South Wales or NSW or Northern Territory or Canberra or (Sydney not Canada) or ((Melbourne not (England or United Kingdom)) or Adelaide or Tasmania or (Perth not Scotland) or Austral*)) \| 164,907 \| \| S9 \| S7 AND S8 \| 8,917 \| \| S10 \| Torres Strait Island* or Ngunnawal or Murrawarri or Alyawarre or Anmatjera or Arrernte or Gurindiji or Kunibidji or Luritja or Murrinh Patha or Pitjantjatjara or Tiwi or Waripiri or Yoingu or “Guugu Yimithirr” or Kalkadoon or “Torres Strait Islander*” or Adnyamathanha or Adynyamathanha or Dieri or Kaurna or Maralinga Tjarutja or Ngarrindjeri or Narungga or Gunai or Kurnai or Kulin or Yorta Yorta or Bangerang or Kailtheban or Wollithiga or Moira or Ulupna or “Kwat Kwat” or “Yalaba Yalaba” or Ngurai illiam wurrung or Jarrakan or Noongar or Nyungar or Nyoongar or Pila Iguru \| 3,584 \| \| S11 \| S9 OR S10 \| 10,808 \| \| S12 \| S1 OR S2 OR S3 OR S4 OR S5 OR S6 OR S11 \| 55,693 \| \| S13 \| ("time trade-off" or (TTO not "tea tree") or "standard gamble" or "health utility" or hui or hui2 or "hui 2" or hui3 or "hui 3" or eq-5d-3L or eq-5d-5L or eq5d* or "eq 5d*" or euroqol* or sf-6d* or sf6d* or short form 6-d or QWB or "quality of well-being" or 15-d or "health utilities index" or CHU-9D or "child health index" or AQoL or "adult quality of life" or "eortc qlq c30") \| 37,207 \| \| S14 \| ("preference base*" or (("quality of life" or qol) and "self report*") or "preference weight*" or "health utilit*" or "valuation weight*" or "preference value*" or "quality adjusted life year*" or "qaly weight*" or ("health state*" N5 (valu* or rank* or preference*)) or (HR-PRO or HRPRO or HR-PRO)) \| 23,879 \| \| S15 \| S13 OR S14 \| 58,389 \| \| S16 \| S12 AND S15 \| 463 \| \| S17 \| (MH "Questionnaires+") OR ( instrument* or measure* or "classification system*" or "health state classification*" or "descriptive system*" or index or indices or indexes or tool* or questionnaire* or scale or scales ) \| 2,066,267 \| \| S18 \| (MH "quality of life") or ("quality of life" or qol or hrqol or hrql) or "health status*" or "quality adjusted life year*" or qaly \| 305,507 \| \| S19 \| ("preference base*" or utility or utilities or "preference weight*" or "valuation weight*" or "preference valu*" or "qaly weight*" or "health state*" or valu* or preference* or rank* or ("health state*" N5 (valu* or preference* or rank*)) \| 785,717 \| \| S20 \| S17 AND S18 AND S19 \| 44,493 \| \| S21 \| S12 AND S20 \| 546 \| \| S22 \| S16 OR S21 \| 930 \| \| S23 \| ( ((HUI or HAN) n3 (Qinghai or Tibet* or Chinese)) or (hui n2 meeting*) or (hui n2 gathering*) or animal* ) OR TI China OR AB China \| 324,314 \| \| S24 \| s22 NOT s23 \| 877 \| |
| --- | --- | --- | --- | --- | --- | --- | --- | --- | --- | --- | --- | --- | --- | --- | --- | --- | --- | --- | --- | --- | --- | --- | --- | --- | --- | --- | --- | --- | --- | --- | --- | --- | --- | --- | --- | --- | --- | --- | --- | --- | --- | --- | --- | --- | --- | --- | --- | --- | --- | --- | --- | --- | --- | --- | --- | --- | --- | --- | --- | --- | --- | --- | --- | --- | --- | --- | --- | --- | --- | --- | --- | --- | --- | --- | --- |

**Cochrane Library Searched September 10, 2020**

ID Search Hits

#1 (indigenous* OR amerindian* OR autochtone* OR "first nation" OR "first nations"
OR metis or inuit OR innu OR inuk OR inuvialuit OR aborigin* OR sami OR sampi OR saami
 OR Lapp OR Lapps OR Maori OR "torres strait islander" OR "american indian" OR "hawaiian
 islander" OR "alaska*native" OR "Indian reservation*" OR "red road" OR "residential
school" OR "country food" OR "medicine man" OR "medicine woman" OR "urban indian"
 OR "urban native" OR "native elder" OR tribe* OR tribal or "Indian band" OR "native
band" "native settlement" or "american indians" ):ti,ab,kw 2880

#2 MeSH descriptor: [Indians, North American] explode all trees 317

#3 MeSH descriptor: [Indigenous Peoples] 1 tree(s) exploded 3

#4 MeSH descriptor: [Health Services, Indigenous] explode all trees 51

#5 #1 or #2 or #3 or #4 2968

#6 "time trade-off" or (tto not "tee tree") 292

#7 "standard gamble" or "health utility" or QWB or HR-PRO or HRPRO or HR-PRO 793

#8 "eq-5d-3L" or "eq-5d-5L" or "euroqol-5d" or "sf-6d" or "short form 6-d" or eq5d*
 or eq-5d* or hui1 or hui2 or hui3 or hui-1 or hui-2 or hui-3 11357

#9 ("quality of well-being" or "15-d" or "health utilities index" or "CHU-9D" or
"child health index" or AQoL or "adult quality of life" or "eortc qlq c30"):ti,ab,kw 5555

#10 "preference based*" 349

#11 #6 or #7 or #8 or #9 or #10 16831

#12 #5 and #11 73

#13 instrument* or measure* or "classification system" or "classification systems" or
 "health state classification" or "descriptive system" or "descriptive systems" or index
or indices or indexes or tool or tools or questionnaire* or scale or scales 1935779

#14 (preference* or utility or utilities or "valuation weight$" or "qaly weight" or
 "health state" or "health states" or value or values or rank*) 241345

#15 "quality of life" or qol or hrqol or hrql or "health status" or "quality adjusted
 life year*" or qaly* 154304

#16 #13 and #14 and #15 29924

#17 #5 and #16 146

#18 #12 or #17 191

#19 "animal study" or "animal studies" or "laboratory animal" or "laboratory animals" or "experimental animal" or "experimental animals" 4688

#20 (china or chinese):ti,ab,kw 43751

#21 hui next 3 (gathering or meeting or Qinghai or tibet*) 3

#22 han next3 (Quinghai or tibet*) 3

#23 "flow reserve" or "Metis study" or "3 study" or "fractional flow" or TTTrials
or TTFields or "III Study" 23561

#24 # 19 or #20 or #21 or #22 or #23 256200

#25 #18 not #24 80

**SCOPUS Searched September 20, 2020 Results =218**

( ( ( TITLE-ABS-KEY ( "time trade-off" OR ( tto AND NOT "tea tree" ) OR "standard gamble" OR "health utility" OR hui OR hui2 OR "hui 2" OR hui3 OR "hui 3" OR eq-5d-3l OR eq-5d-5l OR eq5d* OR "eq 5d*" OR euroqol* OR sf-6d* OR sf6d* OR "short form 6-d" OR "quality of well-being" ) OR TITLE-ABS-KEY ( 15-d OR "health utilities index" OR chu-9d OR "child health index" OR aqol OR "adult quality of life" OR "eortc qlq c30" ) OR TITLE-ABS-KEY ( "preference base*" OR ( ( "quality of life" OR qol ) AND "self report*" ) OR "preference weight*" OR "health utilit*" OR "valuation weight*" OR "preference value*" OR "quality adjusted life year*" OR "qaly weight*" ) OR TITLE-ABS-KEY ( ( "health state*" W/5 ( valu* OR rank* OR preference* ) ) OR ( hr-pro OR hrpro OR hr-pro ) ) ) ) ) AND ( ( TITLE-ABS-KEY ( ( sami OR saami OR sampi OR samis OR lapps OR lappish OR lapp ) ) OR ( TITLE-ABS-KEY ( ( urban W/3 ( indian* OR native* OR aboriginal* ) ) ) ) OR ( TITLE-ABS-KEY ( "country food*" OR "residential school*" ) OR TITLE-ABS-KEY ( ( native* W/1 ( man OR men OR women OR woman OR boy* OR girl* OR adolescent* OR youth OR youths OR person* OR adult OR people* OR indian* OR nation OR tribe* OR tribal OR band OR bands ) ) ) OR TITLE-ABS-KEY ( "alaska native*" OR "hawiian islander*" ) ) OR ( TITLE-ABS-KEY ( metis OR "red road" OR "on reserve" OR "off-reserve" OR shaman* OR "traditional heal*" OR "traditional food*" OR "medicine man" OR "medicine woman" OR autochtone* ) ) OR ( TITLE-ABS-KEY ( maori OR "tangata whenua" OR mauori OR moriori OR mauri OR "torres strait islander*" OR aboriginie ) ) OR ( TITLE-ABS-KEY ( "first nation" OR "first nations" OR "first people*" OR "original people*" OR "first inhabitant*" OR "original inhabitant*" OR autochtone OR aborigin* OR indigenous OR metis "american indian*" OR "native american*" OR amerindian OR athapaskan OR saulteaux OR wakashan OR cree OR dene OR inuit OR inuk OR inuvialuit* OR haida OR ktunaxa OR tsimshian OR gitsxan OR nisga'a OR haisla OR heiltsuk OR oweenkeno OR kwakwaka'wakw OR "Nuu chah nulth" OR tsilhqot'in OR dakelh OR wet'suwet'en OR sekani OR dunne-za OR dene OR tahltan OR kaska OR tagish OR tutchone OR nuxalk OR salish OR stl'atlimc OR nlaka'pamux OR okanagan OR "Sec wepmc" OR tlingit OR anishinaabe OR blackfoot OR nakoda OR tasttine OR "Tsuu T'inia" OR gwich'in OR han OR tagish OR tutchone OR algonquin OR nipissing OR ojibwa OR potawatomi OR innu OR maliseet OR mi'kmaq OR micmac OR passamaquoddy OR haudenosaunee OR cayuga OR mohawk OR oneida OR onodaga OR seneca OR tuscarora OR wyandot OR "Six Nations" OR ( saami OR sampi OR ( sami AND NOT ulus ) OR samis OR southernsami* OR umesami* OR pitesami* OR lulesami* OR northernsami* OR enaresami* OR kolasami* OR lapp OR lapps OR lappish OR lappland OR ( lapland* AND NOT longspur ) OR lappalainen* OR saamelainen* OR "reindeer herd*" OR "reindeer culture*" OR "reindeer pastoral*" OR lappbys OR samebys OR reinbeitesdistrikt OR paliskunta OR siida ) OR "A'ani" OR haaninin OR atsina OR "Gros Ventre" OR acopsel OR tlacopsel OR lacopsel OR ahtna OR ahtena OR akenitsi OR occaneechi OR akokisa OR horcoquisa OR orcoquizas OR aleut OR unangax OR unangan OR alibamu OR "Alabama Alsea" OR alutiiq OR sugpiag OR "Pacific Yupik" OR amahami OR awaxawi OR androscoggin OR arosaguntacook OR ameriscoggin OR anishinaabeg OR chippewa OR anihsinape OR saulteaux OR apalachee OR aranama OR "Texan Coahuilteca" OR tamique OR arikara OR sahnish OR ree OR arickaree OR adakadaho OR assiniboine OR hohe OR nakota OR nakoda OR nakona OR "Atsa Kudok-wa" OR awatixa OR bannock OR "Snake Indians" OR bidai OR ishak OR quasmigdo OR biloxi OR "Blackfoot Confederacy" OR niitsitapi OR sikasikaitsitapi OR cahto OR kato OR kaipomo OR cahuilla OR ivilyuqaletem OR ivilyuat OR catawba OR inna OR iswa OR chemehuevi OR chickasaw OR "Chilula Chimakum" OR aqokulo OR chimariko OR chiricahua OR tsokanende OR chitimacha OR chetimachan OR sitimacha OR chowanoke OR roanoke OR chumash OR ciboney OR "Taino Ciwat" OR clatsop OR coos OR coosa OR uchis OR chiaha OR coste OR tali OR talisi OR coquille OR kokwell OR coso OR cowlitz OR taitnapam OR "Crow Nation" OR absaroka OR "Cui Ui Ticutta" OR cupeno OR kuupangaxwichem OR cupa OR "Cup' ig" OR nunivak OR "Dakota Oyate" OR lakota OR nakota OR santee OR teton OR sioux OR deadose OR "Deng Xinag" OR "Deg Xit' an" OR kaiyuhkhotana OR "Deg Hit' an" OR "Dena' ina" OR tanaina OR "Dichinanek' Hwt' ana" OR "Upper Kuskokwim" OR athabascan* OR kolchan OR goltsan OR "Tundra Kolosh" OR "Do lkabya" OR "Western Yavapai" OR duwamish OR esselen OR eyak OR "Gidi' tikadii" OR guwevkabaya OR "Southeast Yavapai" OR "Gwich' in" OR kutchin OR haida OR xaadas OR xaat OR halchidhoma OR havasupai OR "Green Water People" OR hiratsa OR hiraaca OR ho-chaaqa OR winnebago OR holikachuk OR innoko OR "Tlegon-khotana" OR hopi OR "Houma Louisiana" OR huaco OR waco OR hualapai OR hupa OR natinixwe OR natinook-wa OR "Hwech' in" OR hankutchin OR "Iroquois Confederacy" OR "Hodinoso ni" OR "Illinois Confedera*" OR ilinoweg OR illini OR inupiat OR ioway OR baxoje OR jicarilla OR juaneno OR acjachemen OR jumano OR kalapuya OR clackama OR kalispel OR "Pend d' Oreilles" OR qlispe OR karuk OR karok OR chum-ne OR katkoc OR kaw OR kansa OR kanza OR kawaiisu OR nuwa OR kennebec OR "Kinipekw Kittitas" OR awi-adshi OR mahane OR wahnookt OR "Koa' aga' itoka" OR keresan OR kichai OR kitsai OR keechi OR "K' itaish" OR kiowa OR gaigwu OR cauigu OR kutjau OR kwu-da OR tep-da OR kitanemuk OR klickitat OR "Qwu' ih-hwai-pum" OR konkow OR "Koop Ticutta" OR koyukon OR ktunaxa OR kootenai OR flathead OR kucadikadi OR "Kotsa' va" OR kumeyaay OR tipai-ipai OR kamia OR diegueno OR kwapa OR cocopah OR cucapa OR "Xawitt kwnchawaay" OR lassik OR lenape OR leni-lenape OR lipan OR luiseno OR payomkawichum OR madqwadabaya OR "Desert Yavapai" OR mahican OR mohicans OR makah OR makuhadokado OR maliseet OR wolistoqiag OR manahoac OR mahock OR meipontsky OR mandan OR mattole OR "Bear River" OR "Tul' bush" OR "Ni' ekeni" OR meherrin OR menominee OR mackinac OR mescalero OR myaamiaki OR kickapoo OR twigtwee OR missouria OR miwok OR miwuk OR moadokado OR modoc OR mohave OR "Aha Makhav" OR mohawk OR "Kaneng' hega" OR molala OR molale OR molele OR mono OR nyyhmy OR moosonee OR "Moose Cree" OR monsonis OR multnomah OR chinook OR nabedache OR nabaydacu OR wawadishe OR nabiltse OR applegate OR dakubetede OR "Nacho Nyak Dun" OR tutchone OR nacono OR "Na' isha" OR nanticoke OR navajo OR ndee OR nial OR niimiipu OR "Nez Perce" OR watapala OR watapahlute OR nisenan OR nisqually OR nomlaki OR noamlakee OR "Central Wintun" OR nongatl OR nottoway OR cheroenhaka OR "Northern Cheyenne" OR ohlone OR costanoan OR omaha OR "O' odham" OR pima OR "Tahono O' odham" OR papago OR osage OR otoe OR otse OR "Ozav Dika" OR palus OR passamaquoddy OR pestomuhkati OR patiri OR petaros OR pastia OR patwin OR "Southern Wintun" OR pawnee OR panis OR skidi OR pedee OR penobscot OR "Petun Piipaash" OR "Kokmalik' op" OR piscataway OR piscatawa OR doeg OR conoy OR "Pit River" OR pomo OR kashaya OR ponca OR ponka OR pottawatomi OR bodewadmik OR powhatan OR puyallup OR spuyalepabs OR quapaw OR ugahxpa OR quechan OR yuma OR kwtsaan OR quileute OR salinan OR saponi OR monacan OR sapon OR "Eastern Blackfoot" OR christanna OR sawawatodo OR serrano OR taaqtam OR "Maarenga' yam" OR yuhaviatam OR shasta OR chasta OR sasti OR shoshone OR siletz OR sinkine OR sinkyone OR "Siuslaw Umpqua" OR skitswish OR "Coeur D' Alene" OR "Schitsu' umash" OR snohomish OR snuqualmi OR sokoki OR missiquoi OR stillaguamish OR stoluckwamish OR suquamish OR sutaio OR swinomish OR skagit OR syilx OR okanagan OR sotaae OR "Taga Ticutta" OR takelma OR dagelma OR taltushtuntede OR galice OR "Gwich' in" OR taos OR taovaya OR tataviam OR alliklik OR tawakoni OR tahuacano OR tenino OR thawikila OR hathawekela OR " Fort Ancient" OR tigua OR "Ysleta del Sur" OR tillamook OR nehalem OR timbisha OR panamint OR timpanogos OR ute OR tlingit OR "Toy Ticutta" OR tolowa OR "Talawa Dini'" OR tongva OR gabrieleno OR fernandeno OR tobikhar OR tonkawa OR ticanwatic OR tsikip OR appalousa OR opelousa OR tsitsistas OR tubatulabal OR tukabatchee OR tula OR tunica OR tuscarora OR tomahittan OR kuskarawock OR tutelo OR tutero OR totteroy OR tutera OR yusan OR tututni OR umatilla OR umpqua OR waccamaw OR waxmaw OR wadatika OR "Harney Valley Paiute" OR wailiki OR waluulapam OR "Walla Walla" OR walpapi OR huipui OR wampanoag OR massasoit OR wanapum OR wappo OR washoe OR wichita OR willapa OR kwalhioqua OR "Wi pukba" OR "Verde Valley Yavapai" OR wintu OR "Northern Wintun" OR wiyot OR "Wee' at" OR weyet OR yakama OR "Yamosopo Tuviwarai" OR yaqui OR yoeme OR yatasi OR yattasih OR yavbe' OR "Northwest Yavapai" OR yojuane OR yokuts OR mariposa OR yuki OR yupighyt OR yup'ik OR yurok OR olekwo'l OR zuni ) ) ) AND NOT TITLE-ABS-KEY ( ( ( hui OR han ) W/3 ( qinghai OR tibet* OR chinese ) ) OR ( hui W/22 meeting* ) OR ( hui W/2 gathering* ) OR animal* ) ) AND ( LIMIT-TO ( PUBYEAR , 2021 ) OR LIMIT-TO ( PUBYEAR , 2020 ) )

**PROQUEST Dissertations and Theses Searched May 11, 2021 Results = 28**

(noft(("time trade-off" OR HR-PRO OR HRPRO OR HR-PRO OR (TTO NOT "tea tree") OR "standard gamble" OR "health utility" OR eq-5d* OR eq5d* OR euroqol-5d OR sf-6d OR sf6d* OR hui2 OR hui3 OR "hui 2" OR "hui 3" OR short form 6-d OR QWB OR "quality of well-being" OR 15-d OR "health utilities index" OR CHU-9D OR "child health index" OR AQoL OR "adult quality of life" OR "eortc qlq c30") OR "preference based measure") OR (noft(instrument*) OR noft(measure*) OR noft("classification system") OR noft("classification systems") OR noft("health state classification") OR noft("descriptive system") OR noft("descriptive systems") OR noft(index) OR noft(indices) OR noft(indexes) OR noft(tool) OR noft(tools) OR noft(questionnaire*) OR noft(scale) OR noft(scales)) AND (noft(preference*) OR noft(utility) OR noft(utilities) OR noft("valuation weight*") OR noft("qaly weight") OR noft("health state") OR noft("health states") OR noft(value) OR noft(values) OR noft(rank*)) AND (noft("quality of life") OR noft(qol) OR noft(hrqol) OR noft(hrql) OR noft("health status") OR noft("quality adjusted life year*") OR noft(qaly*)) NOT noft(animal*) OR ti(china) OR noft(ti) (noft(hui W3) (noft(meeting) OR noft(gathering) OR noft(qinghai) OR noft(tibet*) OR noft(chinese)) OR noft(ti) (noft(han w3 quinghai) OR noft(tibet*) OR noft(chinese)))) AND (ab((indigenous* OR amerindian* OR autochtone* OR "first nation" OR "first nations" OR metis OR inuit OR innu OR inuk OR inuvialuit OR aborigin* OR sami OR sampi OR saami OR Lapp OR Lapps OR Maori OR "torres strait islander*" OR "american indian*" OR "hawaiian islander*" OR "alaska* native*" OR "on reserve" OR "off reserve" OR "Indian W3 reservation*" OR "red road" OR "residential school*" OR "country food*" OR "medicine man" OR "medicine woman" OR (urban W3 (indian* OR native*)) OR (native* w3 (man OR men OR women OR woman OR youth* OR child* OR elder OR elders OR tribe* OR tribal OR band OR bands OR settlement* OR reserve* OR reservation*)))) OR ti((indigenous* OR amerindian* OR autochtone* OR "first nation" OR "first nations" OR metis OR inuit OR innu OR inuk OR inuvialuit OR aborigin* OR sami OR sampi OR saami OR Lapp OR Lapps OR Maori OR "torres strait islander*" OR "american indian*" OR "hawaiian islander*" OR "alaska* native*" OR "on reserve" OR "off reserve" OR "Indian W3 reservation*" OR "red road" OR "residential school*" OR "country food*" OR "medicine man" OR "medicine woman" OR (urban W3 (indian* OR native*)) OR (native* w3 (man OR men OR women OR woman OR youth* OR child* OR elder OR elders OR tribe* OR tribal OR band OR bands OR settlement* OR reserve* OR reservation*)))))

**Prospero Searched May 11, 2021**

Line Search for Hits

#1 indigenous* OR amerindian* OR autochtone* OR "first nation" OR "first nations"

OR metis inuit OR innu OR inuk OR inuvialuit OR aborigin* OR sami OR sampi OR saami

OR Lapp OR Lapps OR Maori OR "torres strait islander*" OR "american indian*" OR

"hawaiian islander*" OR "alaska* native*" OR "on reserve" OR "off reserve" OR

"Indian W3 reservation*" OR "red road" OR "residential school*" OR "country food*"

OR "medicine man" OR "medicine woman" OR "urban indian*" OR "urban native*" OR

"native* elder*" OR tribe* OR tribal or "Indian band*" OR "native band*" or

"native settlement*" OR "Indian reservation*" 1107

#2 "preference based*" 117

#3 "quality of well-being" or "15-d" or "health utilities index" or "CHU-9D"
or "child health index" or AQoL or "adult quality of life" or "eortc qlq c30" 326

#4 "eq-5d-3L" or "eq-5d-5L" or "euroqol-5d" or "sf-6d" or "short form 6-d" 309

#5 "standard gamble" or "health utility" or QWB 149

#6 "time trade-off" or (tto not "tee tree") 144

#7 #6 OR #5 OR #4 OR #3 OR #2 801

#8 #1 AND #7 3

**Description of Author Position**

The primary author (LMR) is a PhD student exploring this topic in the early phases of doctoral study. During this work, she has lived and worked as a nurse, educator, and researcher in Una’maki (known by its settler name as Cape Breton) and also lived and studied part-time in Treaty 6 Territory (Edmonton). She identifies as a white female of mixed Scottish, English, and French settler ancestry who grew up in Mi’kmaki (Nova Scotia). She has worked as a non-Indigenous nurse in communities across Canada and the Western Arctic. Lilla has experience in community-based research and research partnering with and supporting needs of underserved communities. Her PhD research has been motivated by work in these settings and the current need to support population-appropriate approaches to measuring HRQL.

The second author (AN) is an experienced federal public servant and resource professional (CPA, CPHR) who has undertaken a course of PhD studies in health services and policy research focusing on health economics as a personal response to the Truth and Reconciliation Calls to Action in Health. As a first generation Canadian of Irish and British origin he is conscious of his own Western bias and the legacy impacts of colonial policy upon Indigenous health outcomes. The third author (KS) is a research assistant with experience working as a registered nurse in Indigenous communities. She is from One Arrow First Nation (Saskatchewan) and is currently a Master of Education student. The fourth author (EJA) is on their Elder journey and advising and working with the research team going forward. EJA and KS joined the research team later in the project (July, 2022 and March 2023, respectively) and will be working with the research team moving forward on projects that have arisen from this review. The fifth author (SMC) is a non-Indigenous librarian experienced in systematic reviews. The sixth author (SC) is a non-Indigenous senior researcher with extensive experience in health systems and Indigenous health research in northern and remote community contexts. The seventh author (FAS) is a non-Indigenous senior health outcomes researcher with experience in patient-reported outcomes and measurement of health-related quality of life. The last author (JAJ) is a non-Indigenous senior researcher with extensive experience in epidemiology, pharmacoepidemiology, economic evaluation, and measurement of health-related quality of life. FAS and JAJ are also very experienced in partnership with clinical, provincial, and national government and non-government agencies.

Table I Characteristics of Included Reports

| **First author** | **Year** | **Country** | **Disease or condition of interest** | **Indigenous group** | **How ethnicity was determined?** | **Ethically engaged approach?** | **Was the focus of the PBM primary or secondary?** | **Results reported for Indigenous people or subgroup?** | **Direct or indirect PBM?** | **If indirect, was it generic or condition specific PBM?** | **Instrument or method** | **Visual Analog Scale (VAS) used?** | **Value set** | **Was formal or informal translation used? If so, to/from which language?** | **Was this reporting development of an instrument? If so, for which group?** |  |
| --- | --- | --- | --- | --- | --- | --- | --- | --- | --- | --- | --- | --- | --- | --- | --- | --- |
| **Reports of Measurement of Health-Related Quality of Life** | | | | | | | | | | | | | | | | |
| Altomare | 2018 | USA^3^ | Polycythemia vera | Native American, Pacific Islander | NR | NR | Unclear | No | Indirect | Condition specific | EORTC-QLQ-C30 | NR | NA | NR | No |  |
| Armstrong | 2021 | Australia | Acute stroke or traumatic brain injury (TBI) | Aboriginal | Medical file or self-identified | Yes | Primary | Yes | Indirect | Generic | EQ-5D-3L | Yes (0-100) | NR | NR | No |  |
| Barnabe | 2015 | Canada | Rheumatoid arthritis | Aboriginal Albertans | NR | NR | Unclear | Yes | Indirect | Generic | EQ-5D | NR | NA | NR | No |  |
| Barnabe | 2018 | Canada | Rheumatoid arthritis refractory to DMARDS and initial biologic therapy | First Nations, Inuit, Metis people | Self-report | Unclear | Primary | Yes | Indirect | Generic | EQ-5D | Yes (0-10) | Canada | NR | No |  |
| Banham | 2019 | Australia | Health related quality of life | Aboriginal South Australians | National best practice guidelines | NR | Primary | Yes | Indirect | Generic | SF-6D v2 | NR | UK | NR | No |  |
| Chenhall | 2012 | Australia | Alcohol and drug rehabilitation | Indigenous Australians | NR | Yes | Primary | Yes | Direct | Generic | SEIQoL-DW^1^ |  | Indiivdual values | NR | No |  |
| Cousins | 2021 | New Zealand | Chronic health conditions | Maori and Pacific peoples | NR | Yes | Secondary | Unclear | Indirect | Generic | EQ-5D-5L | NR | New Zealand | NR | No |  |
| Custer | 2014 | USA | Viral infections | American-Indian | NR | NR | Primary | Yes | Indirect | Generic | EQ-5D | Yes (0-100) | USA | NR | No |  |
| Delbaere | 2021 | Australia | Healty ageing | Aboriginal and Torres Strait Islander | Self-identified | Yes | Primary | Yes | Indirect | Generic | EQ-5D-5L | NR | NR | NR | No |  |
| Derrett | 2009 | New Zealand | Disability outcomes of injury | Maori | NR | Yes | Primary | Yes | Indirect | Generic | EQ-5D | NR | New Zealand | Informal (Maori) | No |  |
| Derrett | 2011 | New Zealand | Disability outcomes of injury | Maori | NR | NR | Primary | No | Indirect | Generic | EQ-5D | Yes | NR | Informal (Maori) | No |  |
| Derrett | 2012 | New Zealand | Spinal cord injury | Maori, Pacific | Clinical records | NR | Secondary | Yes | Indirect | Generic | EQ-5D-3L | NR | NR | NR | No |  |
| Derrett | 2017 | New Zealand | People receiving long-term dialysis; patient-reported global health outcomes | Maori, Pacific | Self-report | NR | Secondary | Yes | Indirect | Generic | EQ-5D-3L | NR | NR | NR | No |  |
| Dingwall | 2019 | Australia | End stage kidney diseae (ESKD), hemodialysis | Indigenous Australians | NR | NR | Secondary | Yes | Indirect | Generic | EQ-5D-5L | NR | NR | Unclear (Options for 11 Northern Territory Indigenous languages) | No |  |
| Dingwall | 2020 | Australia | End stage kidney disease (ESKD), haemodialysis | Indigenous Australians | NR | NR | Unclear | Yes | Indirect | Generic | EQ-5D | NR | NR | NR | No |  |
| Dingwall | 2021 | Australia | End stage kidney diseae (ESKD), hemodialysis | Indigenous Australians | NR | Yes | Secondary | Yes | Indirect | Generic | EQ-5D | Yes | NR | Unclear (Options for 11 Northern Territory Aboriginal languages) | No |  |
| Donaldson | 2022 | Australia | Septic shock | Aboriginal and Torres Strait Islander | Medical records | Yes | Secondary | Yes | Indirect | Generic | EQ-5D-5L | Yes | NR | NR | No |  |
| du Toit | 2016 | South Africa | Cervical cancer | South Africans | Self-report | NR | Primary | Yes | Indirect | Condition specific | EORTC-QLQ-C30 | NR | NR | Formal (Xhosa, Afrikaans, plus English option) | No |  |
| Farace | 2014 | USA | Malignant glioma | Native American | NR | NR | Primary | No | Indirect | Condition specific | EORTC-QLQ-C30 | NR | NR | NR | No |  |
| Gall | 2022 | Australia | Impact of Covid-19 pandemic | Aboriginal and Torres Strait Islander | NR | Yes | Secondary | Yes | Indirect | Generic | AQ0L-4D | Yes (0-100) | NR | NR | No |  |
| Garvey | 2014 | Australia | Cancer (multiple types and stages) | Indigenous Australian | NR | NR | Secondary | Yes | Indirect | Generic | AQoL-4D | NR | NR | NR | No |  |
| Garvey | 2016 | Australia | Cancer | Indigenous Australian | Self-identified | NR | Primary | Yes | Indirect | Generic | AQoL-4D | NR | NR | NR | No |  |
| Groessl | 2003 | USA | Osteoarthritis | Native American | NR | NR | Primary | No | Indirect | Generic | QWB | NR | Unclear | NR | No |  |
| Guevara | 2020 | Equador | MSK pain and rheumatic disease | Saraguro | Self-identified | Yes | Secondary | Yes | Indirect | Generic | EQ-5D-3L | Yes (0-100) | Unclear | NR | No |  |
| Hachem | 2021 | Australia | Type 2 diabetes | Indigenous Australians | NR | NR | Secondary | Yes | Indirect | Generic | EQ-5D-5L | NR | NR | NR | No |  |
| Harcombe | 2022 | New Zealand | Musculoskeletal injury | Maori | NR | Unclear | Secondary | Yes | Indirect | Generic | EQ-5D-5L | NR | NR | NR | No |  |
| Hatcher | 2011 | New Zealand | Self-harm | Maori | Unclear | Yes | Secondary | Yes | Indirect | Generic | EQ-5D | NR | NR | Informal | No |  |
| Hay | 2021 | USA | Covid-19 | Native Hawaiian/Pacific Islander, American Indian/Alaskan Native | Unclear | NR | Primary | Yes | Indirect | Generic | EQ-5D-5L | Yes (0-100) | USA | NR | No |  |
| Hughes | 2004 | South Africa | HIV | Zhosa | NR | NR | Primary | Yes | Indirect | Generic | EQ-5D | Yes | NR | Unclear (Zhosa) | No |  |
| Ingham | 2017 | New Zealand | Asthma wheeze | New Zealand Maori | Self/parental report | Yes | Secondary | Yes | Indirect | Generic | EQ-5D-Y (Proxy 1 version) | NR | New Zealand | NR | No |  |
| Jamieson | 2022 | Australia | Dental caries | Indigenous children | NR | NR | Secondary | Yes | Indirect | Generic | CHU-9D | NR | NR | NR | No |  |
| Janda | 2009 | Australia | Cancer screening and risk behaviors | Indigenous Australian | Self-report | NR | Primary | No | Indirect | Condition specific | FACT-GP | NR | NR | NR | No |  |
| Johnson | 2015 | Australia | Chronic disease management | Aboriginal and Torres Strait Islander | Self-report | Yes | Secondary | Yes | Indirect | Generic | AQoL | NR | NR | NR | No |  |
| Ju | 2021 | Australia | Oral health | Indigenous Australian | Self-report | Yes | Primary | Yes | Indirect | Generic | EQ-5D-5L | NR | UK | No | No |  |
| Kilkenny | 2018 | Australia | Admitted patients with stroke or TIA | Aboriginal Australians | NR | NR | Primary | Yes | Indirect | Generic | EQ-5D-3L | NR | NR | NR | No |  |
| Kinchin | 2018 | Australia | Multiple chronic conditions and emerging complex care needs | Aboriginal and Torres Strait Islander | NR | NR | Secondary | NR | Indirect | Generic | EQ-5D-5L; AQOL-8D | NR | NR (EQ-5D-5L); Australia general population (AQOL) | NR | No |  |
| Kularatna | 2020 | Australia | Oral health | Indigenous Australian | NR | Yes | Secondary | Yes | Indirect | Generic | CHU-9D | NA | Australia | NR | No |  |
| LaGrappe | 2022 | Australia | Machado-Joseph Disease (MJD) / Spinocerebellar Ataxia Type 3 (SCA3); Sleep disorders | Aboriginal Australians | NR | Yes | Secondary | Yes | Indirect | Generic | EQ-5D | NR | NR | Unclear (recruited "3 Aboriginal language groups") | No |  |
| Lalloo | 2015 | Australia | Denal caries | Indigenous Australian children | NR | Unclear | Secondary | Yes | Indirect | Generic | CHU-9D | NR | UK | NR | No |  |
| Lapsley | 2020 | New Zealand | Caregiving | Maori | Supported by the New Zealand electoral roll | Yes | Secondary | Yes | Indirect | Generic | EQ-5D-3L | NR | NR | NR | No |  |
| Lavergne | 2009 | Canada | NR | Aboriginal (Canada) | CCHS | NR | Primary | Yes | Indirect | Generic | HUI3 | NR | NR | NR | No |  |
| Lavergne | 2012 | Canada | Rural health | First Nations, Inuit, or Metis | NR | NR | Primary | Yes | Indirect | Generic | HUI3 | NR | NR | No | No |  |
| Liu | 2010 | Australia | Cardiovascular disease | Indigenous Australian | NR | Yes | Secondary | Yes | Indirect | Generic | EQ-5D | NR | NR | NR | No |  |
| Lott | 2019 | Australia | Surgery | Aboriginal and Torres Strait Islander | NR | NR | Secondary | NR | Indirect | Generic | EQ-5D | NR | NR | No | No |  |
| Maclennan | 2022 | New Zealand | Injury | Maori | NZ 2006 Census ethnicity question | NR | Primary | Yes | Indirect | Generic | EQ-5D-3L | NR | NR | NR | No |  |
| Mann | 2020 | Australia | complex care/needs | Indigenous Australian | NR | NR | Primary | Yes | Indirect | Generic | EQ-5D | Yes | NR | NR | No |  |
| McDermott | 2015 | Australia | Type 2 diabetes | Aboriginal and Torres Strait Islander | Health records | Unclear | Secondary | Yes | Indirect | Generic | AQoL | NR | NR | Informal (Creole) | No |  |
| McNoe | 2019 | New Zealand | End stage kidney diseae (ESKD) | Maori, Pacific People | Self-reported ethnicity (per census data) | NR | Unclear | Yes | Indirect | Generic | EQ-5D-3L | NR | NR | Informal (Unclear) | No |  |
| Mold | 2004 | USA | Peripheral neurological deficits | Native American | Self-report (questionnaire) | NR | Secondary | No | Indirect | Generic | HUI3; QWB | NR | NR | NR | No |  |
| Moodie | 2010 | Australia, New Zealand, Fiji, Tonga | obesity | Multiple (Maori, Pacific Islanders, Indigenous Fijians, Tongans, and unspecified "Australians") | NR | NR | Primary | Yes | Indirect | Generic | AQoL-6D | NR | NR | NR | No |  |
| Morales | 2018 | Mexico | Rheumatoid Arthritis | Maya-Yucateco Indigenous | NR | NR | Secondary | Yes | Indirect | Generic | EQ-5D-3L | NR | NA | NR | No |  |
| Oen | 2018 | Canada | Juvenile Idiopathic Arthritis (JIA) | Canadian Aboriginal | NR | NR | Primary | Yes | Indirect | Generic | QoML | Yes (0-10) | Indiivdual values | NR | No |  |
| Radford | 2019 | Australia | Multiple (Poor mobility, cognitive decline, depression, chronic disease, falls, physical Inactivity) | Aboriginal and Torres Strait Islander | Self-identified | NR | Secondary | Yes | Indirect | Generic | EQ-5D | NR | NR | NR | No |  |
| Ramirez-Cervantes | 2015 | Mexico | Celiac disease (CD) | Mexican Mestizo | NR | NR | Primary | Yes | Indirect | Generic | EQ-5D-3L | Yes | NR | Formal (validated Spanish version) | No |  |
| Ralph-Campbell | 2006 | Canada | Type 2 diabetes | Aboriginal | Self-report | NR | Unclear | Yes | Indirect | Generic | HUI-3 | NR | NR | NR | No |  |
| Ranta | 2021 | New Zealand | Stroke care, rurality, ethnicity | Maori | NR | NR | Secondary | Yes | Indirect | Generic | EQ-5D-3L | NR | NR | NR | No |  |
| Robinson | 2022 | Australia | Cancer | Aboriginal | NR | Unclear | Primary | Yes | Indirect | Generic | AQoL-4D | NR | Unclear | NR | No |  |
| Sato | 2012 | Ghana | Use of traditional medicine | Multiple traditional groups identified through religion (traditionalist African or Indigenous religion) or other ethnicities | Self-report | NR | Secondary | No | Indirect | Generic | EQ-5D-3L | Unclear | NR | Informal (Multiple Ghanaian dialects - Ga, Twi, Waale, Dagaare) | No |  |
| Scholes-Robertson | 2021 | Australia | Chronic kidney disease (CKD) | Aboriginal Australian and/or Torres Strait Islander | Self-report | NR | Secondary | Unclear | Indirect | Generic | EQ-5D | NR | NR | NR | No |  |
| Segal | 2016 | Australia | Type 2 diabetes | Indigenous Australian | NR | Unclear | Secondary | Yes | Indirect | Generic | AQoL-4D | NR | Australia general population | NR | No |  |
| Shettigar | 2021 | New Zealand | Kidney failure | Maori and Pacific peoples | Self-report (interview) | Unclear | Secondary | Yes | Indirect | Generic | EQ-5D-3L | NR | NR | NR | No |  |
| Smith | 2019 | Australia | Dementia | Aboriginal Australian and/or Torres Strait Islander | NR | Unclear | Primary | Yes | Indirect | Generic | EQ-5D-5L | NR | NR | NR | No |  |
| Taylor | 2005 | New Zealand | MSK pain | Maori | NR | NR | Secondary | Yes | Indirect | Generic | EQ-5D | NR | NA | NR | No |  |
| Thompson | 2022 | New Zealand | Stroke | Maori and Pacific peoples | Self-identified based on Statistics New Zealand coding | NR | Unclear | Yes | Indirect | Generic | EQ-5D-3L | Yes | NR | NR | No |  |
| Toombs | 2018 | Australia | Depression | Aboriginal and Torres Strait Islander | NR | Yes | Secondary | Yes | Indirect | Generic | AQoL-8D | NR | NR | NR | No |  |
| Walker | 2019 | New Zealand | Smoking | New Zealand Maori | Self-identified | Yes | Secondary | Yes | Indirect | Generic | EQ-5D | NR | New Zealand | NR | No |  |
| Walker | 2021 | New Zealand | Smoking | Maori or whanau of Maori | Self-identified | NR | Secondary | Yes | Indirect | Generic | EQ-5D | NR | New Zealand | NR | No |  |
| Wilson | 2010 | USA | Type 2 diabetes | American Indian | NR | Yes | Secondary | Yes | Indirect | Generic | QWB | NR | NR | NR | No |  |
| **Reports of Instrument Development or Translation** | | | | | | | | | | | | | | | | |
| Anderson | 2021 | Australia | Well-being | Aboriginal and Torres Strait Islander | NR | Yes | Primary | Yes | NA | NA | NA | NA | NA | NR | Yes, Aboriginal and Torres Strait Islander |  |
| Arrow | 2018 | Australia | Dental caries | Aboriginal Australians | Community engagement | Yes | Secondary | Yes | Indirect | Both | CHU-9D, EQ-5D-Y, new oral specific health utility scale | NR | NR | NR | Yes, oral health specific multi-attribute utility instrument for Aboriginal children |  |
| Howard | 2020 | Australia | Well-being | Aboriginal and Torres Strait Islander | NR | Yes | Primary | Yes | NA | NA | NA | NA | NA | NR | Yes, Aboriginal and Torres Strait Islander |  |
| Nagel | 2020 | Australia | Chronic kidney disease | Aboriginal and Torres Strait Islander | Self-identified | Yes | Secondary | Yes | Indirect | Generic | EQ-5D-3L | NR | NR | Formal (multiple languages) | No |  |
| Willing | 2020 | New Zealand | Health | Maori | NR | Yes | Primary | Yes | NA | NA | NA | NA | NA | NR | Yes, Maori |  |
| **Reports of Direct Preference Elicitation** | | | | | | | | | | | | | | | | |
| Alfonso | 2022 | USA | Suicidal ideation and depression | American Indian/Alaskan Natives | NR | Yes | Primary | Yes | Direct |  | VAS (0-100) | Yes | NA | NR | No |  |
| Devlin | 2000 | New Zealand | General population | Maori | NR | NR | Primary | Yes | Indirect | Generic | EQ-5D-3L | Yes (0-100) | Unclear | NR | No |  |
| Ju | 2021 | Australia | HPV and cervical cancer | Indigenous Australians | NR | Yes | Primary | Yes | Direct | NA | Standard Gamble | No | NA | NR | No |  |
| Ju | 2021 | Australia | HPV and oropharyngeal cancer | Indigenous Australians | NR | Yes | Primary | Yes | Direct | NA | Standard Gamble | No | NA | NR | No |  |
| **Reports of Instrument Performance** | | | | | | | | | | | | | | | | |
| McClure | 2011 | USA | under-corrected refractive error (vision problem) | American Indian/Alaskan Natives | NR | NR | Primary | Yes | Indirect | Condition specific | NEI-VFQ-25 | NR | NA | NR | No |  |
| Jelsma | 2004 | South Africa | General population and rehabilitation | Zhosa | NR | NR | Primary | Yes | Indirect | Generic | EQ-5D | Yes (pain only) | NA | Formal (Zhosa) | No |  |
| Perkins | 2004 | New Zealand | HRQL | Maori | NR | Yes | Primary | Yes | Both | Generic | EQ-5D-3L (indirect); VAS (0-100) (Direct) | Yes | NR | NR | No |  |
| Ribeiro-Santiago | 2021 | Australia | Oncogenic genotypes of HPV infection | Aboriginal and Torres Strait Islander | NR | Yes | Primary | Yes | Indirect | Generic | EQ-5D-5L | NR | NR | NR | No |  |

^1^ SEIQoL-DW = Self Evaluated Individual Quality of Life-Direct Weight; ^2^ QOML = Quality of My Life Questionnaire (QoML; ^3^ USA = United States of America

**Table II Performance of PBMs with Indigenous People**

| **Author (Year)** | **Sample (n) (% Indigenous)** | **PBM** | **Measurement properties of interest** | **Validity findings** | **Reliability findings** | **Responsiveness findings** | **Methodologic observations** | **Major findings related to Indigenous people** |
| --- | --- | --- | --- | --- | --- | --- | --- | --- |
|  |  |  |  | **Content, Construct, & Criterion validity** | **Reliability (test-retest), Internal consistency, & Measurement error** | **Responsiveness** |  |  |
| **Jelsma (2004)**  **[42]** | Reliability (n=88); Validity (n=49) (100%) | EQ-5D-3L | Test-retest reliability, stability of VAS, concurrent validity | **Concurrent validity shown for:**  Mobility (vs velocity of gait) Self-care and usual activity (vs BADL instrument) Pain/discomfort (vs pain VAS) Anxiety/depression (vs BDI scores)  **"All domains performed well"** | **ICC for VAS^a^: significant and reasonably high**  (0.66) **ICC for domains: all significant but somewhat low** (Range = 0.39-0.75; Mobility 0.46; Self-care 0.69; Usual activity 0.40; Pain/discomfort 0.33 (low); Anxiety/derpession 0.42) | NR^b^ | NR | Zhosa EQ-5D is valid and reliable; Few gold standards with which to compare validity of EQ-5D in Zhosa; Recommend research on construct validity of the instruments used (concepts are different in Zhosa vs Western culture) |
| **McClure (2011)**  **[48]** | 102 (100%) | NEI-VFQ-25 | Reliability (Internal consistency of sub-scales) | NR | **Internal consistency of sub-scales: acceptable (**Cronbach's alpha = 0.94) | NEI-VFQ-25 was sensitive to changes in visual acuity.   *Ceiling effect | NR | Internal consistency of sub-scales acceptable for study population (American Indian/Alaska Natives)  NEI-VFQ-25 was sensitive to changes in visual acuity for study population (AI/AN) |
| **Perkins (2004)**  **[9]** | 66 (100%) | EQ-5D-3L | Content validity, construct validity, test-retest reliability | **Content valdiity: adequate** (Reported by 76%). | **ICC (as a measure of test-retest reliability): adequate (**0.85 for health state 11111); 0.96 for health state 33333)) | NR | 71% did not value dead.  Many asked for assistance when scoring other health states.   Logical inconsistencies (70%) and missing valuations noted (Figure 1 & Table 1). | Authors conclude that:  - Suggests the EQ-5D hasadequate content validity and test-retest reliability (per extreme states) - Most Maori do not conform to the ‘Maori health model’. - High prevalence of missing valuations and logical inconsistencies suggests the health state valuation instrument lacks construct validity. - Missing values for dead are especially problematic because this renders all of a respondent’s other valuations unusable for estimating ‘social’ tariffs (for calculating QALYs for CUA) |
| **Ribeiro-Santiago  (2021)**  **[53]** | 988 (100%) | EQ-5D-5L | Face and content validity, construct validity, criterion validity, reliability | **Face & content validity: endorsed by Indigenous Reference Group in the absence of another suitable instrument  Dimensionality: 2 dimensional - physiological + psychological** (exploratory graph analysis vs factor analysis, confirmed with CFA) **Corrected item-total correlation (CITC): moderate to strong (physiological) and strong (psychological)  Item analysis/performance (IRT Rasch model): adequancy of response categories and monoticity  Concurrent criterion validity: good** (AUROC values correctly identify poor general health and chronic pain at item and dimension/subscale level) **Discriminant validity: reasonable** (weak association between EQ-5D-5L items and subscale with experiences of racism at item and dimension/subscale level **Convergent validity: yes** (association between EQ-5D items and subscales with general health) | **Reliability (internal consistency): adequate** (Omega coefficient = good (0.84 physiological) and adequate (0.70 psychological)) | NR | NR | Psychometric properties are excellent; there are two dimensions (pshyiological and psychological), reliability is adequate. |

**^a^VAS = Visual Analogue Scale; ^b^NR = Not Reported;**
